# Inferring *who-infected-whom-where* in the 2016 Zika outbreak in Singapore—a spatio-temporal model

**DOI:** 10.1098/rsif.2018.0604

**Published:** 2019-06-19

**Authors:** Kiesha Prem, Max S. Y. Lau, Clarence C. Tam, Marc Z. J. Ho, Lee-Ching Ng, Alex R. Cook

**Affiliations:** 1 Saw Swee Hock School of Public Health, National University of Singapore and National University Health System, Tahir Foundation Building, 12 Science Drive 2, #10-01, Singapore 117549, Republic of Singapore; 2 Department of Ecology and Evolutionary Biology, Princeton University, Princeton, NJ 08544, USA; 3 London School of Hygiene and Tropical Medicine, Keppel Street, London WC1E 7HT, UK; 4 Ministry of Health, 16 College Road, Singapore 169854, Republic of Singapore; 5 Environmental Health Institute, National Environment Agency, 11 Biopolis Way, Singapore 138667, Republic of Singapore

**Keywords:** spatial models, temporal, Bayesian data augmentation, Zika, vector-borne outbreaks

## Abstract

Singapore experienced its first known Zika outbreak in 2016. Given the lack of herd immunity, the suitability of the climate for pathogen transmission, and the year-round presence of the vector—*Aedes aegypti*—Zika had the potential to become endemic, like dengue. Guillain–Barré syndrome and microcephaly are severe complications associated elsewhere with Zika and the risk of these complications makes understanding its spread imperative. We investigated the spatio-temporal spread of locally transmitted Zika in Singapore and assessed the relevance of non-residential transmission of Zika virus infections, by inferring the possible infection tree (i.e. *who-infected-whom-where*) and comparing inferences using geographically resolved data on cases' home, their work, or their home and work. We developed a spatio-temporal model using time of onset and both addresses of the Zika-confirmed cases between July and September 2016 to estimate the infection tree using Bayesian data augmentation. Workplaces were involved in a considerable fraction (64.2%) of infections, and homes and workplaces may be distant relative to the scale of transmission, allowing ambulant infected persons may act as the ‘vector’ infecting distant parts of the country. Contact tracing is a challenge for mosquito-borne diseases, but inferring the geographically structured transmission tree sheds light on the spatial transmission of Zika to immunologically naive regions of the country.

## 1. Introduction

The tropical city-state of Singapore is home to the *Aedes* mosquitoes, *Ae. aegypti* and *Ae. albopictus*, has local transmission of arboviruses such as dengue [1–5] and chikungunya [2,6,7] and is at risk of outbreaks of Zika virus (ZIKV) [8]. The anthropophilic *Ae. aegypti*, in particular, is found—at low densities—in close proximity to the human population, despite sustained elimination efforts, including source reduction, traps that remove gravid mosquitoes, outbreak investigations around clusters of cases, and public health education campaigns [9,10]. The continued presence of *Ae. aegypti* has led to dengue being endemic in Singapore, with substantial impacts on health and the economy [11].

As in many other metropolises, it is commonplace for residents to live and work in different parts of the city. As a result, although vector-borne diseases are geographically clustered, because of the localized presence of infected mosquitoes, humans may act as a conduit facilitating longer distance spread to seed new foci of infection [12–14]. It is therefore vital to understand the contribution of both human mobility and the length of mosquito ranges to arbovirus dissemination.

The Zika outbreak that was first identified in Singapore in August 2016 provided a unique opportunity to illuminate the geographical spread of a nascent arbovirus outbreak across the city-state. Within several weeks of its first identification within and around a construction site in the southeast of the country, the outbreak had spread to multiple secondary loci across many parts of the island [8]. Over the next month, there were approximately 100 confirmed cases a week, of the same order of magnitude as the four serotypes of dengue [15]. Like the dengue viruses, Zika is transmitted primarily by *Ae. aegypti*, and in consequence, there was, and remains, the potential that the ZIKV could become similarly endemic. Autochthonous ZIKV transmission is concerning, because of the risk of severe complications including Guillain–Barré syndrome and microcephaly [16–18], which, although not reported to date in relation with ZIKV infection in Singapore, makes successful control imperative.

As a legally notifiable disease in Singapore, the residential and workplace addresses of confirmed cases of ZIKV disease were collected by the Ministry of Health under the proviso of the Infectious Diseases Act [8]. Despite the ongoing baseline vector control programme that targets *Aedes* mosquitoes in residential areas, the outbreak demonstrates the possibility that human movement may allow the virus to break the ‘cordon’ of vector control and lead to cross-island transmission. In contrast to dengue, which has been endemic in Singapore since at least the 1960s [10,19], the 2016 Zika outbreak spread from one initial locus and across a population that has heretofore not experienced recorded Zika outbreaks; like dengue, it is expected that there be relatively little seasonal forcing on transmission risk [20]. Together, these characteristics make Singapore an ideal model system to understand Zika transmission.

The objective of this study was to assess the contribution of workplaces to the transmission of ZIKV infections during the first wave of ZIKV in Singapore. To this end, we used spatio-temporal models built using Bayesian methods to comprehensively analyse the autochthonous spread of Zika, exploiting the home and work addresses and date of symptom onset of 323 Zika-confirmed cases. To quantify the role of workplaces to the transmission of ZIKV, we explored the space of possible infection trees (i.e. *who-infected-whom-where*) to infer how much infection, and how much transmission, occurs around the home and workplace of cases. This may provide valuable information to guide vector control efforts for future outbreaks both in Singapore and elsewhere.

## 2. Material and methods

### 2.1. Zika case data

The Zika outbreak in Singapore resulted in 323 Zika-confirmed cases between July and September 2016 [8]. As Zika had been made a notifiable disease, attending physicians were required to notify the Ministry of Health for all cases. Under the auspices of the Infectious Diseases Act, the residential and workplace addresses, and date of symptom onset, were recorded for these confirmed cases.

### 2.2. Public transport data

Public transportation is the most common mode of transport of the residents of Singapore [21]. There are three main modes of public transportation in Singapore: the Mass Rapid Transit (MRT), the Light Rail Transit (LRT) and public buses. To pay the public transport fares, most commuters use an EZ-link contactless card: a smart card that is read when entering or leaving the MRT or LRT network or when boarding or alighting a bus. Using data on all users of EZ-link cards over one month from the Land Transport Authority of Singapore, which captures the time and location of commuters' intersecting with the public transport system, we identified individuals who visited within a 1 km square centered on the initial focus, and for each inferred a plausible home address and a plausible work address too using the algorithm described in the electronic supplementary materials. We then discarded those with neither an inferred home nor work address within that 1 km square and summarized the distribution of distances between the addresses of those remaining.

### 2.3. Spatio-temporal model

We developed a spatio-temporal model using the residential and workplace addresses, and date of symptom onset (as a proxy for time of infection), of the 323 Zika-confirmed cases between July and September 2016 to estimate the source of infection for each case using Bayesian data augmentation.

While contact tracing is possible for diseases like severe acute respiratory syndrome (SARS) [22], it is a challenge for vector-borne disease as we are, obviously, unable to contact trace the mosquitoes, and as a result we do not know the source of infection for each case, i.e. the case infecting the mosquito infecting that case. The location where the individual is infected is unobserved and may often be their home or other places they spend significant amounts of time, such as their place of work. In addition, there is very little information available on those individuals who escape infection altogether. Calculating the likelihood of the parameters becomes challenging without knowing the extra information. To address this uncertainty, we developed a Bayesian data augmentation framework which considers the source of infection as augmented data or nuisance parameters [23–25]. In this analysis, the parameter space includes the unknown nature, location and time of infection. Flat prior distributions were assumed for all parameters (details in the electronic supplementary materials).

**Figure 1.**
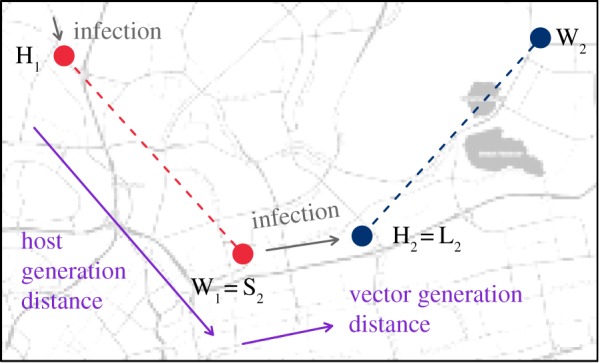
Notation and model of host and vector generation distances. Individual 1 (red dots) resides at H_1_ and works at W_1_. She was infected in the vicinity of her home and she seeds an infection (or a cluster, S_2_) near her workplace; this represents the host generation distance. This cluster then infects individual 2 (blue dots) near his home, H_2_. This typically shorter distance is the vector generation distance. The host generation distance is typically longer than the vector generation distance because humans may act as a conduit to longer distance spread to seed new foci of infection. (Online version in colour.)

Assuming that *I_j_* is the individual who infects individual *j*, we denote *h_ij_* to be the hazard of infection from a potential infecting individual *i* to individual *j*, which is parameterized as follows. The probability that *i* is the individual infecting *j* is:

$$\mathrm{Pr}(I_{j}=i)=\frac{h_{ij}}{\sum_{k:t_{k}<t_{j}}h_{kj}},$$

where the time dependence of *h_ij_* is suppressed for brevity and *t_k_* is the day that individual *k* had the onset of symptoms. The hazard of infection is assumed to be additive across sources and multiplicative in time and space and, hence, the hazard can be written in terms of the following spatial and temporal kernels:

$$\begin{array}{rl} & h_{ij}\propto f_{T}(t_{j}-t_{i})[\;f_{D}∥H_{i},\;H_{j}∥+\;f_{D}∥H_{i},W_{j}∥+\;f_{D}∥W_{i},H_{j}∥\\ & \;+\;f_{D}∥W_{i},W_{j}∥ \end{array}],$$

where $f_{T}(\delta )=1/\delta \sigma \sqrt{2\pi }\exp\{-({\left(\log(\delta )-\mu \right)}^{2})/(2\sigma^{2})\}$ (for *δ* > 0 and 0 otherwise) is a time and *f*_D_(*d*) = *λ*exp(−*λd*) a spatial kernel representing host and mosquito movement (for space) and incubation (for time). It is worth noting that the temporal kernel represents the convolution of extrinsic and intrinsic incubation, and these are not inferred or modelled separately. We assume that the hazard for the time between infections is proportional to the lognormal density with mean *μ* and standard deviation *σ* on the log scale, set to be consistent with the 10–23 days serial interval range for Zika fever [26] as there was insufficient information to directly estimate the temporal kernel from the data. Three fixed temporal density were considered: lognormal(12,3), lognormal(14,3) and lognormal(16,3); in addition a distribution with free parameters, lognormal(*μ*,*σ*), was also considered. We assume that the distances between infections have kernels proportional to the exponential density with rate *λ* estimated from the data. In this model, *t_i_* is time *i* had onset of symptoms, *H_i_* and *W_i_* are coordinates of *i*'s home and workplace addresses, respectively. The individual can be infected at home or work and can infect others near his or her home or workplace. *L_j_* ∈ {*H_j_*, *W_j_*} is the vicinity individual *j* was infected at and $S_{j}\in \{H_{I_{j}},W_{I_{j}}\}$ the vicinity *I_j_* infects *j*. Here, we also defined $∥L_{I_{j}},S_{j}∥$ to be the host generation distance, namely the distance from where *I_j_* was infected to the location closest to where s/he infected *j*, and $∥S_{j},L_{j}\;∥$ to be vector generation distance, namely the distance from the location *I_j_* exposed *j* at to where *j* was infected. The notation and terminology are represented in figure 1.

A custom-designed Markov chain Monte Carlo algorithm [23,24] was developed to allow for rapid mixing through the joint posterior distribution of the parameters, which governs both the spatial and temporal aspects, and the augmented data. A standard Metropolis–Hastings step was implemented to update the parameters [27,28] using a standard Gaussian proposal distribution with bandwidth determined from pilot tests. A Gibbs step was implemented to update the source of infection for case *i*. The source (individual) must precede the new case and is sampled with the proposal distribution with mass:

$$w_{i,j}=\frac{\beta \;f_{T}(t_{i}-t_{j})\;[\;f_{D}∥H_{i},H_{j}∥+\;f_{D}∥H_{i},W_{j}∥+\;f_{D}∥W_{i},H_{j}∥+\;f_{D}∥W_{i},W_{j}∥]}{\sum_{k:t_{k}<t_{j}}\beta \;f_{T}(t_{j}-t_{k})[\;f_{D}∥H_{k},H_{j}∥+\;f_{D}∥H_{k},W_{j}∥+\;f_{D}∥W_{k},H_{j}∥+\;f_{D}∥W_{k},W_{j}∥]}.\;$$

Here, *f*_T_ and *f*_D_ are taken to be proportional to lognormal and exponential distributions, respectively, while *β* is a constant of proportionality that cancels in the Gibbs step.

### 2.4. Estimation of infection tree—*who-infected-whom-where*

From the spatio-temporal model, we inferred (i) *L_j_* the location where individual *j* was infected and (ii) *S_j_* the potential source which was seeded by individual *I_j_* (i.e. *I_j_* is the ‘human donor’ who infected the *Aedes* mosquitoes in the vicinity of *j* which then went on to infect *j*). With this, we estimated an infection tree (i.e. *who-infected-whom-where*).

The majority of the ZIKV infections were not severe resulting in ambulant infected individuals who may act as the ‘vector’ infecting distant parts of the country. Given the infection tree, we identify several individuals who may be responsible for spreading Zika to immune-naive regions of Singapore. These individuals, or super-dispersers, were the most probable donors who resulted in the infection of other individuals at a relatively far distance (beyond an arbitrary threshold *θ* = 15 km, corresponding roughly to the 95th percentile of the cumulative distribution function (CDF)) from where they were infected. Again, assuming *I_j_* to be the human donor responsible for infecting individual *j*, *I_j_* was responsible for seeding the source location of infection of individual *j*, *S_j_*. The distance between *S_j_* and the location at which *j* was infected at, *L_j_*, is given by $∥S_{j},L_{j}∥$ and the distance between the location where *I_j_* was infected at, $L_{I_{j}}$, and the location at which *j* was infected, *L_j_*, is $∥L_{I_{j}},L_{j}∥$. Individual *I_j_* is identified as a super-disperser if $∥\;L_{I_{j}},L_{j}∥-∥S_{j},L_{j}∥>\theta$. For example, an individual infected at work could infect mosquito(es) in his residential area (formerly without an outbreak and distant from other potential sources) which after several days results in the infection in other individuals around that area. This individual would be flagged as a super-disperser if his home and work were sufficiently distant.

Three variants of the basic model were developed—*home-alone*, *work-alone* and *home-and-work*. The first two models consider only home or work address, respectively, as a source of infection while the third model considers both home and workplace as potential sources of infection. Using the deviance information criterion (DIC) [23], we chose the model variant that best fits the data. To display uncertainty in the source of infection, we derive spatial posterior density plots of the location of the source of infection for each case, separately, using bivariate kernel density estimation on the posterior samples of sources of infection. These posterior density maps aggregate cases located close to each other in space, thereby providing a fairer depiction of the uncertainty in infection locations than an individual-based metric. The posterior distribution of the CDF for the distance between where the inferred donor was inferred to have infected the recipient and where the recipient was inferred to have been infected was derived by calculating the empirical CDF for each iteration of the algorithm and aggregating over iterations to obtain the posterior mean and an equal-tailed 95% credible interval. This was separately calculated for each temporal kernel, and for the *home-alone* and *work-alone* variants of the model.

For each model, we ran the algorithm for 100 000 iterations with every 10th iteration retained following a burn-in period of 5000 iterations. Convergence was assessed visually with trace plots. The average computational time of a run with 100 000 iterations is approximately 3 h on a desktop computer.

We created a simulated outbreak on a spatial domain of radius 20 km, i.e. of approximately the surface area of Singapore's main island, Pulau Ujong, and we also built an individual-based simulation model in which each member of the resident population is represented by a line-listing, with home addresses assigned to residential addresses extracted from a geographical information system (GIS) in-line with the number of residents by age and gender in each subzone, an administrative division of Singapore (described in the electronic supplementary materials).

The analyses and model building were performed in R [29] and the geographical visualizations were done in R [29] and QGIS [30]. Ethical approval was obtained from the National University of Singapore Institutional Review Board.

## 3. Results

**Figure 2.**
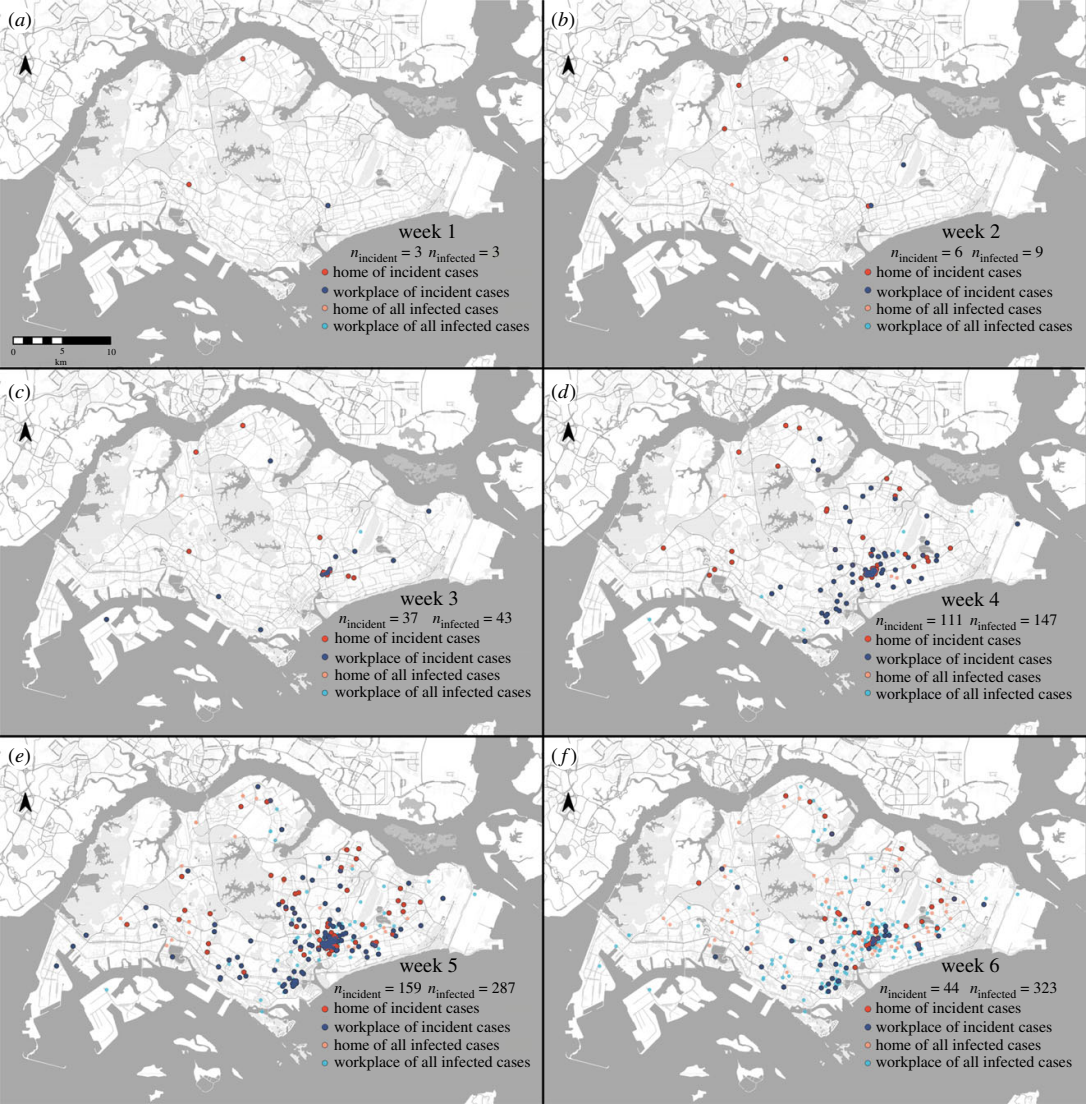
Spatial-temporal transmission of ZIKV infections in the first six weeks. Home (red and orange dots) and workplace addresses (light and dark blue dots) of Zika-confirmed cases are geographically represented across Singapore. Recent weekly incident infections are enumerated for each week and emphasized by the darker shades of red and blue. The cumulative number of infections each week is also presented. (Online version in colour.)

**Figure 3.**
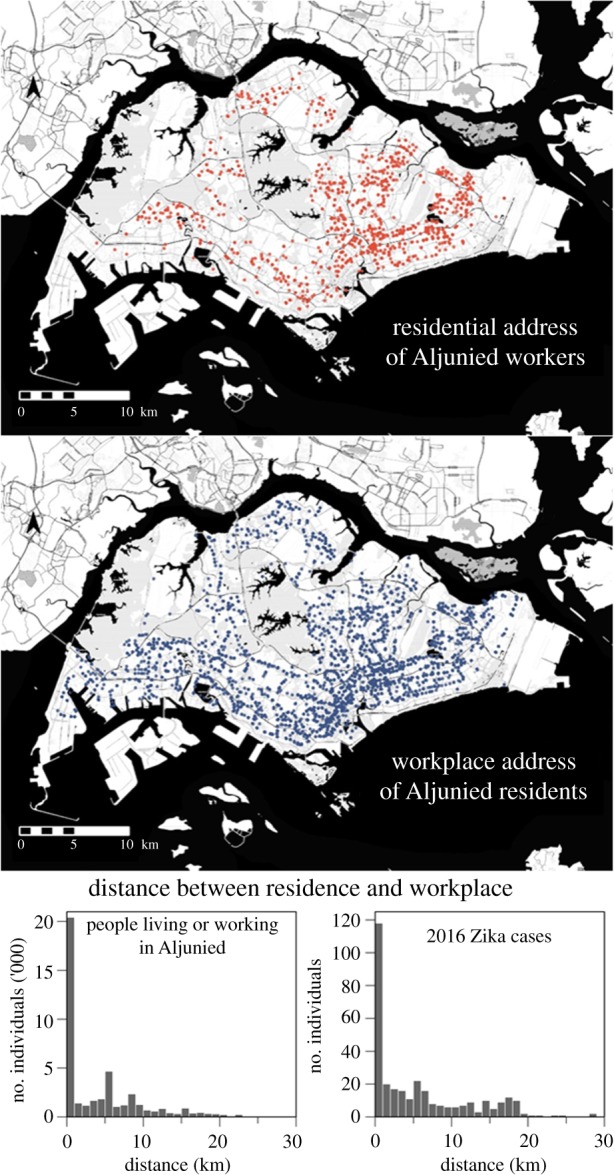
Geographical distribution of residential and workplace addresses of individuals working and residing 1 km within the epicentre of the 2016 Zika outbreak. Euclidean distances between residence and workplaces, *l*^2^-norm, were calculated for population (top right) and Zika cases (bottom right) residing and working in near the epicentre.

The spatio-temporal evolution of the outbreak depicted in figure 2 shows how the initial focus within and around a construction site in the Aljunied neighbourhood (in the Southeast of the country and on the periphery of the downtown core of Singapore) rapidly proliferated across the island over four weeks [8]. This rapid dissemination coincided with the relatively large distances between the homes and workplaces of cases, with 25.4% of cases working at least 10 km from their home (on an island that spans 50 km at its greatest extent); this mirrors the relatively long distances travelled by commuters working or living in the Aljunied area (figure 3), of whom 14.0% travel more than 10 km to get to and from work. A permutation test of median differences showed some evidence that cases on average travelled further than the reference group of people living or working in Aljunied (*p* = 0.01). Despite this, the distributions are qualitatively similar, which indicates that even if the initial focus had not been at a construction site—leading to a substantial number of cases being construction workers mostly living in foreign worker dormitories on the other side of the island—there would still have been considerable risk of secondary foci being seeded far from the initial outbreak.

**Figure 4.**
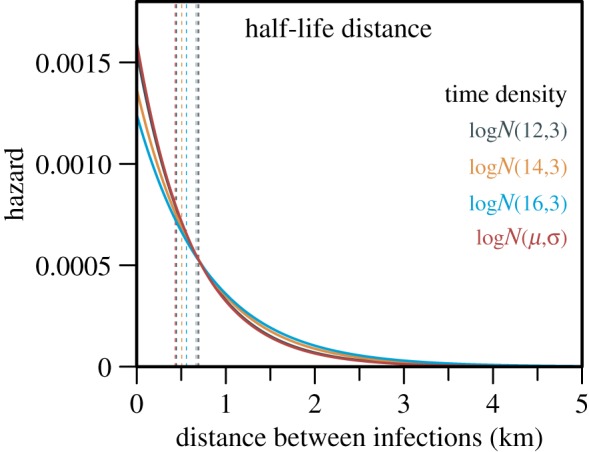
Posterior mean infectious hazard as a function of distance, in kilometres, between Zika infections. The solid lines indicate the posterior mean hazards and the posterior mean half-life distances are represented by dashed lines. A version in which the temporal effect parameters were estimated directly is coloured red; other colours are for sensitivity analyses in which time parameters were fixed at arbitrary values within the feasible serial interval range of Zika fever. (Online version in colour.)

**Figure 5.**
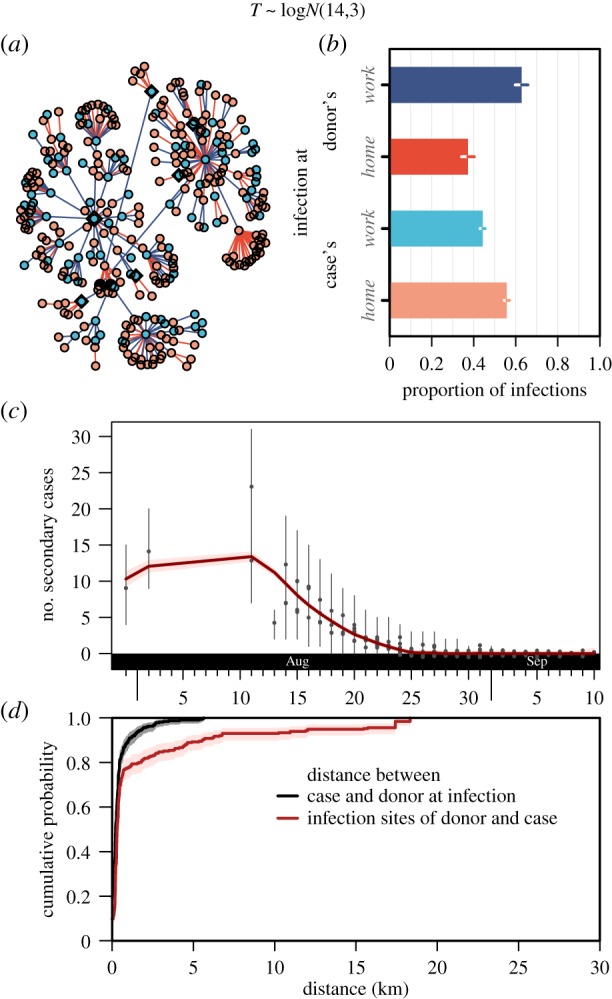
Estimated *who-infected-whom-where* in the 2016 Zika outbreak in Singapore. (*a*) The directed infection tree was estimated from the epidemiological data. The cases could be infected at home (blue dots) and work (orange dots) by the inferred donor. The donor can infect their secondary cases near home (blue line) or work (red lines). (*b*) The bar chart of proportions and 95% credible interval show that both home and workplaces were essential to understand the Zika outbreak in Singapore. (*c*) The number of secondary cases (grey dots are posterior median, and the grey lines depict the 95% credible interval) determined from the estimated infection tree was calculated over time. These dots are plotted on the day of symptom onset of the infector and the multiple dots on the same day implies multiple infectors (jittered for visual clarity). The loess-smoothed mean number of secondary cases are plotted in red (the 95% confidence interval shaded in pink). (*d*) Euclidean distance, *l*^2^-norm, between case and donors at infection were compared against the infection sites of donors and cases. (Online version in colour.)

The posterior mean half-life distances between infections—which represents a combination of human movement in the vicinity of their home or workplace and of mosquito movement over the incubation period—of the different models fitted ranged from 430 to 560 m (figure 4). Some cases were infected relatively far from where extant cases were (figure 5*d*). Thus the inferred infection tree suggests that several ambulant cases acted like long-distance vectors, or super-dispersers, as they exposed distant and as-yet unaffected neighbourhoods to ZIKV transmission.

A substantial proportion (64.2%) of ZIKV infections occurred at workplaces (of the donor or recipient case, or both), as seen from the estimated infection tree (figure 5*a,b*), signifying the importance of not limiting control efforts to the vicinity of residences of cases. The number of secondary cases that resulted from the initial few cases was large but as the outbreak progressed, the data suggested that the transmissibility had reduced (figure 5*c*). When we excluded infections linked to the construction site at the initial epicentre, 51.0% of infections occurred at either the donor or recipient's workplace.

Although the generation time distribution could not be reliably estimated from the spatio-temporal data due to the multiple overlapping transmission events, results were robust to the generation time distributions assumed (the electronic supplementary materials present alternative results). When we considered a variant of the model in which only one address type was used, there was strong support in favour of the model which considered both *home-and-work* addresses compared to the *home-alone* or *work-alone* models (DIC tabulated in table 1), thus indicating the role both locations play and the importance of considering both location types for vector control efforts. All variants of the model support the finding that the reproduction number reduced from 5 or more in the early period of the epidemic to below unity for cases with onset after mid- to late-August, i.e. that secondary cases fell a few weeks thereafter. Although the cause of the reduction cannot be determined by the model structure, this coincided with the beginning of vector control targeting the outbreak site around the end of August 2016. Although the models in which the parameters of the time kernel were fixed led to similar numbers of secondary cases, that of the model in which the time kernel's parameters were left as free parameters exhibited a profile that was qualitatively different.

**Table 1.** Deviance information criterion (DIC) of the models.

model		deviance information criterion (DIC)
*T* ∼ log*N*(12,3)	*home-and-work*	6002
	*home-alone*	6214
	*work-alone*	6213
*T* ∼ log*N*(14,3)	*home-and-work*	6136
	*home-alone*	6414
	*work-alone*	6413
*T* ∼ log*N*(16,3)	*home-and-work*	6286
	*home-alone*	6577
	*work-alone*	6577
*T* ∼ log*N*(*μ*,*σ*)	*home-and-work*	5158

The posterior density of the estimated location of infection for each case for the models with various temporal kernels is presented in the electronic supplementary materials. These demonstrate, as with the number of secondary cases against time and general network topology, that the source of infection is relatively robust to the specification of the temporal kernel within the range of kernels considered. These spatial figures also demonstrate that for most cases, the source of infection can be inferred to a relatively limited set of potential foci. The fraction of infections attributable to transmission events at each combination of the donor's and the recipient's home and work was consistent across the parameters considered for the temporal kernel (electronic supplementary materials). In the early phase of the outbreak, most infections were estimated to have occurred near the case's workplace, with approximately equal numbers of infections at home and at work in the second half of the outbreak (electronic supplementary materials). These results were robust to the temporal kernel considered. The posterior CDF between infections is presented in the electronic supplementary materials for different temporal kernels and the model variants in which transmission is allowed to occur only near homes or only near workplaces. This shows that the inferences on the spatial signature are robust to the time kernels and that substantially more longer distance infection events are required to explain the outbreak if it is assumed that only homes or only workplaces are responsible for transmission.

Despite knowing approximately 20% of the actual infections in the simulation exercise, the inference performed well to infer the location of infection of the infected cases (electronic supplementary materials).

## 4. Discussion

Shoe-leather epidemiology to trace the route of transmission between cases of a mosquito-borne disease is challenging, as there may be no discernible links between cases separated by one human–mosquito cycle of transmission. Statistical methods first developed during the SARS epidemic [31] are capable of inferring transmission trees but need to account for geographical structure for vector-borne diseases, in which the vector has a considerably shorter range than does the host. The method presented in this paper is an extension of the transmission tree reconstruction methods as described by Wallinga & Teunis [31] that extends their temporal kernel, represented by *f*_T_(*δ*) in our paper, to account for space, through a spatial kernel *f*_D_(*d*), and by considering two main location types for each case, namely their home and workplace. Although the locations of cases by themselves make a convincing case for the role of humans as a ‘vector’ transmitting the virus to naive regions of the city, this extension to the Wallinga and Teunis method allows the degree of such long-distance spread, and the importance of the workplace in transmission, to possibly be quantified. Although applied to a specific outbreak, the method could potentially be useful in other settings, in particular, when the amount of genetic variability is insufficient to allow transmission trees to be inferred using phylogenetic methods.

Because ZIKV infections, in common with other arboviruses such as dengue, are predominantly clinically mild [8], infected hosts may not be isolated from the vector, and through their continued mobility may disseminate infection far from their home, or from the location they were infected. The potentially high fraction of such ambulant, subclinical infections may lead to delays in the identification of new foci of infection. Moreover, in an urban outbreak like this, the distance from home to work may be large relative to the scale of transmission and, as a result, ambulant infected persons may act as the ‘vector’ infecting distant parts of the country [12–14], further complicating control. In this analysis, we identified some individuals who could be super-dispersers—or long-distance human vectors—for ZIKV infection, and a key priority in future outbreaks should be to identify such individuals early and ideally isolate or protect them from being bitten by mosquitoes. Such individuals are not unusual, as seen in the long tail for commuting distances for people living or working near the initial focus of the outbreak.

Conventionally, the residential address is the usual proxy for the location of infection in spatial analyses [32], but this study identified workplaces as a substantial source location for transmission. Across all the models presented, the proportion of infections occurring around workplaces was substantial. The results represented in figure 5*b*,*c* provide some empirical evidence that the national vector control programme should consider both types of location (home and workplaces) when defining clusters for interventions. As a substantial proportion of infection occurs at workplaces, control measures targeted at residential regions of the city-state may be ineffective to contain the spread of an arbovirus outbreak. In the past, vector control measures concentrated on residential areas, although the national vector control programme now considers both types of location in defining clusters for interventions. Substantial amounts of infection occurring at workplaces will make a control programme that focuses on the home less effective, and as a result, the method developed in this study may potentially better inform vector control measures. Given the endemicity of dengue in Singapore, it would be of some interest to investigate the degree to which dengue infections occur away from the home, especially in the light of previous research which showed infection risk varying over a weekly cycle [33].

The methods described in this study met with several challenges. Neither the exact location nor the time of infection of the cases was known, and to overcome this, we used the residential and workplace addresses and date of symptom onset as proxies of place and time of infection. Transmission in the neighbourhood of the home or workplace is implicitly captured through the dispersal kernel, but it is a limitation that other locations frequented by cases (such as eating houses or places of worship [34]) could not be recorded. Owing to the non-negligible asymptomatic rate of ZIKV infections, we may have incomplete information on potentially infectious individuals despite the good surveillance system in Singapore. A previous analysis of this outbreak sought to infer the phylogeny of a fraction of the cases, but the low mutation rate led to a lack of variation in the genetic data [8], and as such, the epidemiological data provide greater information on the transmission dynamics. Over a longer time period, more mutations would be expected, and a marriage of field and molecular epidemiological information may be warranted for a longer outbreak [35–37]. Cross-immunological effects have been demonstrated [38,39] between dengue and zika, and it is not clear what effects this might have had on transmission potential in Singapore, given the high prevalence of dengue. In initial model building, we sought to specify non-informative priors for the parameters of the temporal kernel, but there was insufficient information to allow these to be estimated together with the other estimands. We therefore set biologically plausible values of these parameters and assessed the sensitivity of the results to the values chosen. The main findings—in particular, the role of workplaces in transmission and the inferred locations of infection—were robust to this choice.

This study is both important epidemiologically—indicating the importance of considering both residential and workplace addresses to inform the vector control measures for diseases like Zika and dengue—and involved new methodological development to tease out the multiple spatial patterns driving this urban outbreak. Incorporating spatial data in the model allows us to study the geographical-related exposures for these vector-borne diseases. Singapore's outbreak appears qualitatively different from those in less urbanized environments [40–42], in that infected *Aedes* mosquitoes played a less significant role in the long-distance transmission of Zika than did ambulant cases, which may have implications for control of subsequent arbovirus outbreaks in urban settings like Singapore.

## Supplementary Material

Supplementary Materials

## Supplementary Material

Additional Supplementary Figures

## Data accessibility

This article has no additional data.
