# Supplementary material for: Inferring who-infected-whom-where in the 2016 Zika outbreak in Singapore—a spatio-temporal model
Source: J R Soc Interface. 2019 Jun 19;16(155):20180604. doi: 10.1098/rsif.2018.0604 (PMC6597776; doi:10.1098/rsif.2018.0604)
Supplement: Supplementary Materials [file rsif20180604supp1.pdf]

Supplementary Materials

Inferring *who-infected-whom-where* in the 2016 Zika outbreak in Singapore: a spatial-temporal model

Kiesha Prem, Max SY Lau, Clarence Tam, Marc ZJ Ho, Lee-Ching Ng, Alex R Cook

Table of Contents

Background of Singapore and the population at risk of Zika 2
Methods 4
Inference 4
Notation 4
Sensitivity analyses: *Home-alone* and *work-alone* models 4
Super-dispersers 5
Genetic data 5
Markov Chain Monte Carlo Algorithm 5
Simulation study 7
Results 8
Time-free model 10
Home-alone model 11
Work-alone model 12
Simulation study 16
References 19

Background of Singapore and the population at risk of Zika

Singapore is a highly urbanised city-state in Southeast Asia with a tropical climate, around 100km from the equator. It has a land area of ~720 square kilometres and its mainland spans 50km from east to west and 27km from north to south [1]. Like many other highly urbanised cities, most residents live in high-rise apartment flats with many neighbours. Despite intensive eradication efforts, the *Aedes* mosquitoes (*Ae. aegypti* and *Ae. albopictus*) remain at large in these living environments [2,3]. The close proximity of these mosquitoes and humans allow for more interactions between them which may trigger arbovirus outbreaks such as dengue [4–8], Chikungunya [5,9,10], and Zika [11]. The mild clinical severity of most Zika infections may allow an ambulant infected individual to seed new foci of infection [12–14], e.g. around their workplace or school. This is particularly salient as the small size of the country means the distance travelled from home to work in Singapore constitutes a larger fraction of the country's size than in most other countries at risk of arboviruses. As a result, the transmission patterns of vector-borne disease may be more comparable to other high-urban cities than to other countries.

The majority of the population uses public transport to commute to work or school. From the Singapore census in 2010 [15], 87% of the population relies on some means of transport to get to work or school, of whom about 57% regularly use the public transport to get to school/work. Most public transport is paid for using a contactless payment card (commonly called an EZ-link card) that allows capture of transport usage data, including the times and locations of trips. All trips made over a one-month period (August 2013) were provided as anonymous data by the Land Transport Authority of Singapore. From these, we extracted out all records in which a trip started or ended within a 1km square cantered on the initial outbreak focus. For each card (which we treated as equivalent to a unique individual: although cards are transferable, commonly individuals retain a single card for years) in this reduced dataset, we inferred primary and secondary addresses based on the following algorithm, which we labelled their home and work location, respectively.

We used each EZ-link card as a proxy for an individual, ignoring the possibility of a card being transferred from one individual to another or multiple cards being used by the same individual within the one-month period of the dataset. The location at the start and end of each journey was determined by geo-referencing each bus stop and train station. When an individual neglected to 'tap out' at the end of a journey, as can happen on buses, there is no end point recorded for the journey, and for such journeys, only the start point was used. We extracted the card number for any trip that started or ended within a 1km square centred on the initial focus of the outbreak in Aljunied. For each such card, we extracted all starting and ending locations throughout the one-month period of the dataset provided by the Land Transport Authority.

To impute a home location for each card, we identified the first and last trips made per day. We then applied a two-dimensional Gaussian kernel with arbitrary bandwidth of 0.001 degrees of latitude or longitude around each starting or ending point, primarily to accommodate individuals who board and alight from buses on opposite sides of the street on the way to and from work. We then identified the point among these with the highest summed kernel to be the imputed home location. Bus stops are typically located quite close to one another and to

high density urban neighbourhoods where most Singaporeans live, so the error between the actual home and imputed home is likely to be of the order of a few hundred meters.

To impute a work (or school) location, we followed a similar approach, but using the last trip made before noon and the first trip made after noon. We then counted the number of days in which the imputed work address was visited and excluded those with fewer than 15 visits over the one month period. Those removed were suspected of having multiple work sites or of the trips not being to a workplace.

We then removed any individual who did not have an imputed work or home location within the 1km square cantered on the epicentre. All remaining location-pairs were then used as a proxy for the commuting patterns to and from the initial outbreak site.

Methods

In this study, we developed a spatio-temporal model using the residential and workplace addresses and time of infection of the 323 Zika confirmed cases between July and September 2016 to estimate the source of infection for each case using Bayesian data augmentation.

Inference

The source of infection for each case is unknown, as we are unable to do contact tracing to identify the transmission chain due to the role of the vector. As a result, the location where the individual is infected is unobserved. In addition, all non-cases are also unknown. Consequently, these challenges make defining the likelihood function of the parameters difficult. To address these challenges, we developed a Bayesian data augmentation framework which considers the source of infection as augmented data or nuisance parameters [16]. In this analysis, the parameter space includes the unknown source of infection, following the approach of Gibson and colleagues [17]. The parameters in the model are defined in the table below.

Notation

Symbols	Description
t_i	Time of symptom onset for i
h_{ij}	Hazard of infection of an infected individual j from its source seeded by individual i
H_j and W_j	Coordinates of j's home and workplace addresses respectively
$L_j \in \{H_j, W_j\}$	Place/vicinity individual j was infected
I_j	Individual who 'infects' individual j (through infecting the vector that infects j)
$S_j \in \{H_{I_j}, W_{I_j}\}$	Place/vicinity I_j infects j
$\lVert L_{I_j}, S_j \rVert$	Host generation (Euclidean) distance: between the location the donor was infected and the recipient was infected
$\lVert S_j, L_j \rVert$	Vector generation (Euclidean) distance, from the location the donor seeded infection to the location the recipient was infected
θ	Threshold distance to determine a super-dispersal event

Sensitivity analyses: *Home-alone* and *work-alone* models

In the sensitivity analyses to assess the importance of including workplaces in the model, we considered two spatial-temporal models where an individual can only be infected at home or can only be infected at work, which we called the *home-alone* and *work-alone* models.

The hazard of infection under this model is $h_{ij} \propto f_{\mathrm{T}}(t_j - t_i)[f_{\mathrm{D}}\lVert H_i, H_j \rVert]$ instead of $h_{ij} \propto f_{\mathrm{T}}(t_j - t_i)[f_{\mathrm{D}}\lVert H_i, H_j \rVert + f_{\mathrm{D}}\lVert H_i, W_j \rVert + f_{\mathrm{D}}\lVert W_i, H_j \rVert + f_{\mathrm{D}}\lVert W_i, W_j \rVert]$ in the *home-and-work* model. The temporal and spatial kernels are as before, namely $f_{\mathrm{T}}(\delta) = \frac{1}{\delta\sigma\sqrt{2\pi}} \exp\left\{-\frac{(\log(\delta)-\mu)^2}{2\sigma^2}\right\}$ and $f_{\mathrm{D}}(d) = \lambda \exp(-\lambda d)$ for non-negative distances and positive times, respectively. Similarly, for the *work-alone* model, the hazard of infection is $h_{ij} \propto f_{\mathrm{T}}(t_j - t_i)[f_{\mathrm{D}}\lVert W_i, W_j \rVert]$.

In initial analyses, flat prior distributions were assumed for all parameters, as tabulated below. Parameters were given independent, improper prior distributions.

Parameter	Prior distribution
Temporal kernel centrality	$\mu \sim U(-\infty, \infty)$
Temporal kernel spread	$\sigma \sim U(0, \infty)$
Spatial kernel distance	$\lambda \sim U(0, \infty)$

As there was insufficient information to estimate the temporal kernel from the data directly, we then fixed the temporal kernel parameters to values consistent with the range estimated from Guatemala and Martinique [18]. Specifically, the values used are tabulated below.

Scenario	Mean	Standard deviation
Main analysis	$\mu = 14$	$\sigma = 3$
Alternative 1	$\mu = 12$	$\sigma = 3$
Alternative 2	$\mu = 16$	$\sigma = 3$

The results from the three parameterisations were separately determined and presented.

Super-dispersers

We identified post hoc individuals who were inferred to have seeded infection at distance from where they themselves were infected, whom we labelled super-dispersers. An example of super-dispersion would be when an individual was infected at their place of work, and seeded infection around their home, if the home was distant from the workplace. We arbitrarily set the threshold determining super-dispersion to be 15 km, following trial-and-error: this corresponds roughly to the 95th percentile of the distribution. We then visualised super-dispersers differently on the inferred infection tree to represent their impact on transmission.

Genetic data

Very little genetic variation was found in a previous analysis of a subset of cases during the outbreak [11]. As a result, the epidemiological data was considered better able to characterise the transmission tree than the phylogeny.

Markov Chain Monte Carlo Algorithm

A customised Markov Chain Monte Carlo algorithm was developed to allow for rapid mixing of the joint posterior distribution of the parameters governing both the spatial and temporal kernels and of the augmented data. This cycled between updates of the parameters, using a Metropolis-Hastings step, and of the sources of infection, using a Gibbs step.

Updates of parameters. A standard Metropolis Hastings step was implemented to update the parameters μ, σ, and λ [19,20] using a standard Gaussian proposal distribution with bandwidth determined from pilot tests. In the variant where μ and σ were fixed, no such proposals were made to them.

Update of the source of infection of case i. A Gibbs step was implemented to updates the source of infection for case i and the location that case i was infected at. The source (individual), which must precede the new case, was sampled from the proposal distribution with probability masses given by:

$$w_{i,j} = \frac{\beta\, f_T(t_i - t_j)\,[f_D\|H_i, H_j\| + f_D\|H_i, W_j\| + f_D\|W_i, H_j\| + f_D\|W_i, W_j\|]}{\sum_{k:\, t_k < t_j} \beta\, f_T(t_j - t_k)\,[f_D\|H_k, H_j\| + f_D\|H_k, W_j\| + f_D\|W_k, H_j\| + f_D\|W_k, W_j\|]}.$$

At each iteration, the source for each case was updated.

Implementation. The algorithm is run for 100,000 iterations with a burn in of 5,000, storing 1 out of 10 iterations. Convergence was assessed visually with trace plots, an example of which is presented in **Supplementary Figure 1**. The average computational time of a run with 100,000 iterations is approximately three hours on a desktop. The analyses and model building was performed in R [21] and the map visualisation was done in R and QGIS [22].

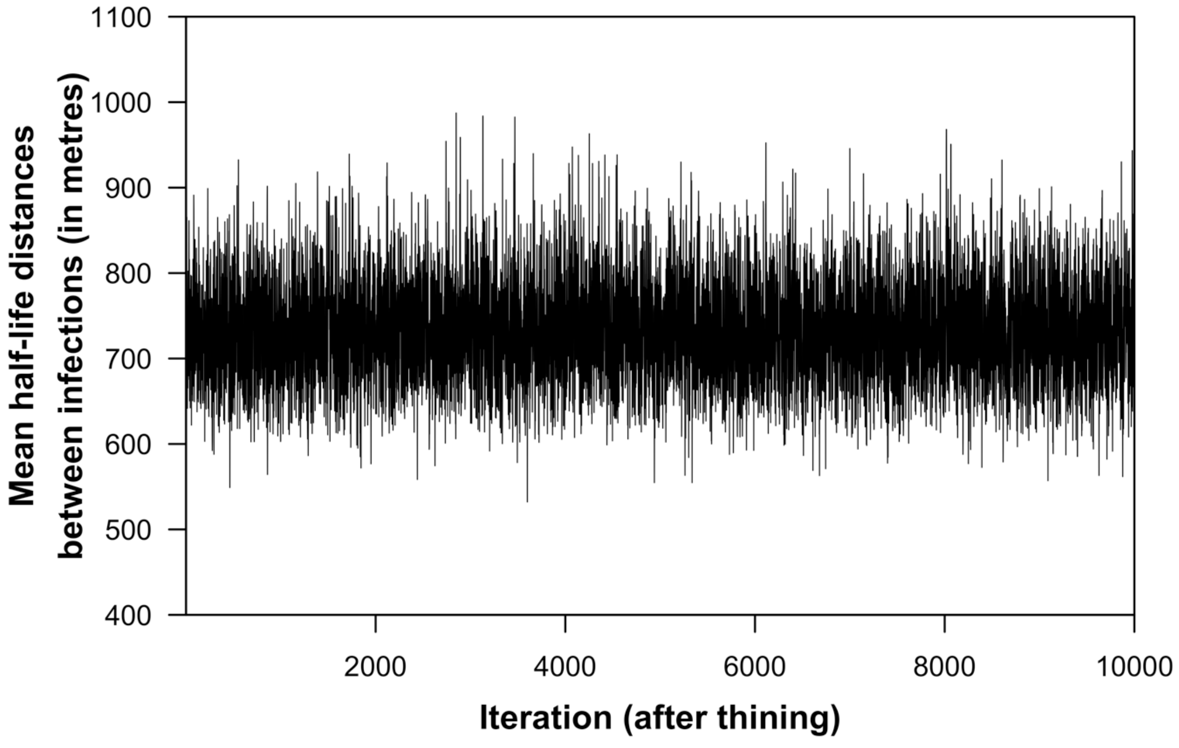

Supplementary Figure 1: Trace plot of the key parameter governing the spatial kernel distance. The algorithm ran for 100,000 iterations and 1 out of 10 iterations was stored and plotted above.

Simulation study

Simple simulation model. We created a simulated outbreak on a spatial domain of radius 20km, i.e. of approximately the surface area of Singapore's main island, Pulau Ujong. The model developed in this paper is not a good data-generating model, because some signals in the data are inferred but not explicitly modelled, such as the change in transmission rates over time. The simulated outbreak was therefore generated with an extension to this model that imposed structure akin to that observed in the data, as described below.

On infection of a case, a secondary location is generated for the case by generating an angle uniformly on $(0, \tau)$ (τ being the transcendental circular constant, ~ 6.28) and a distance from an exponential distribution with mean 6km, repeatedly generating both quantities should the secondary address fall beyond the spatial domain. These two locations were not assigned specific types but may be taken to be the home and work address.

Each newly infected case i was allowed to seed a new cluster at each of the two locations independently with probability $\pi(t_i)$, where t_i is the time of infection of individual i. We set $\pi(t) = 0.25$ for $t < 30$, $\pi(t) = 0.03$ for $t > 60$ and assume a linear decline between these times, i.e. loosely modelled on the time course of the Singapore outbreak. On seeding a cluster, a Poisson distributed number of secondary cases are generated with mean 7; these are distributed spatially using a $U(0, \tau)$ distribution for the angle and Exp(0.45^{-1}) distribution for the distance. Each case in the simulated outbreak is assumed to be symptomatic with probability 0.2. Those that develop symptoms develop symptoms after a log-normally distributed interval of time, with mean 14d and standard deviation 3d. The simulation is stopped after 120d or when no more clusters are awaiting generation.

Extended simulation model. We also built an individual-based simulation model in which each member of the resident population is represented by a line-listing, with home addresses assigned to residential addresses extracted from a geographic information system in-line with the number of residents by age and gender in each subzone, an administrative division of Singapore. Employment status (in work, in schooling, or neither) was also assigned to be consistent with data from the latest census of population in 2010. A work place or school was assigned to each individual based on distance from home, reported commuting distance (from the census), a list of all schools under the purview of the Ministry of Education of Singapore, and a list of all workplaces by sector obtained from a comprehensive business directory. In this extended simulation study, we hope to demonstrate the robustness of the method to underdiagnosis of Zika in Singapore by generating an outbreak over a realistic arena that properly captures the residential and work place structure of Singapore.

Infection was seeded with a single case seeding a residential cluster. At the beginning of the epidemic, each new case seeded a residential cluster with probability 0.4 and additionally seeded a work-place cluster with probability 0.3. These probabilities were quenched at a rate of 0.02 per day. On seeding a cluster, all individuals living nearby were infected with probability $0.00075 \exp -d_{ij}/200$ where d_{ij} is the distance from the donor i to the potential recipient j in metres. Similarly, all individuals working nearby were infected with probability $0.0002 \exp -d_{ij}/200$. The simulation was continued until all individuals had had a chance to seed clusters, for one year, or until 10 000 cases were infected, whichever came first. We then randomly assigned 20% of the infections to be symptomatic; asymptomatic infections were retained for comparisons.

Results

Several models—fixed temporal density, temporal density with free parameters lognormal(μ,σ)—were considered and the model outputs presented below. We also present the findings from of sensitivity analyses, i.e, the *home-alone* and the *work-alone* models, and a comparison of the results under different models and kernels to assess the robustness of findings.

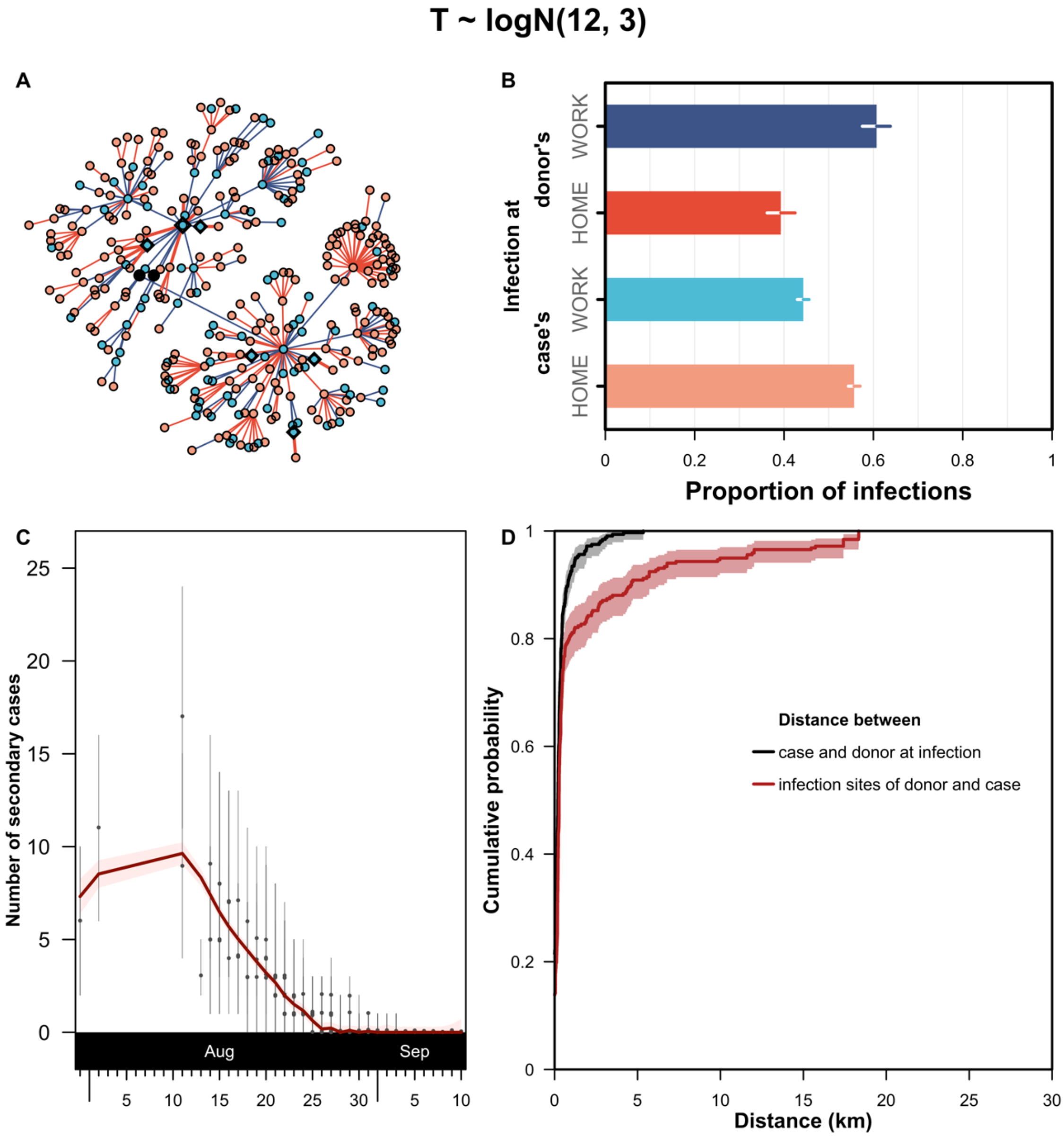

Supplementary Figure 2: Estimated *who-infected-whom-where* in the 2016 Zika outbreak in Singapore for model with fixed temporal density lognormal(12,3). (A) The directed infection tree was estimated from the epidemiological data. The cases could be infected at home (blue dots) and work (orange dots) by the inferred donor. The donor can infect their secondary cases near home (blue line) or work (red lines). (B) The bar chart of proportions and 95% credible interval show that both home and workplaces were essential to understand the Zika outbreak in Singapore. (C) The number of secondary cases (grey dots are posterior median, and the grey lines depicts the 95% credible interval) determined from the estimated infection tree were calculated over time. These dots are plotted on the

day of symptom onset of the infector and the multiple dots on the same day implies multiple infectors (jittered for visual clarity). The loess-smoothed mean number of secondary cases are plotted is red (the 95% confidence interval shaded in pink). (D) Euclidean distance, l^2-norm, between case and donors at infection were compared against the infection sites of donors and cases.

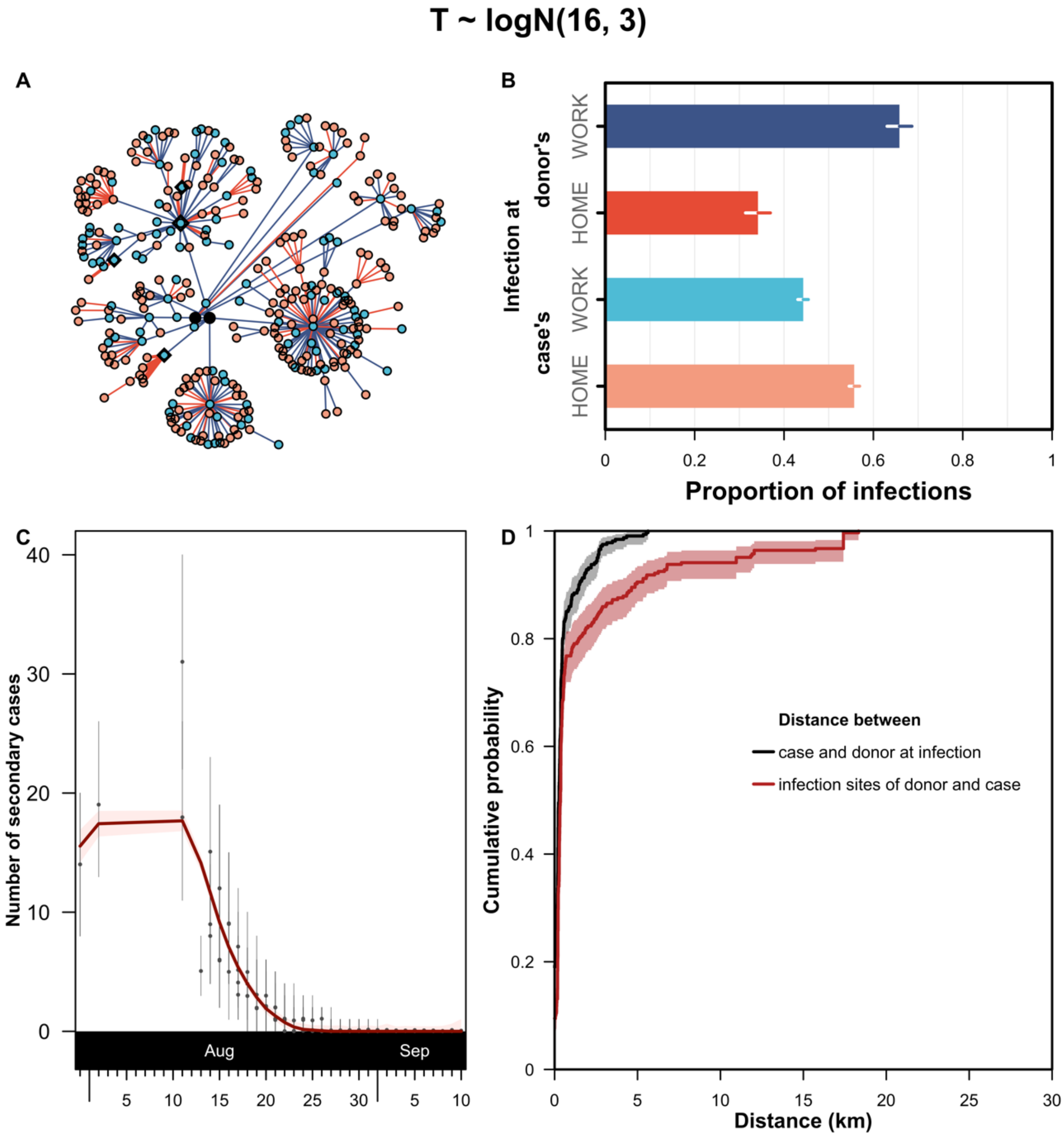

Supplementary Figure 3: Estimated *who-infected-whom-where* in the 2016 Zika outbreak in Singapore for model with fixed temporal density lognormal(16,3). (A) The directed infection tree was estimated from the epidemiological data. The cases could be infected at home (blue dots) and work (orange dots) by the inferred donor. The donor can infect their secondary cases near home (blue line) or work (red lines). (B) The bar chart of proportions and 95% credible interval show that both home and workplaces were essential to understand the Zika outbreak in Singapore. (C) The number of secondary cases (grey dots are posterior median, and the grey lines depicts the 95% credible interval) determined from the estimated infection tree were calculated over time. These dots are plotted on the day of symptom onset of the infector and the multiple dots on the same day implies multiple

infectors (jittered for visual clarity). The loess-smoothed mean number of secondary cases are plotted is red (the 95% confidence interval shaded in pink). (D) Euclidean distance, l^2-norm, between case and donors at infection were compared against the infection sites of donors and cases.

Time-free model

$$T \sim \log N(\mu, \sigma)$$

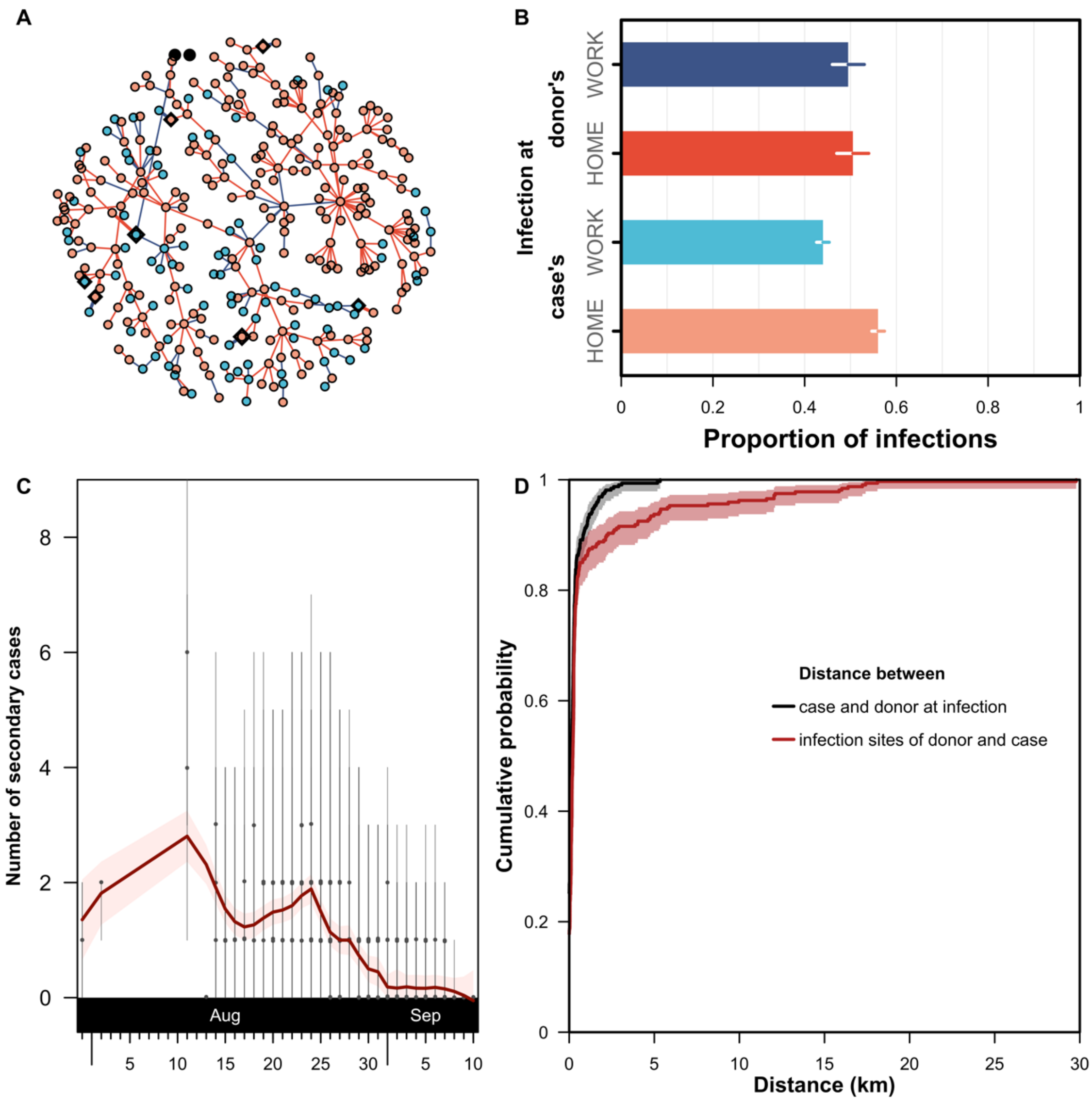

Supplementary Figure 4: Estimated *who-infected-whom-where* in the 2016 Zika outbreak in Singapore for model with temporal density governed by free parameters. (A) The directed infection tree was estimated from the epidemiological data. The cases could be infected at home (blue dots) and work (orange dots) by the inferred donor. The donor can infect their secondary cases near home (blue line) or work (red lines). (B) The bar chart of proportions and 95% credible interval show that both home and workplaces were essential to understand the Zika outbreak in Singapore. (C) The number of secondary cases (grey dots are posterior median, and the grey lines depicts the 95% credible interval) determined from the estimated infection tree were calculated over time. These dots are plotted on the day of symptom onset of the infector and the multiple dots on the same day implies multiple infectors. The loess-smoothed mean number of secondary cases are

plotted is red (the 95% confidence interval shaded in pink). (D) Euclidean distance, l^2-norm, between case and donors at infection were compared against the infection sites of donors and cases.

Home-alone **model**

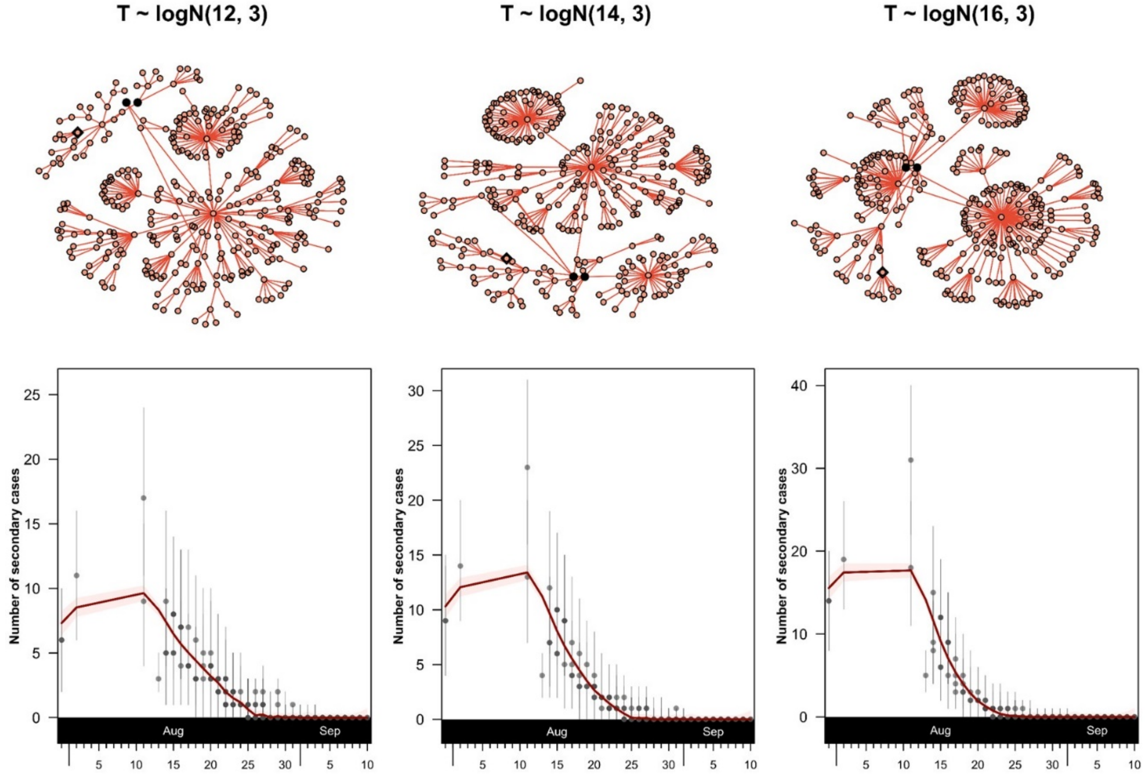

Supplementary Figure 5: *Home-alone* model.

Work-alone model

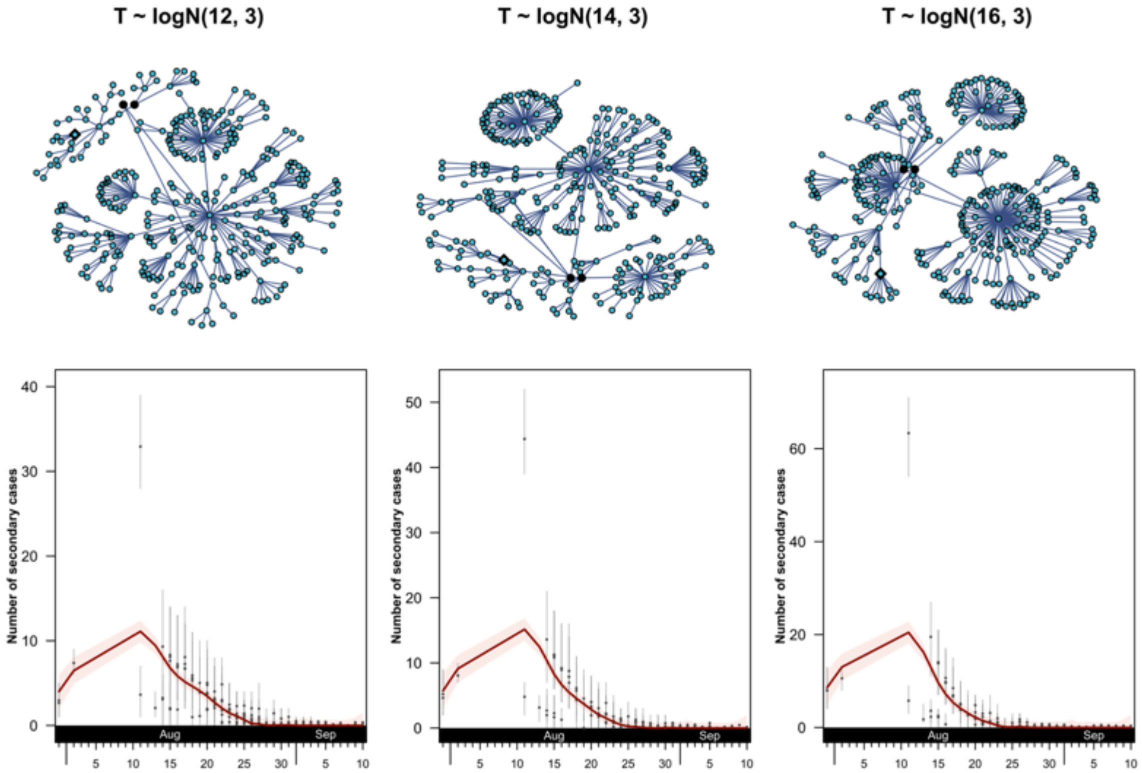

Supplementary Figure 6: *Work-alone* model.

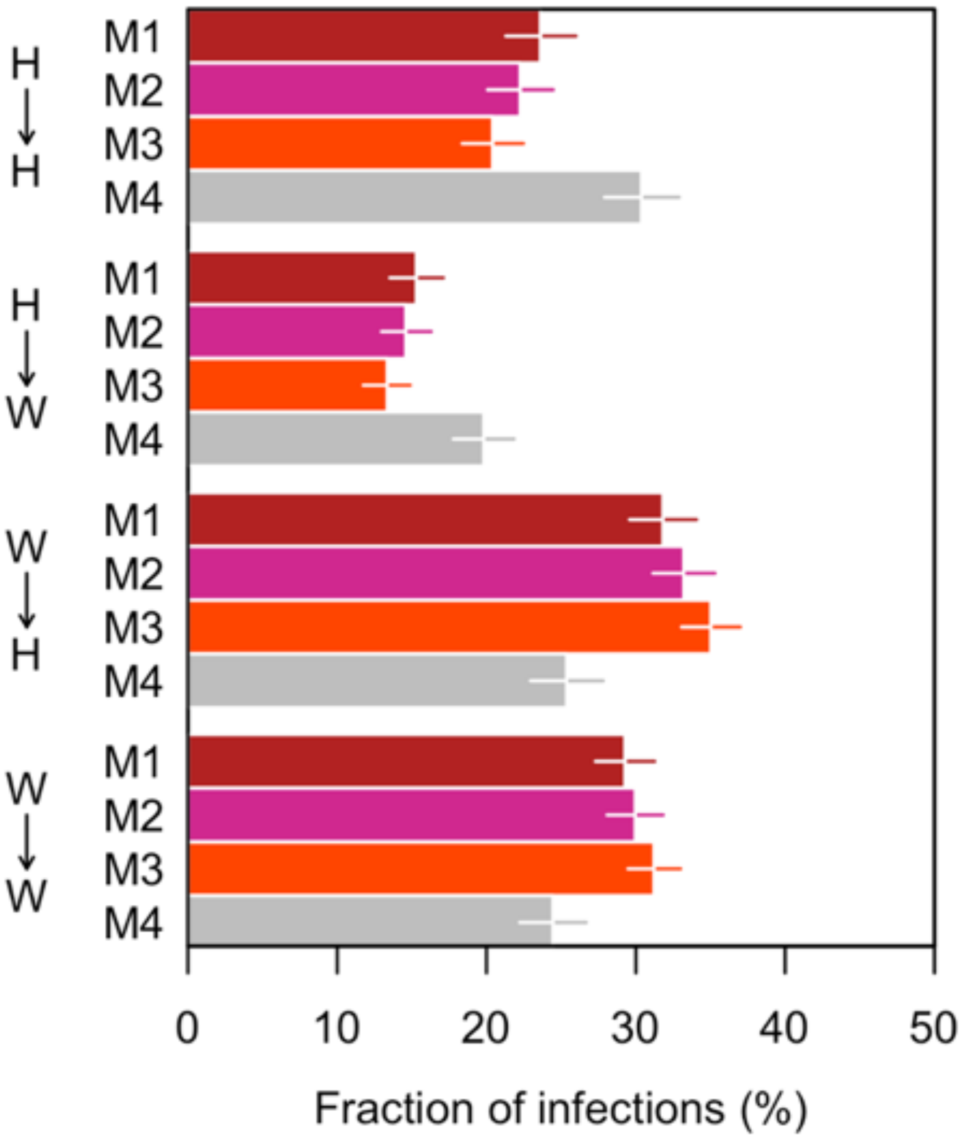

Supplementary Figure 7: Estimated proportion of cases attributable to transmission (indicated by arrows) near the donor's home (H) or work (W) and the recipients H or W, for four variants of the model. The temporal kernel is proportional to M1: a $\log N(12,3)$ density; M2: a $\log N(14,3)$; M3L a $\log N(16,3)$; and M4: a log-normal density with parameters estimated from the data.

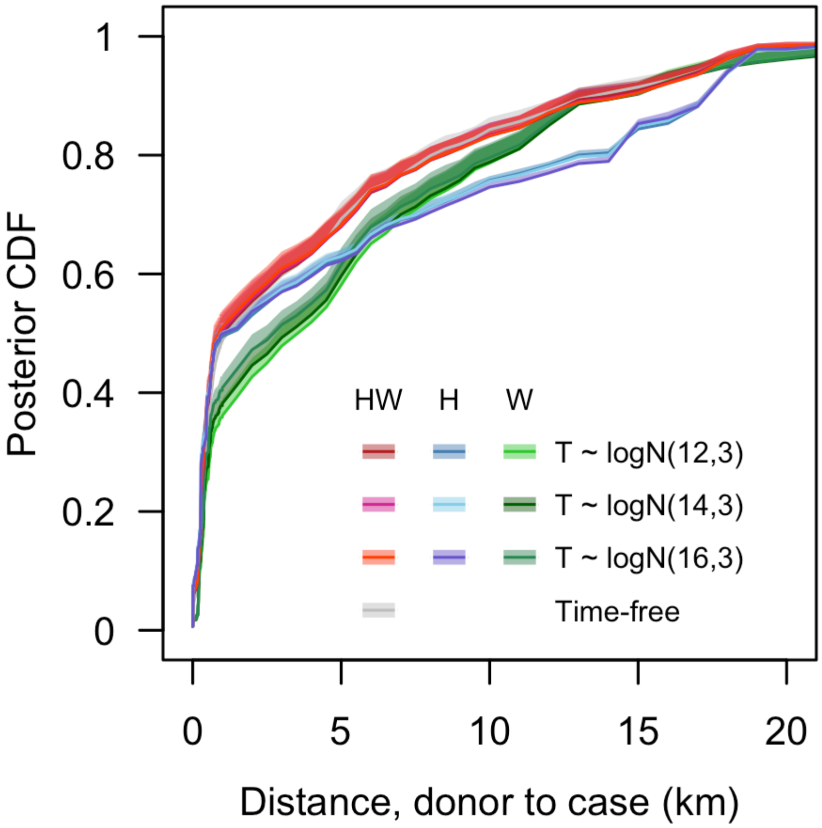

Supplementary Figure 8: Posterior cumulative distribution function (CDF) of distance from donor to recipient, under ten temporal kernels. Lines represent posterior means; shaded polygons represent 95% credible intervals. Reds represent the model in which transmission is allowed to happen at home and work; blues at home alone; and greens at work alone. In grey (lying near the red curves) is the model in which infection may happen at home and work and in which the temporal kernel has free parameters.

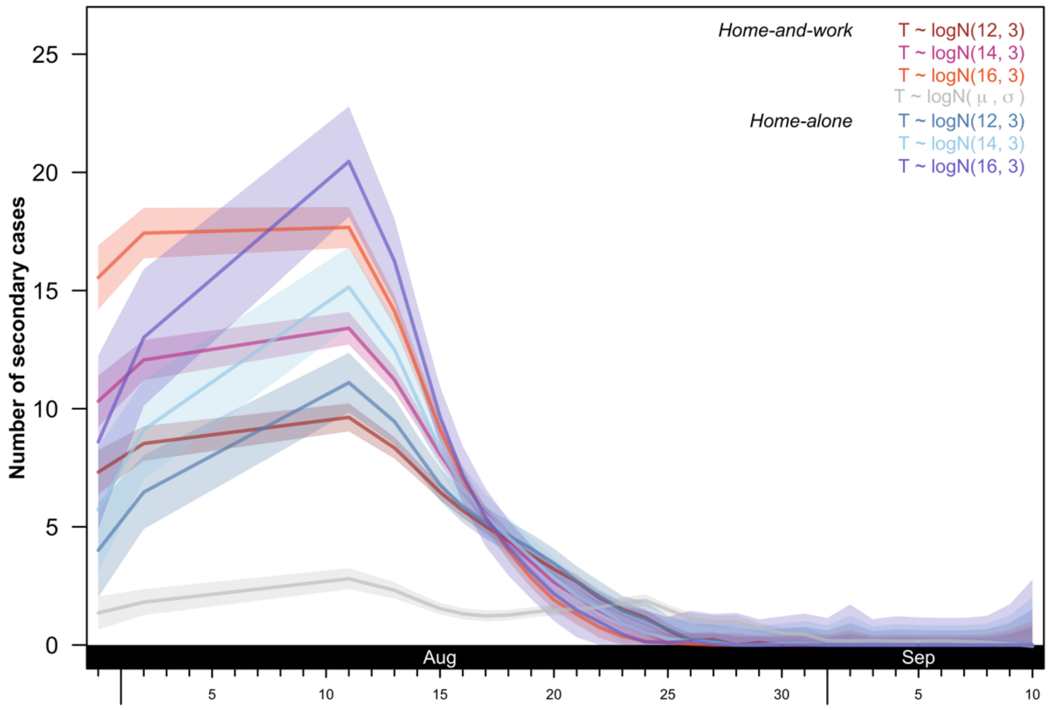

Supplementary Figure 9: Number of secondary infections against time.

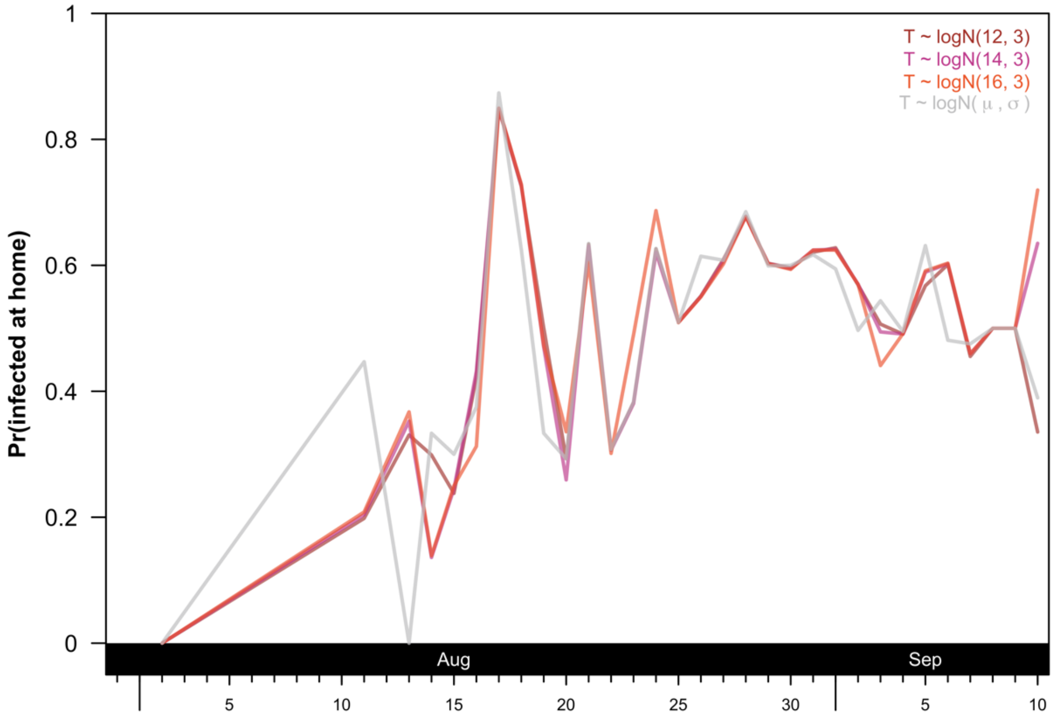

Supplementary Figure 10: Proportion of infections at home.

Simulation study

Simple simulation model. We then fitted the model to simulated data from the above algorithm and inferred the location of infection for each case as before, which were compared through a map for an arbitrary selection of cases (**Supplementary Figure 11**).

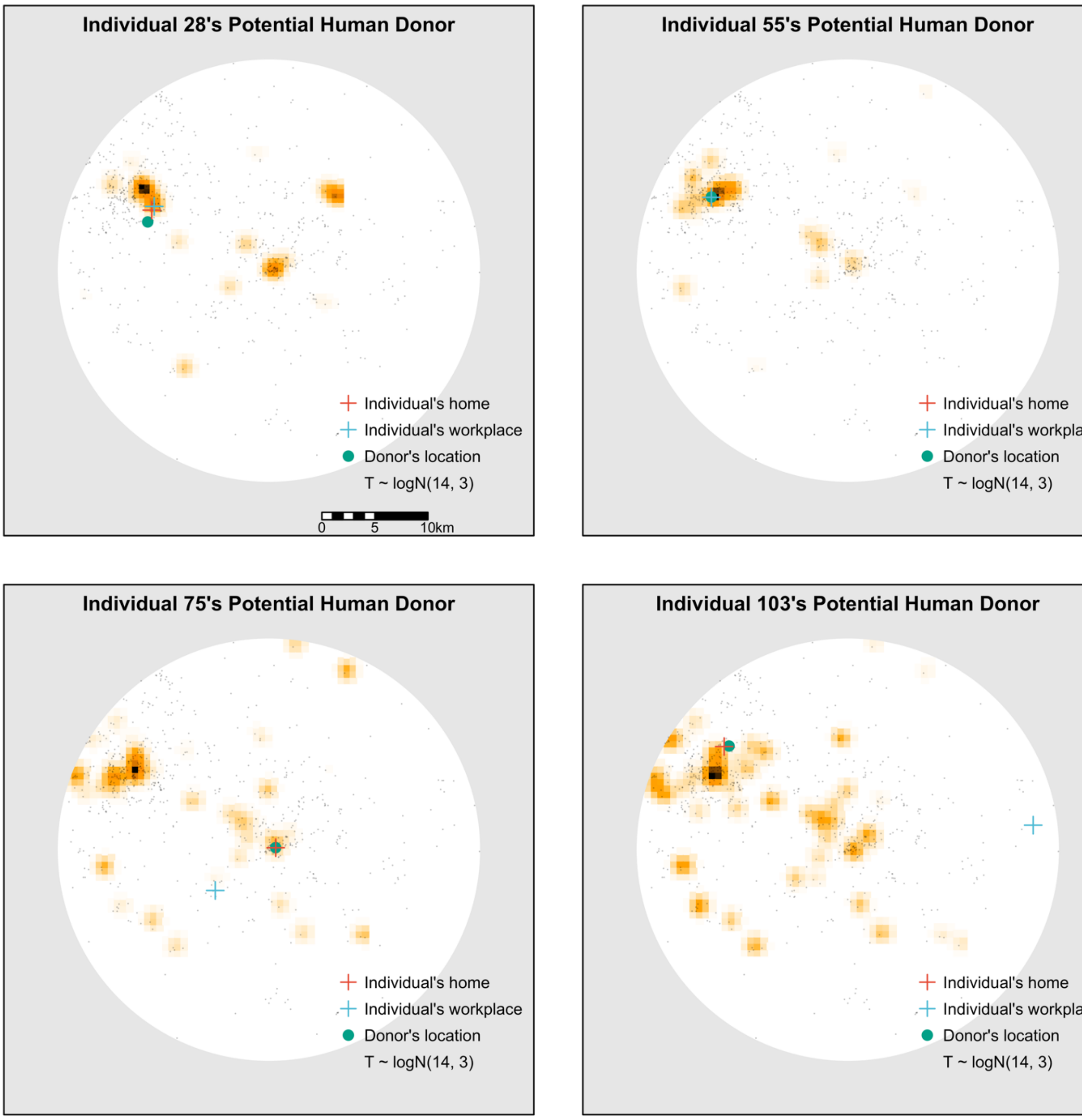

Supplementary Figure 11: Inferred infection location for four simulated cases from a simulated outbreak. The actual location of transmission is depicted with a circle, while the case's home and work are indicated by crosses. Orange regions represent the posterior distribution for the location of infection. The white area is the spatial domain of the simulations.

Extended simulation model. As can be seen, the generating model is more complex than the inferential model, including as it does differential rates of exposure at home and work, differential transmission rates at home and work, and a specific parametric form for the time-varying transmissibility. It therefore represents a reasonable challenge to the inferential routine that may be akin to the problems faced in analysing the actual dataset.

We inferred the donor (from among symptomatic cases) and location of infection, as described in the main paper. Because multiple symptomatic cases may be proximate in space and time, it is not possible to infer the exact source of infection, and as such, it is more reasonable to compare the inferred location than the individual causing infection. We inferred the location for all infected cases in the simulation, which we mapped in **Supplementary Figure 12** for an arbitrarily selected set of cases and in the supplementary files for all symptomatic cases. As can be seen, the inference is usually very good despite not knowing 80% of the actual infections.

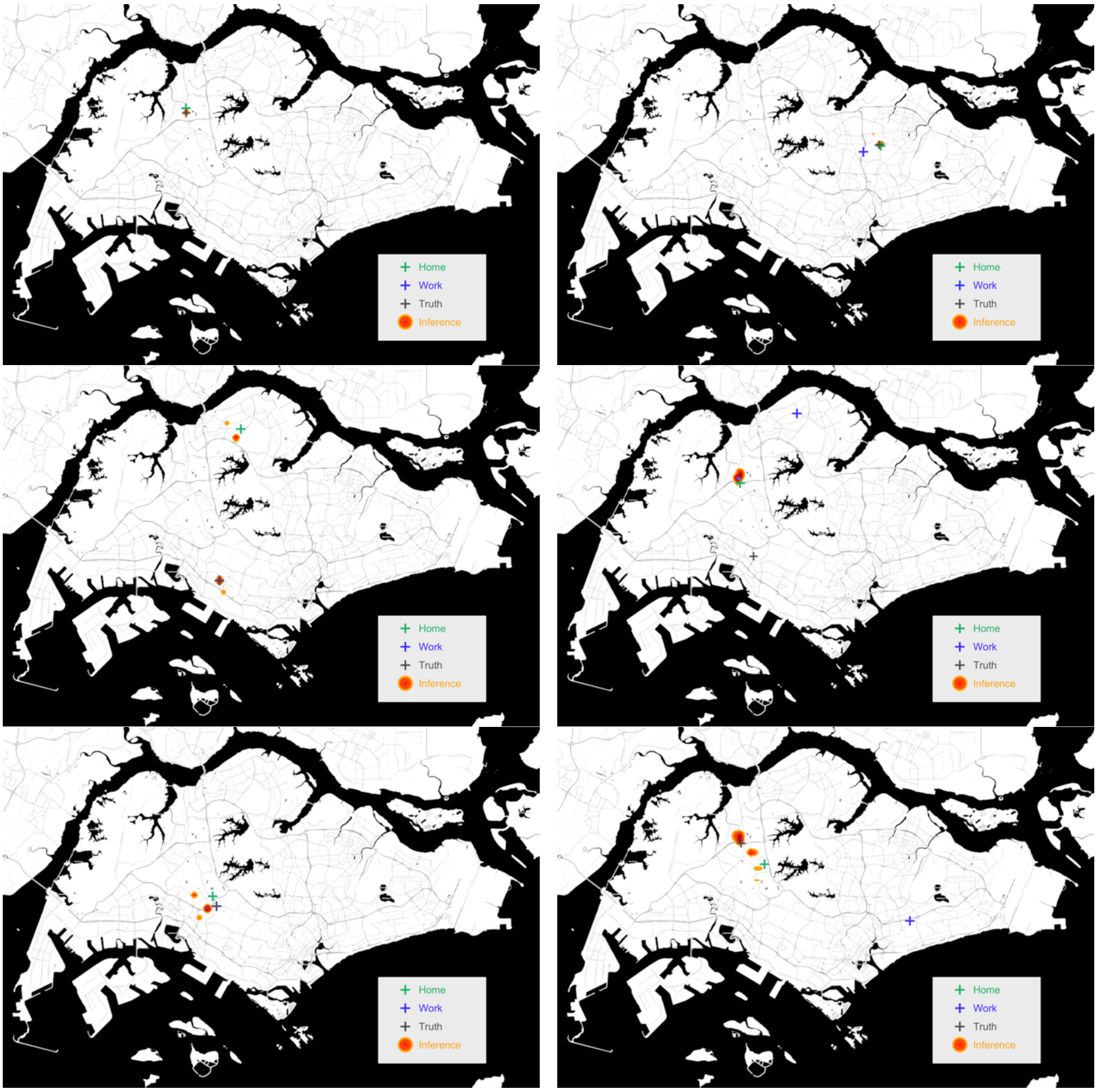

Supplementary Figure 12: Estimated location of infection for six arbitrary individuals (red heatmap). Home (green) and work (blue) locations are represented as crosses from the simulation study. The actual location of infection (gray) is also indicated by a cross. Other individuals in the simulation study are presented in the supplementary files.

We further compared the estimated distance from donor to recipient and compared to the actual distance distribution, using asymptomatic donors' locations where required, as presented in **Supplementary Figure 13**. The general features of the distance distribution are similar, though there is evidence that the actual distances tend to be longer.

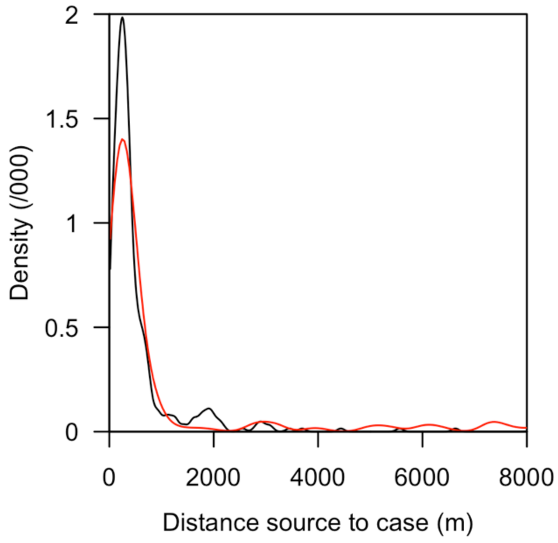

Supplementary Figure 13: The estimated distance between source and case (black) and actual distance (red) for all symptomatic cases from the simulation study.

We also quantified the proportion of cases infected at home or work, as well as the proportion of cases causing infection at home or work. We found that the inference routine can extremely accurately determine the fraction of cases infected near their home or work, but was not able to determine the corresponding fraction of cases infecting others at their home or work in the presence of severe censoring due to asymptomatic infections (**Supplementary Figure 14**).

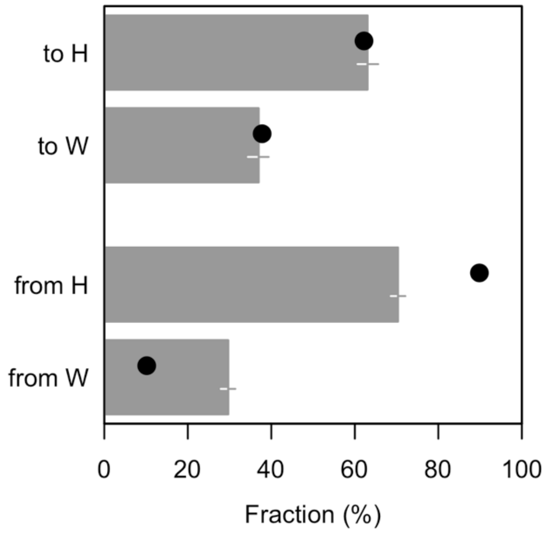

Supplementary Figure 14: Estimated proportion of cases infected at home (H) or work (W), or through the donor's home or work (grey) from the simulation study. The 95% credible intervals are grey/white whiskers. Black dots indicate the truth. In the presence of 80% asymptomatic cases, the distribution of secondary cases from the two location types cannot be accurately determined.

References

1. Department of Statistics Singapore. 2017 Statistics Singapore - Yearbook of Statistics, Singapore. See https://www.singstat.gov.sg/publications/publications-and-papers/reference/yearbook-of-statistics-singapore (accessed on 26 April 2018).

2. Ng L-C, Tan HK, Tan L-K, Chong CS, Ho D. 2016 Evolving dengue control programme in Singapore. *Epidemiol. News Bull.* **42**, 11–15.

3. Ooi E-E, Goh K-T, Gubler DJ. 2006 Dengue Prevention and 35 Years of Vector Control in Singapore. *Emerg. Infect. Dis.* **12**, 887–893. (doi:10.3201/10.3201/eid1206.051210)

4. Ang LW, Cutter J, James L, Goh KT. 2015 Seroepidemiology of dengue virus infection in the adult population in tropical Singapore. *Epidemiol. Infect.* **143**, 1585–1593. (doi:10.1017/S0950268814002507)

5. Ng LC, Lam S, Teo D. 2009 Epidemiology of dengue and chikungunya viruses and their potential impact on the blood supply. *ISBT Sci. Ser.* **4**, 357–367. (doi:10.1111/j.1751-2824.2009.01274.x)

6. Lee K-S, Lo S, Tan SS-Y, Chua R, Tan L-K, Xu H, Ng L-C. 2012 Dengue virus surveillance in Singapore reveals high viral diversity through multiple introductions and in situ evolution. *Infect. Genet. Evol. J. Mol. Epidemiol. Evol. Genet. Infect. Dis.* **12**, 77–85. (doi:10.1016/j.meegid.2011.10.012)

7. Stephenson JR. 2005 The problem with dengue. *Trans. R. Soc. Trop. Med. Hyg.* **99**, 643–646. (doi:10.1016/j.trstmh.2005.05.003)

8. Hapuarachchi HC *et al.* 2016 Epidemic resurgence of dengue fever in Singapore in 2013-2014: A virological and entomological perspective. *BMC Infect. Dis.* **16**. (doi:10.1186/s12879-016-1606-z)

9. Ng L-C *et al.* 2009 Entomologic and Virologic Investigation of Chikungunya, Singapore. *Emerg. Infect. Dis.* **15**, 1243–1249. (doi:10.3201/eid1508.081486)

10. Leo YS, Chow ALP, Tan LK, Lye DC, Lin L, Ng LC. 2009 Chikungunya Outbreak, Singapore, 2008. *Emerg. Infect. Dis.* **15**, 836–837. (doi:10.3201/eid1505.081390)

11. Ho ZJM *et al.* 2017 Outbreak of Zika virus infection in Singapore: an epidemiological, entomological, virological, and clinical analysis. *Lancet Infect. Dis.* **0**. (doi:10.1016/S1473-3099(17)30249-9)

12. Stoddard ST, Morrison AC, Vazquez-Prokopec GM, Paz Soldan V, Kochel TJ, Kitron U, Elder JP, Scott TW. 2009 The Role of Human Movement in the Transmission of Vector-Borne Pathogens. *PLoS Negl. Trop. Dis.* **3**, e481. (doi:10.1371/journal.pntd.0000481)

13. Barmak DH, Dorso CO, Otero M. 2016 Modelling dengue epidemic spreading with human mobility. *Phys. Stat. Mech. Its Appl.* **447**, 129–140. (doi:10.1016/j.physa.2015.12.015)

14. Cosner C, Beier JC, Cantrell RS, Impoinvil D, Kapitanski L, Potts MD, Troyo A, Ruan S. 2009 The effects of human movement on the persistence of vector-borne diseases. *J. Theor. Biol.* **258**, 550–560. (doi:10.1016/j.jtbi.2009.02.016)

15. Department of Statistics Singapore. 2010 Statistics Singapore - Census of Population 2010 Statistical Release 3 - Geographic Distribution and Transport. See

https://www.singstat.gov.sg/publications/publications-and-papers/cop2010/census10_stat_release3 (accessed on 3 April 2018).

16. Gelman A. 2014 *Bayesian data analysis*. Third edition. Boca Raton: CRC Press.

17. Gibson GJ, Renshaw E. 1998 Estimating parameters in stochastic compartmental models using Markov chain methods. *Math. Med. Biol. J. IMA* **15**, 19–40. (doi:10.1093/imammb/15.1.19)

18. Majumder MS, Cohn E, Fish D, Brownstein JS. 2016 Estimating a feasible serial interval range for Zika fever. *Bull. World Health Organ.* (doi:10.2471/BLT.16.171009)

19. Metropolis N, Rosenbluth AW, Rosenbluth MN, Teller AH, Teller E. 1953 Equation of State Calculations by Fast Computing Machines. *J. Chem. Phys.* **21**, 1087–1092. (doi:10.1063/1.1699114)

20. Hastings WK. 1970 Monte Carlo Sampling Methods Using Markov Chains and Their Applications. *Biometrika* **57**, 97–109. (doi:10.2307/2334940)

21. R Core Team. 2016 *R: A Language and Environment for Statistical Computing*. Vienna, Austria: R Foundation for Statistical Computing. See http://www.R-project.org.

22. QGIS Development Team. 2009 *QGIS Geographic Information System*. Open Source Geospatial Foundation. See http://qgis.osgeo.org.
