# Supplementary material for: Inferring who-infected-whom-where in the 2016 Zika outbreak in Singapore—a spatio-temporal model
Source: J R Soc Interface. 2019 Jun 19;16(155):20180604. doi: 10.1098/rsif.2018.0604 (PMC6597776; doi:10.1098/rsif.2018.0604)
Supplement: Additional Supplementary Figures [file rsif20180604supp2.pdf]

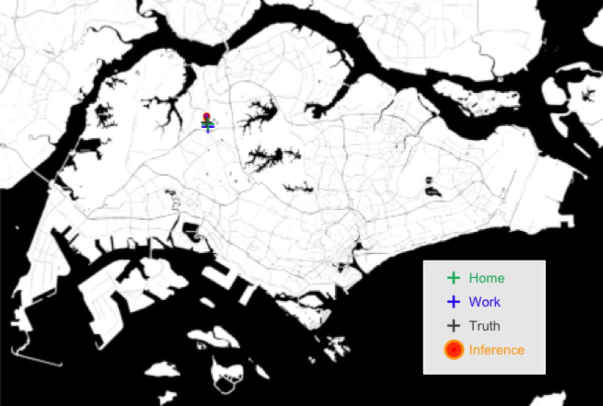
Home
Work
Truth
Inference

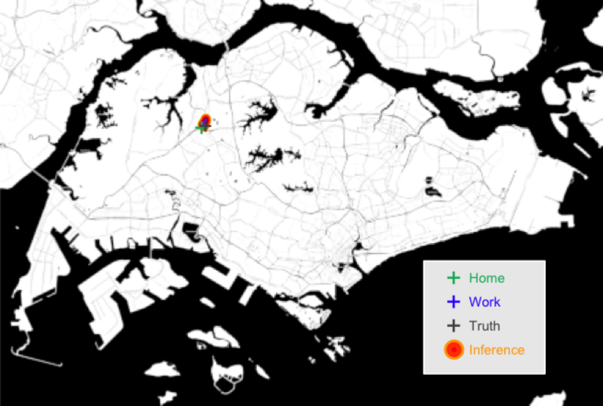

Home
Work
Truth
Inference

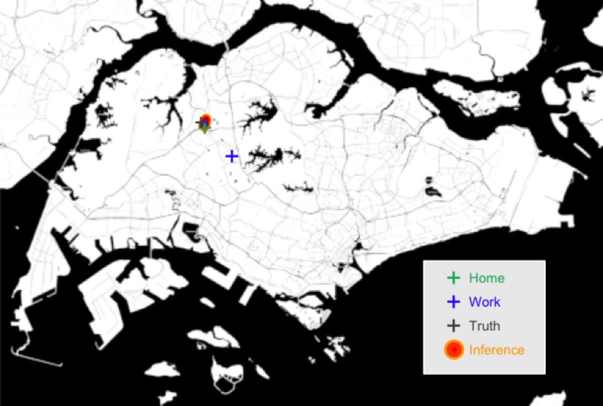
Home
Work
Truth
Inference

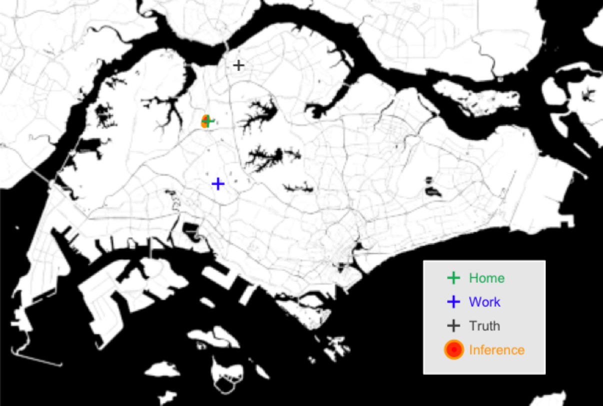
Home
Work
Truth
Inference

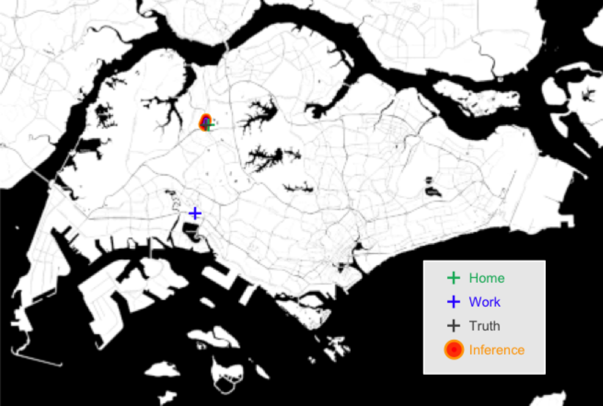
Home
Work
Truth
Inference

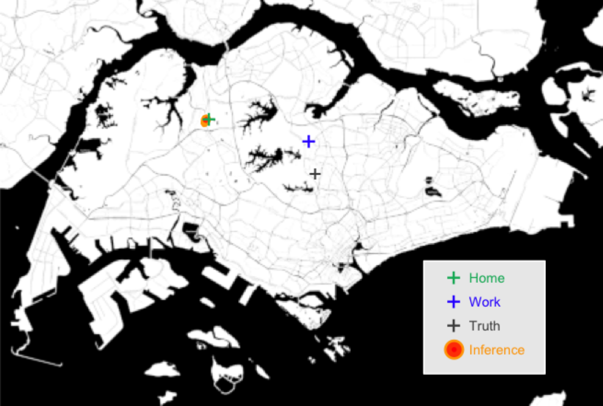
Home
Work
Truth
Inference

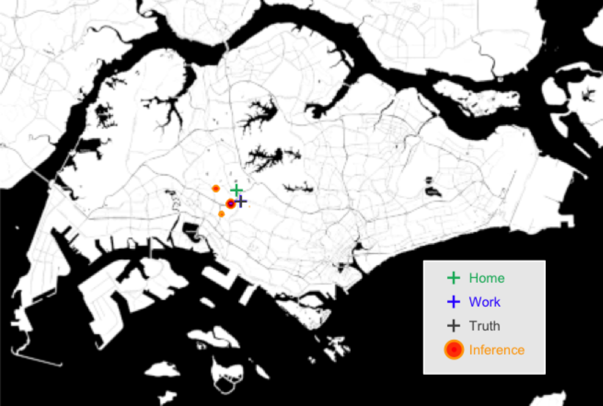
Home
Work
Truth
Inference

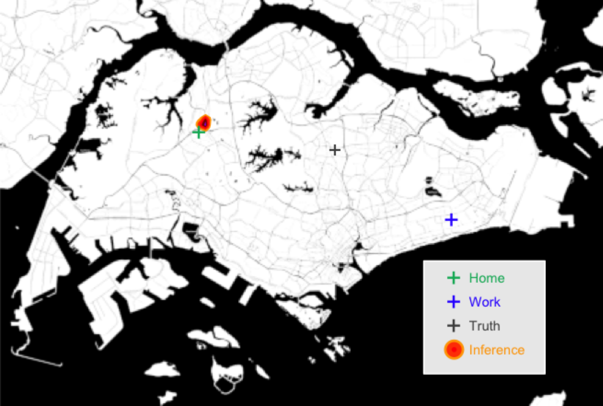

Home
Work
Truth
Inference

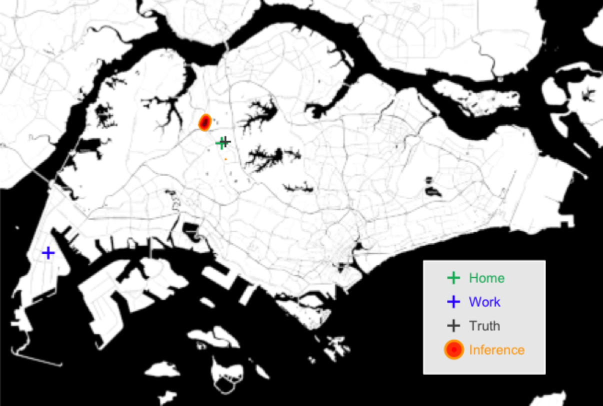
Home
Work
Truth
Inference

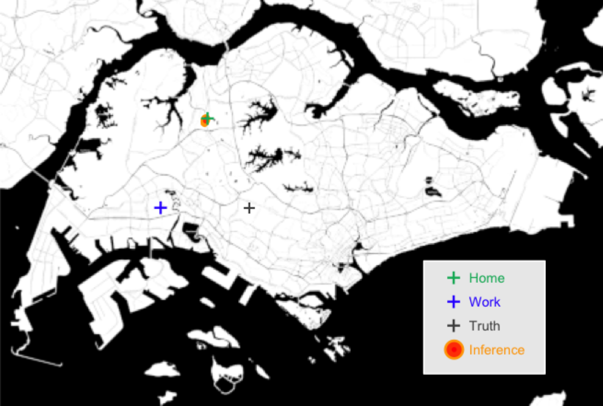
Home
Work
Truth
Inference

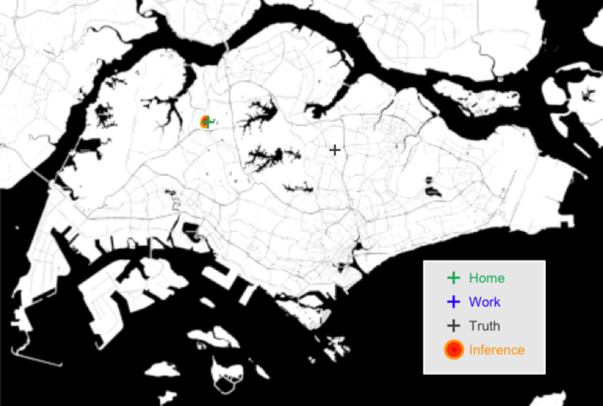
Home
Work
Truth
Inference

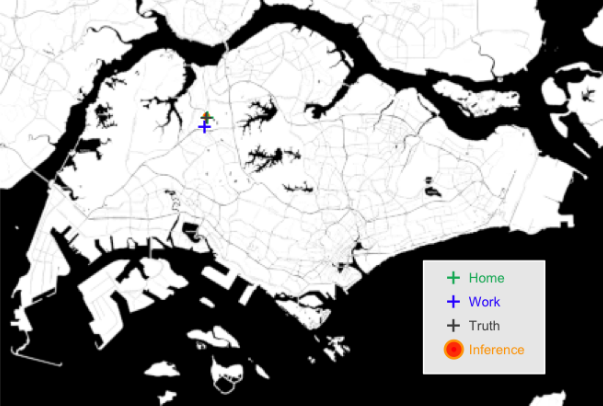
Home
Work
Truth
Inference

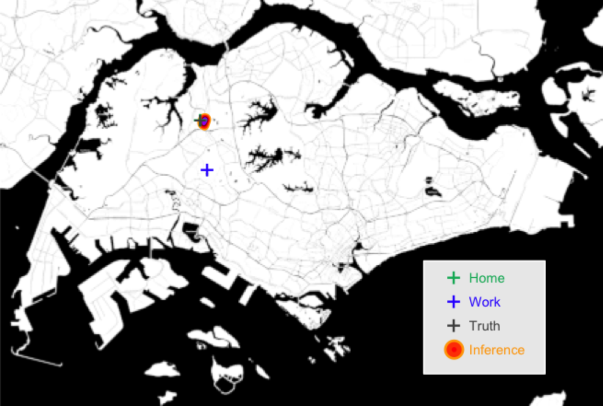

Home
Work
Truth
Inference

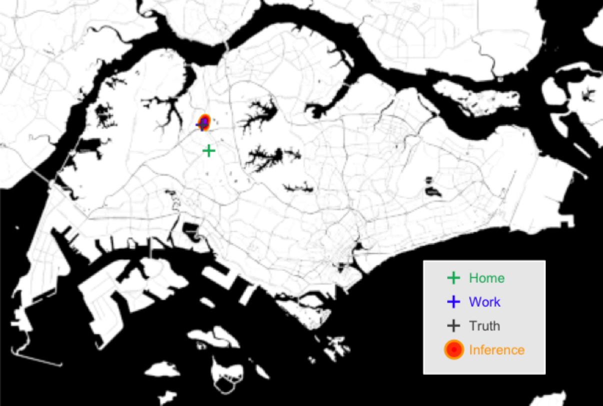
Home
Work
Truth
Inference

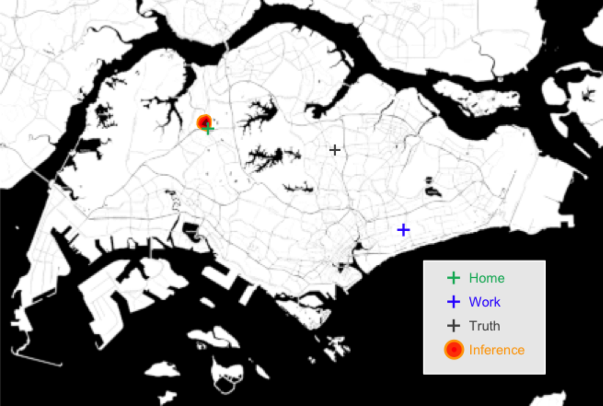
Home
Work
Truth
Inference

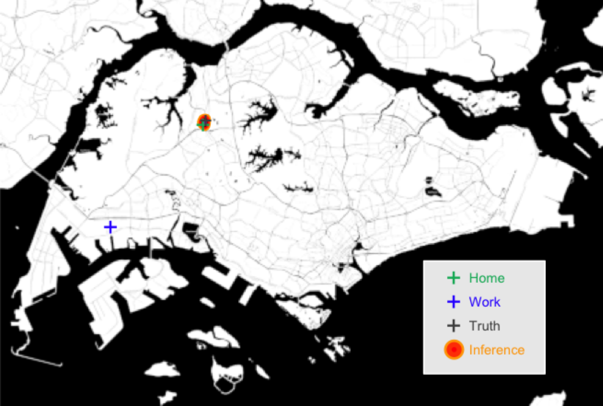
Home
Work
Truth
Inference

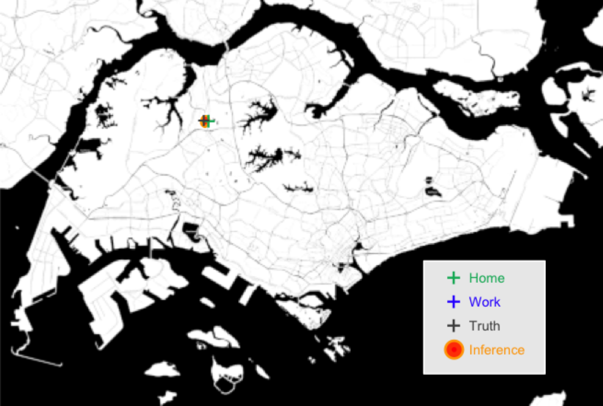
Home
Work
Truth
Inference

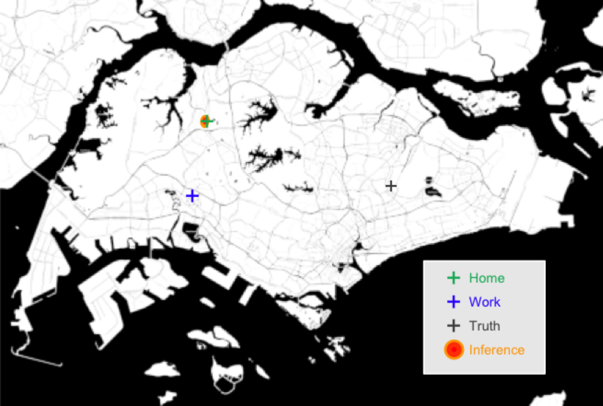
Home
Work
Truth
Inference

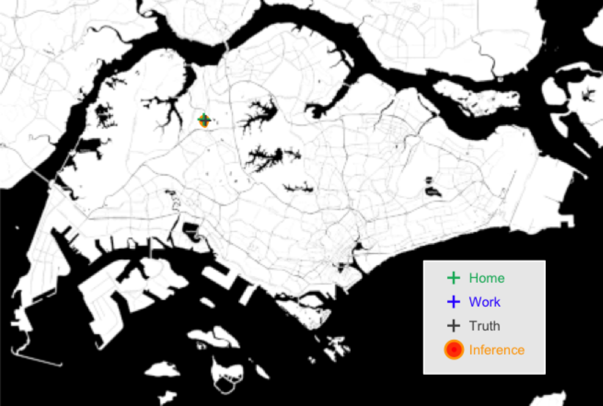
Home
Work
Truth
Inference

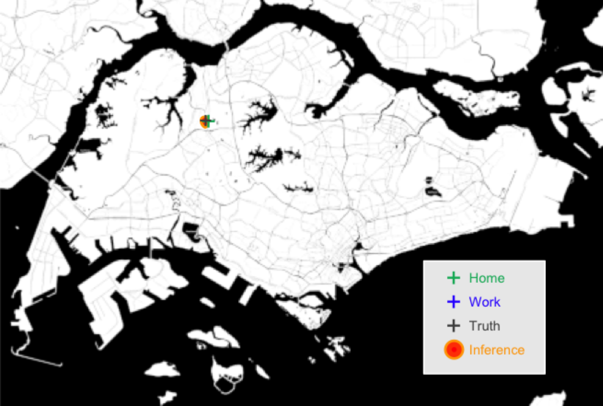

Home
Work
Truth
Inference

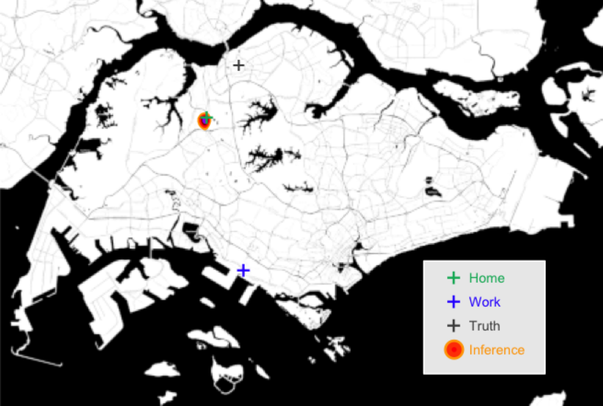
Home
Work
Truth
Inference

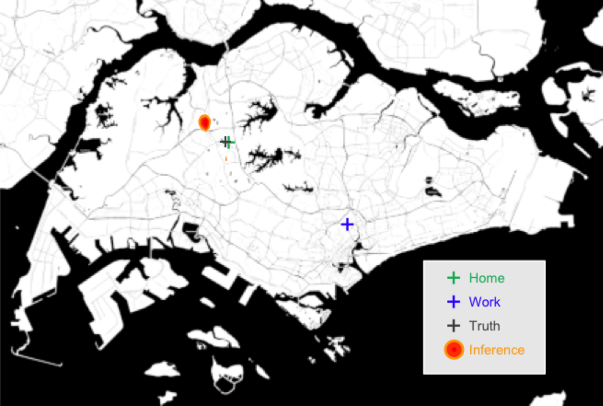

Home
Work
Truth
Inference

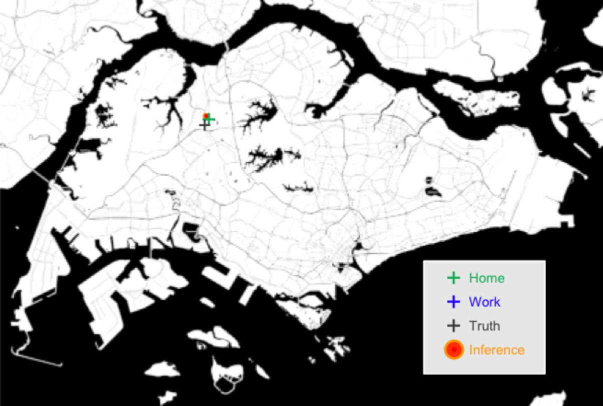
Home
Work
Truth
Inference

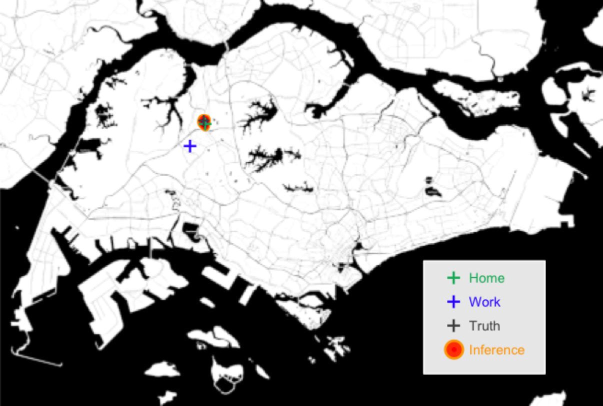
Home
Work
Truth
Inference

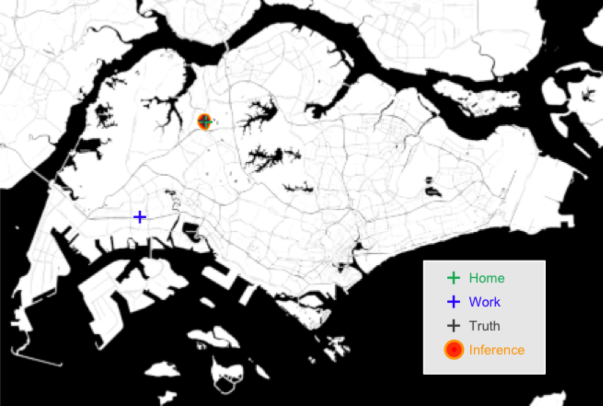
Home
Work
Truth
Inference

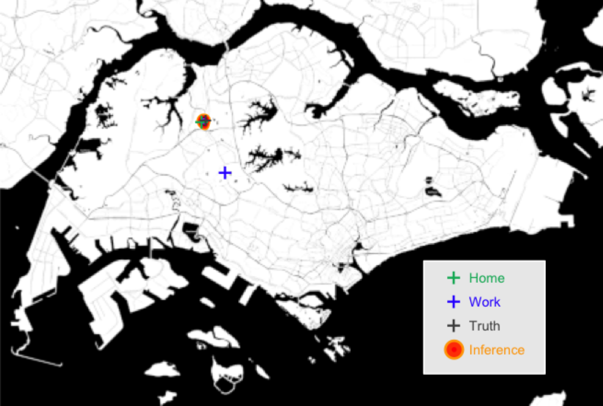
Home
Work
Truth
Inference

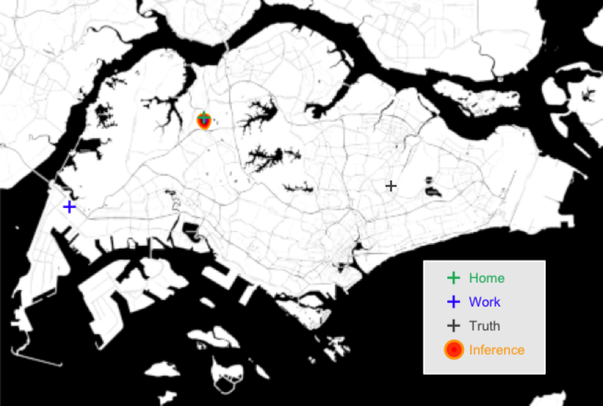

Home
Work
Truth
Inference

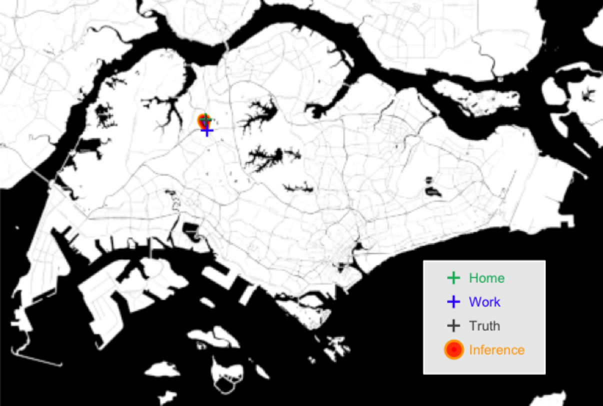
Home
Work
Truth
Inference

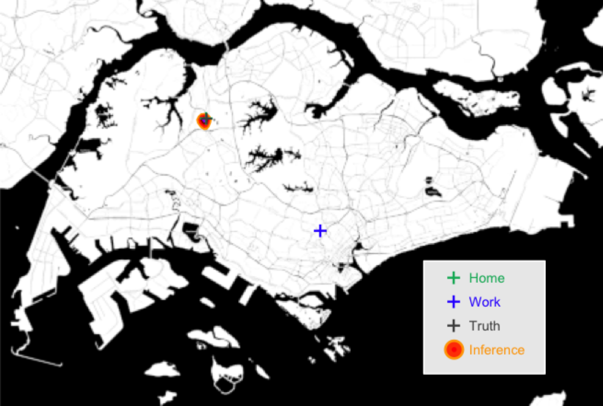
Home
Work
Truth
Inference

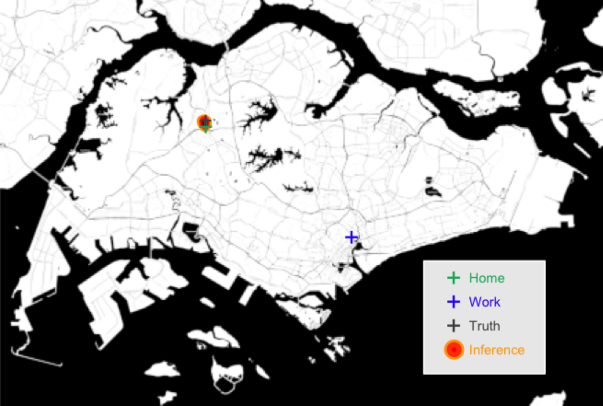
Home
Work
Truth
Inference

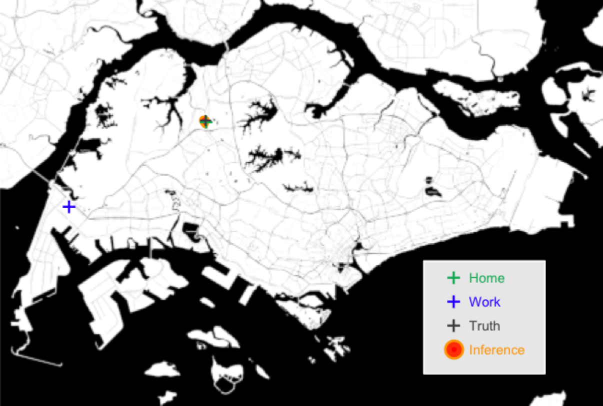
Home
Work
Truth
Inference

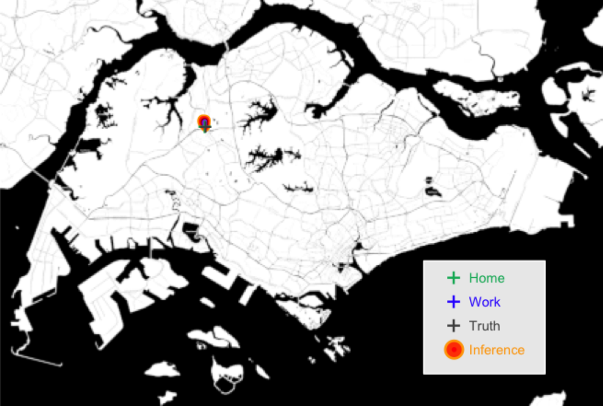
Home
Work
Truth
Inference

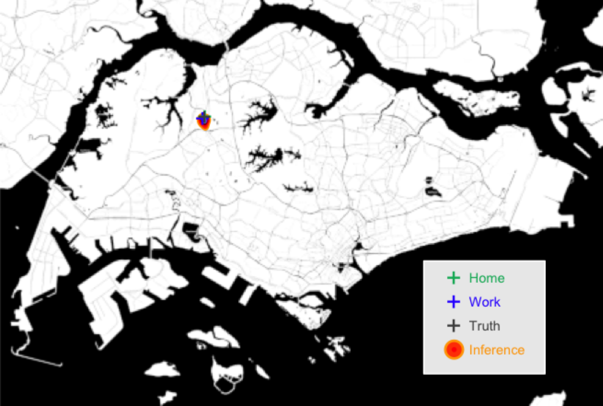
Home
Work
Truth
Inference

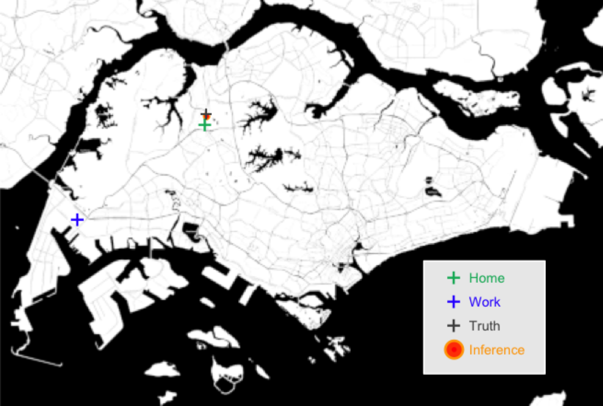
Home
Work
Truth
Inference

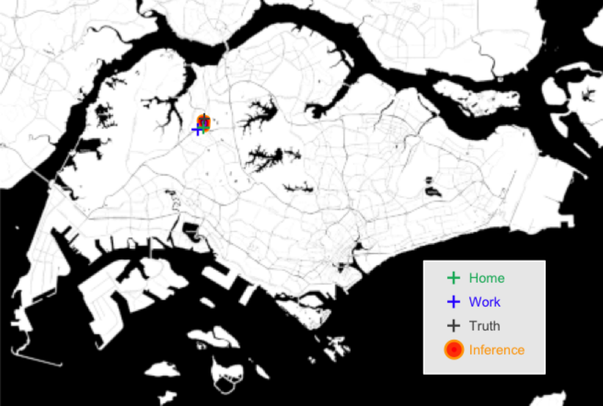
Home
Work
Truth
Inference

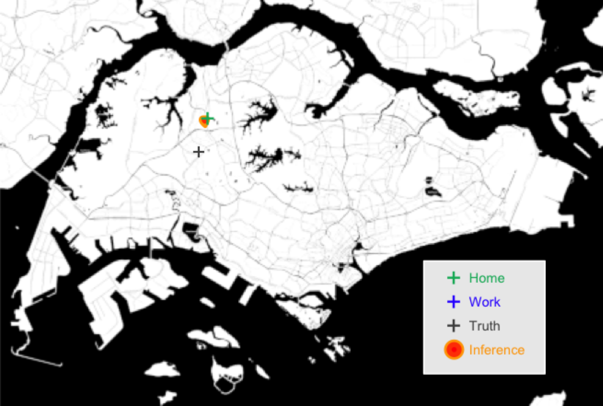

Home
Work
Truth
Inference

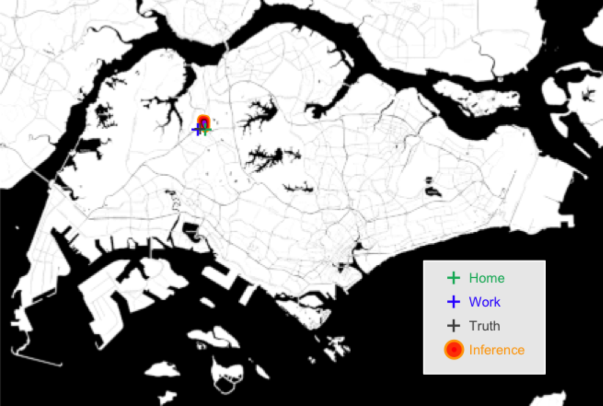
Home
Work
Truth
Inference

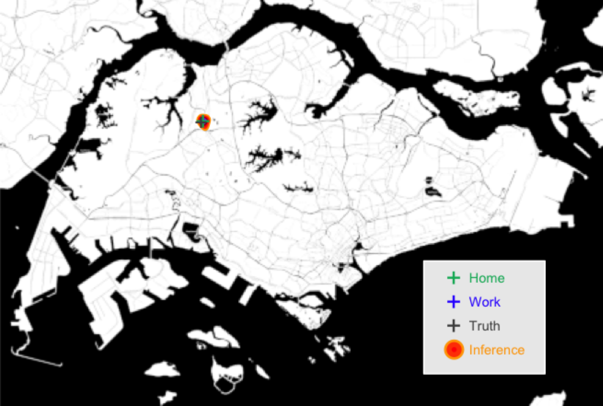

Home
Work
Truth
Inference

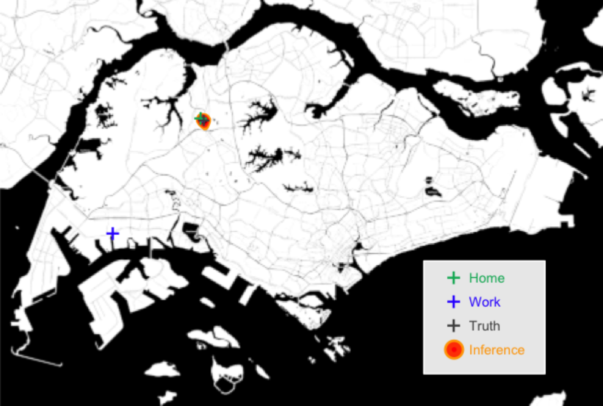
Home
Work
Truth
Inference

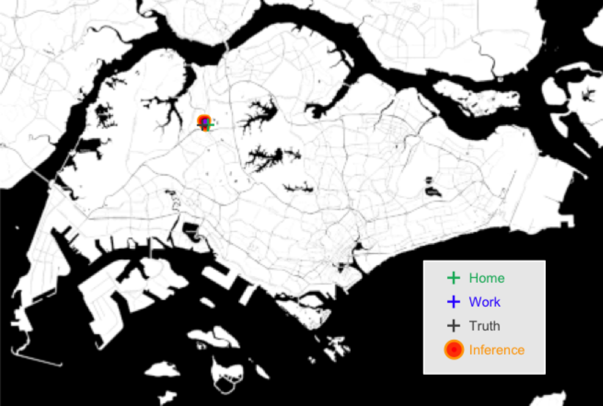
Home
Work
Truth
Inference

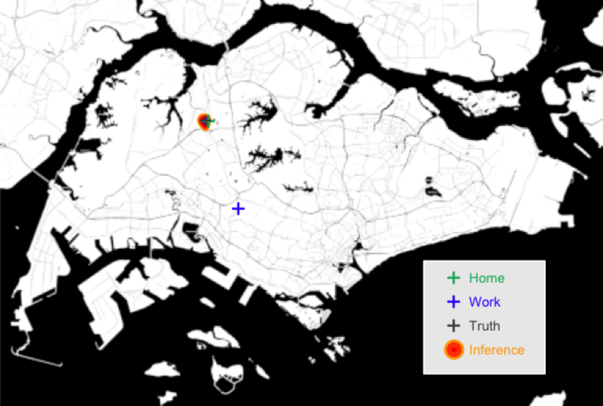
Home
Work
Truth
Inference

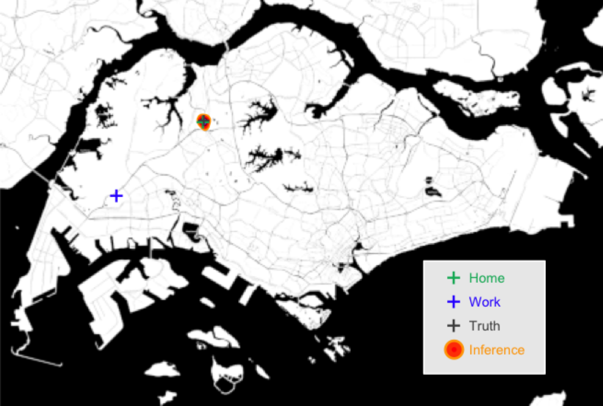
Home
Work
Truth
Inference

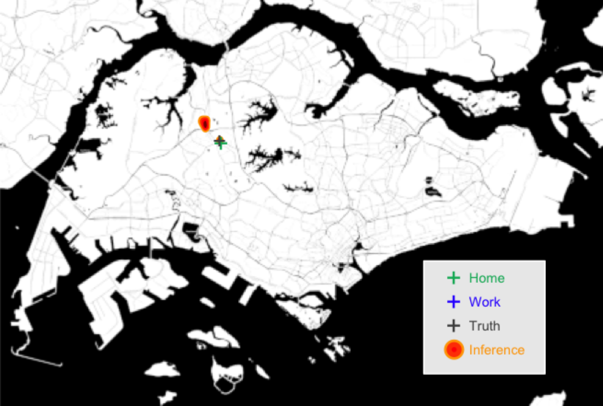
Home
Work
Truth
Inference

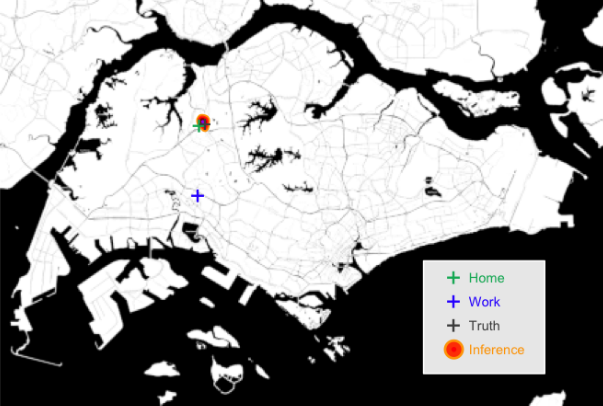
Home
Work
Truth
Inference

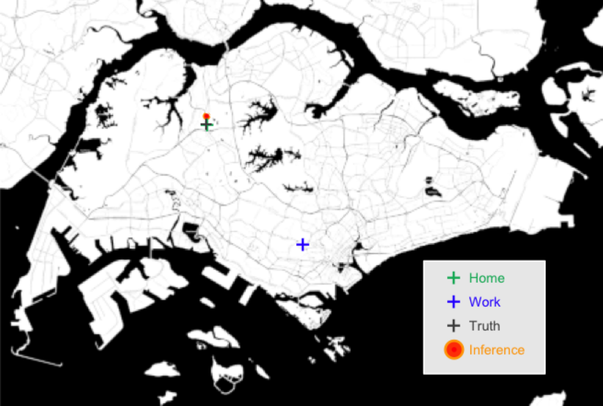
Home
Work
Truth
Inference

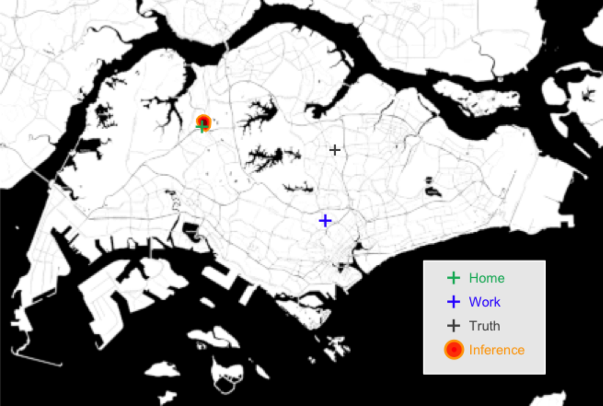
Home
Work
Truth
Inference

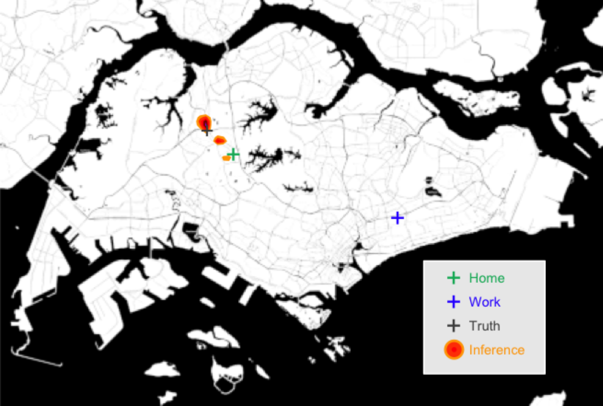
Home
Work
Truth
Inference

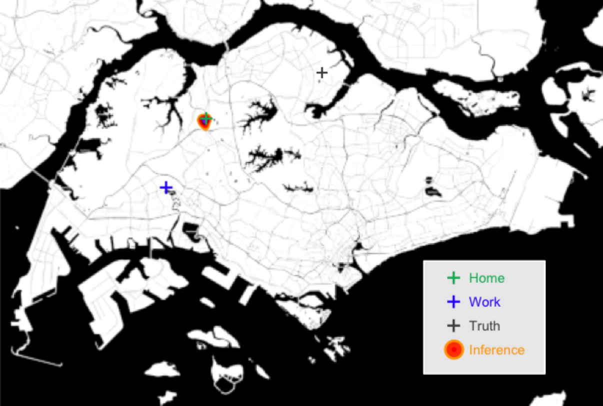
Home
Work
Truth
Inference

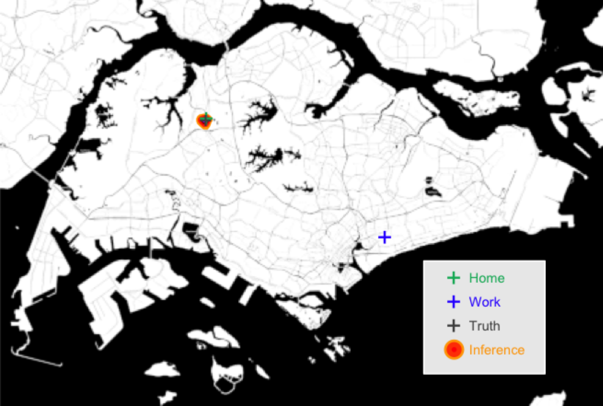
Home
Work
Truth
Inference

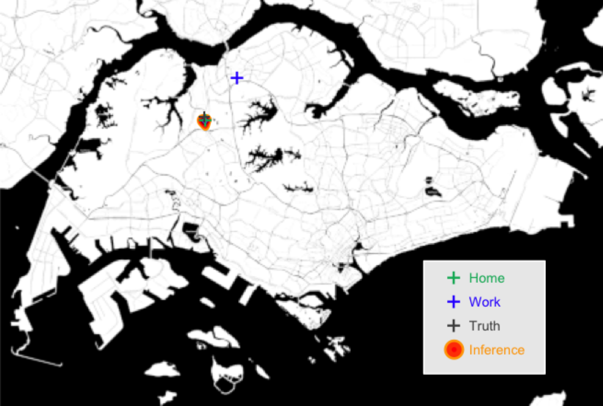

Home
Work
Truth
Inference

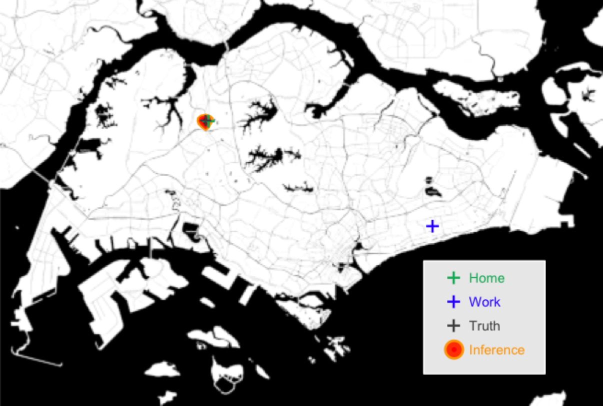
Home
Work
Truth
Inference

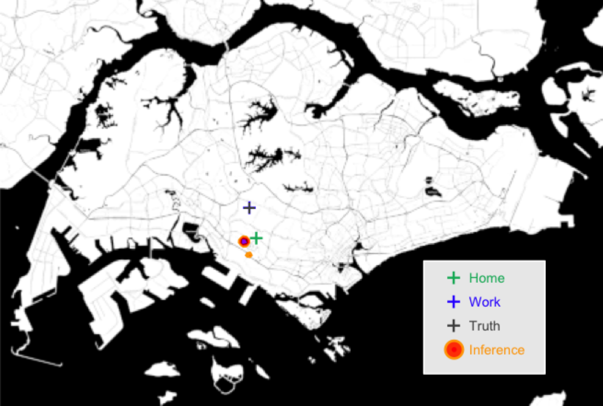
Home
Work
Truth
Inference

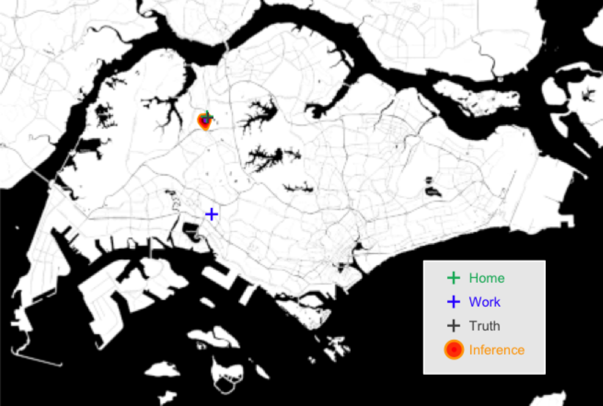
Home
Work
Truth
Inference

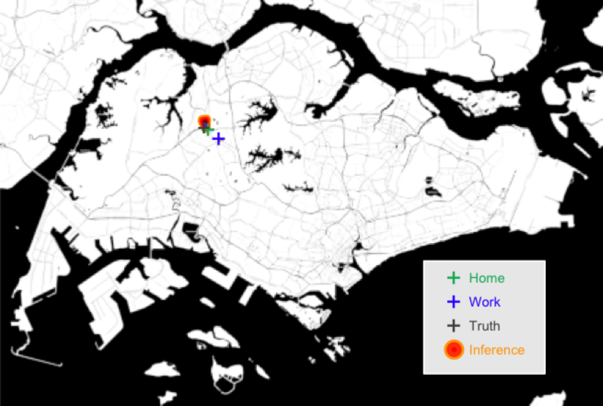
Home
Work
Truth
Inference

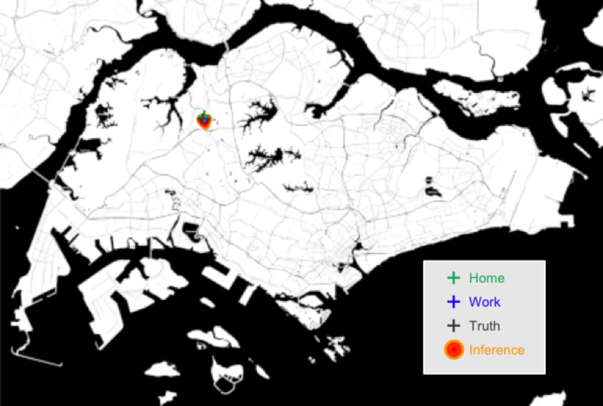
Home
Work
Truth
Inference

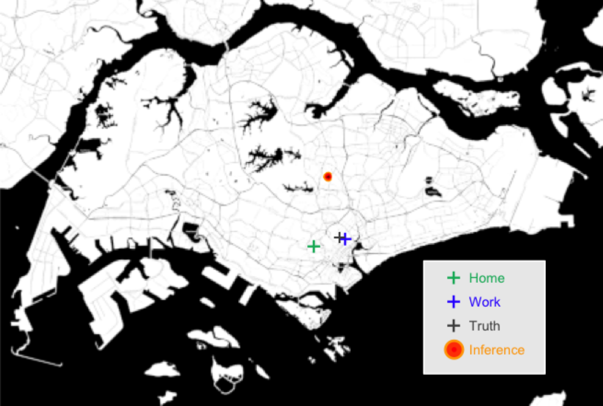
Home
Work
Truth
Inference

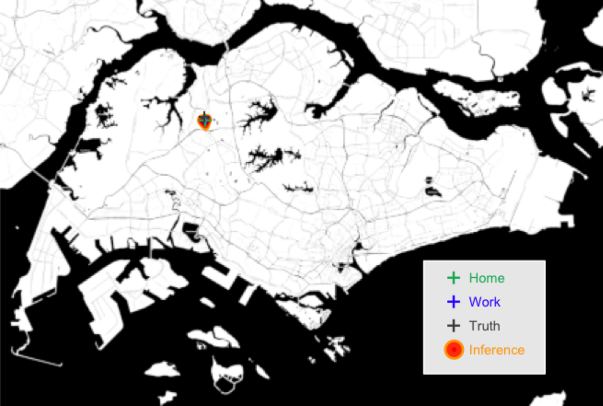
Home
Work
Truth
Inference

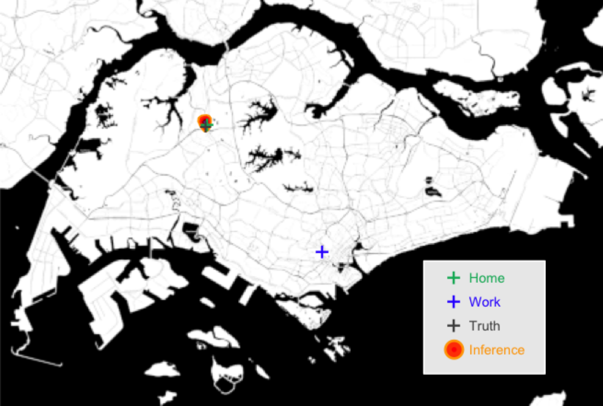
Home
Work
Truth
Inference

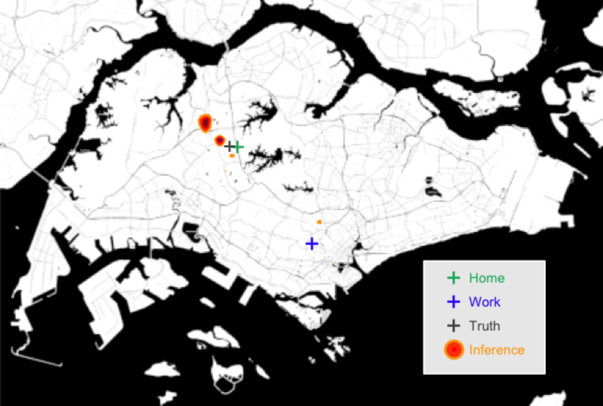
Home
Work
Truth
Inference

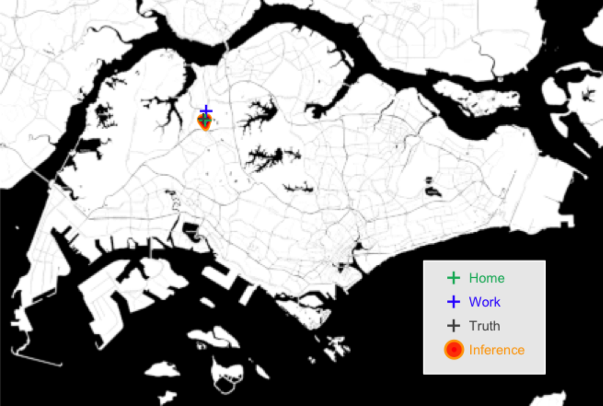
Home
Work
Truth
Inference

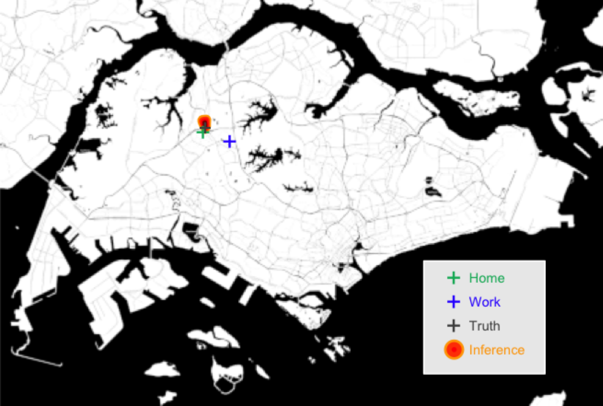
Home
Work
Truth
Inference

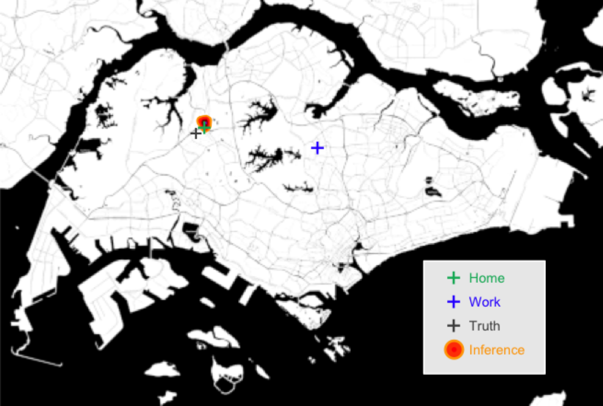
Home
Work
Truth
Inference

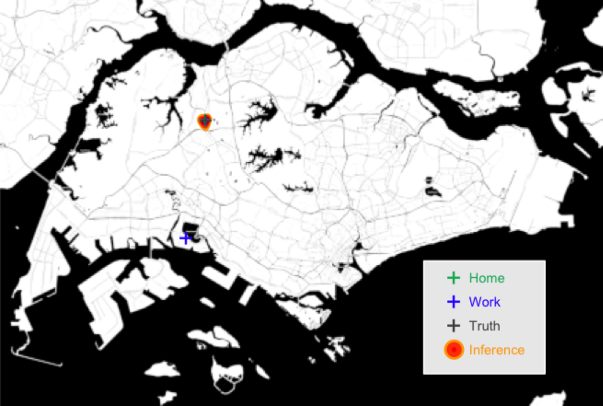
Home
Work
Truth
Inference

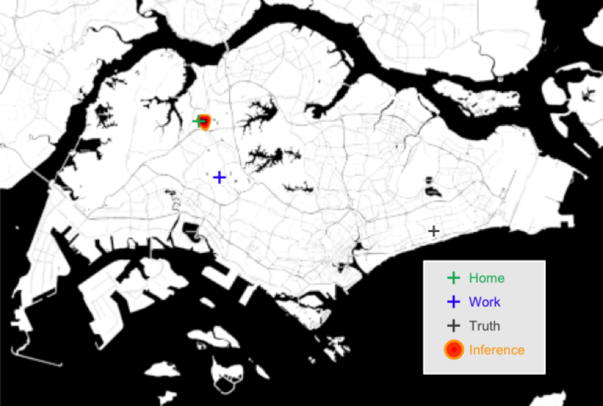
Home
Work
Truth
Inference

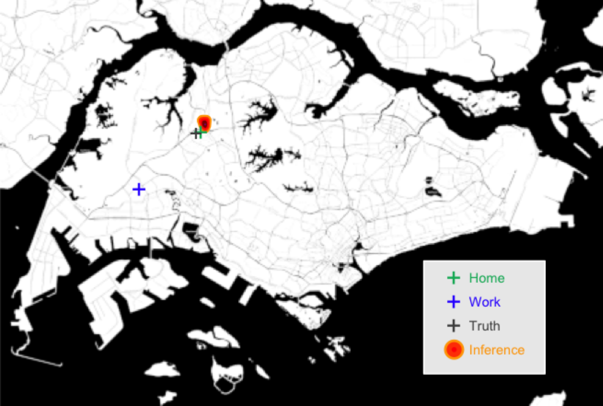
Home
Work
Truth
Inference

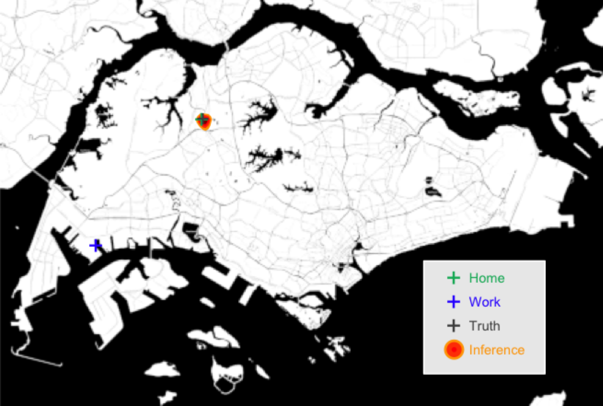

Home
Work
Truth
Inference

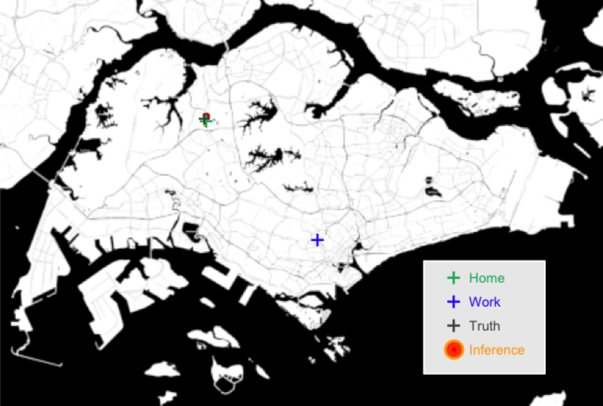
Home
Work
Truth
Inference

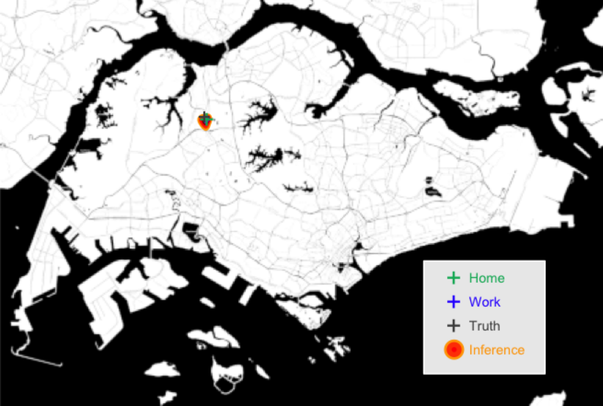
Home
Work
Truth
Inference

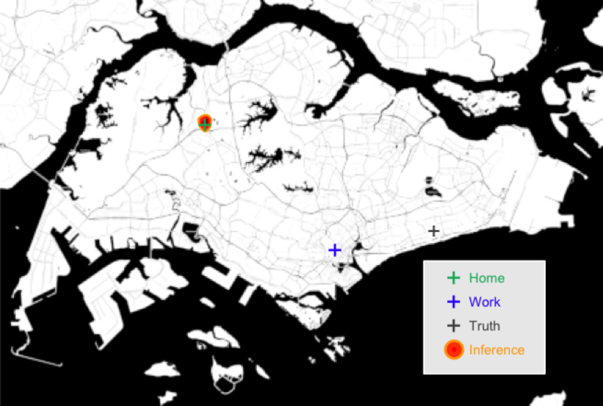
Home
Work
Truth
Inference

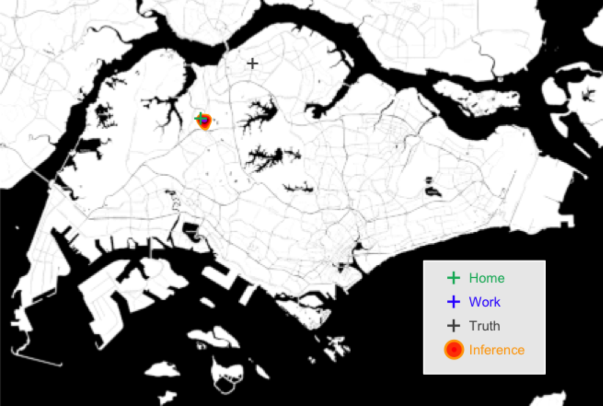
Home
Work
Truth
Inference

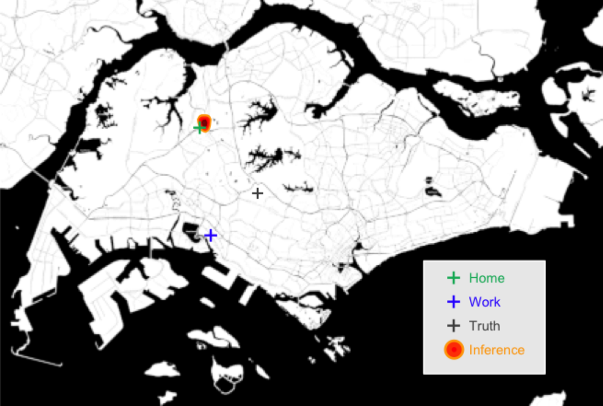

Home
Work
Truth
Inference

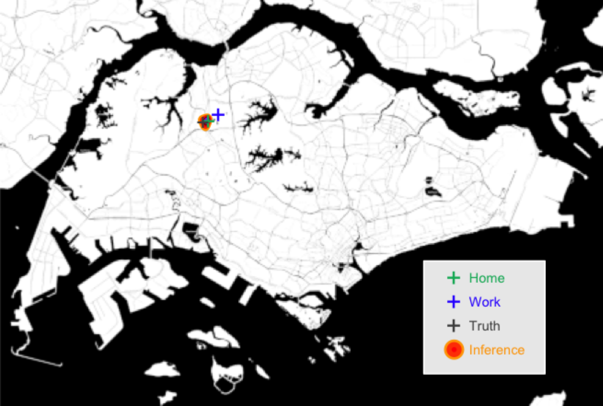
Home
Work
Truth
Inference

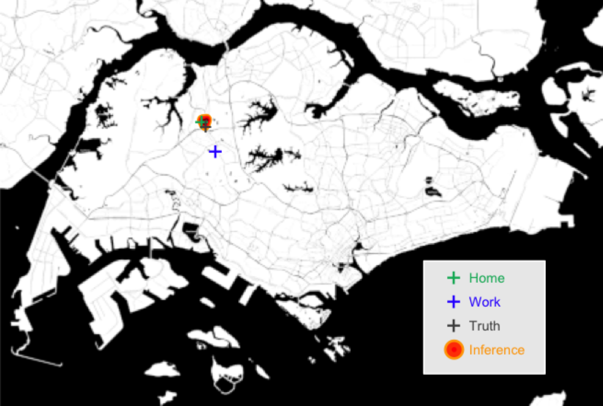
Home
Work
Truth
Inference

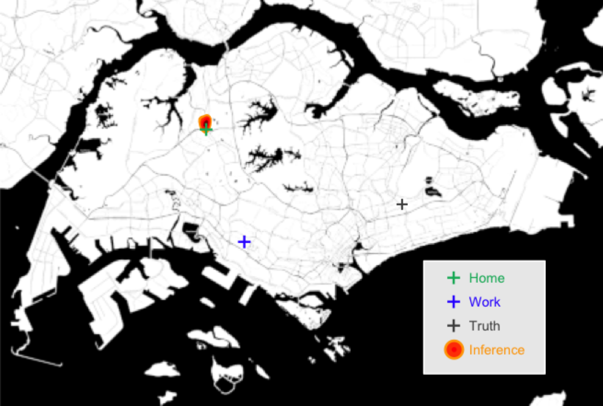
Home
Work
Truth
Inference

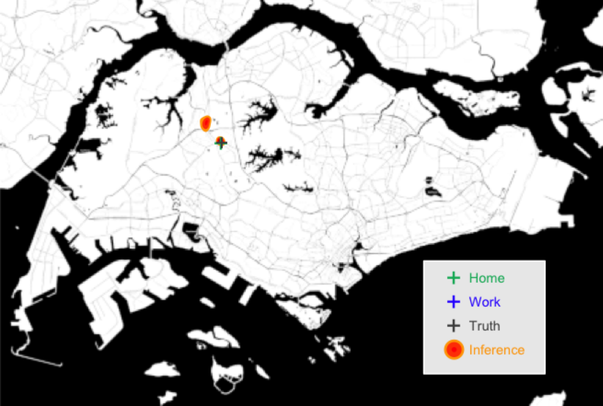

Home
Work
Truth
Inference

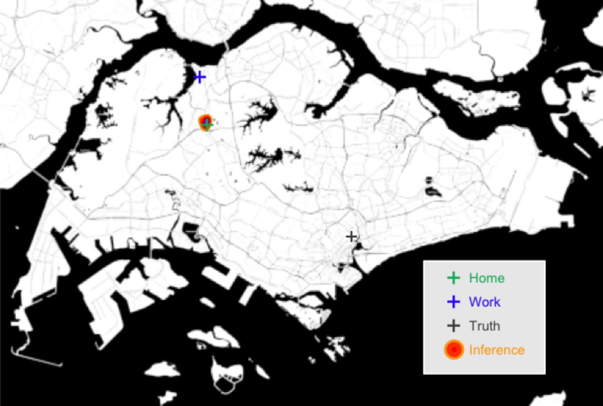
Home
Work
Truth
Inference

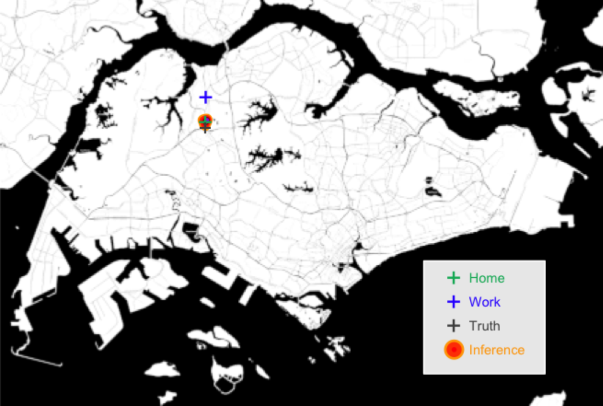
Home
Work
Truth
Inference

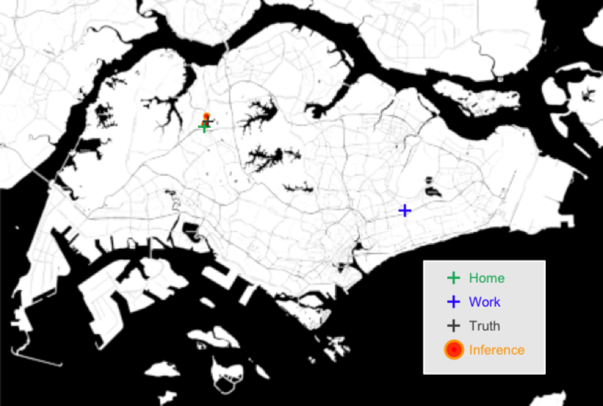

Home
Work
Truth
Inference

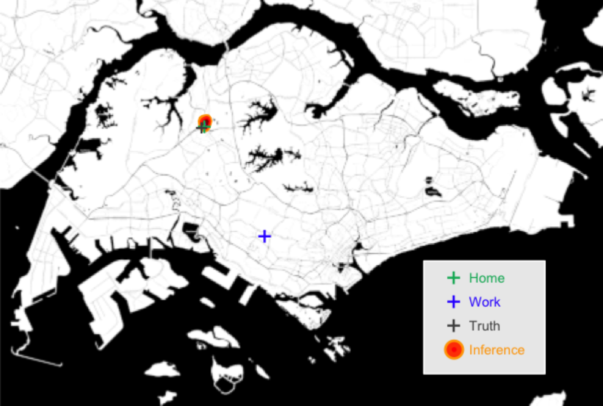
Home
Work
Truth
Inference

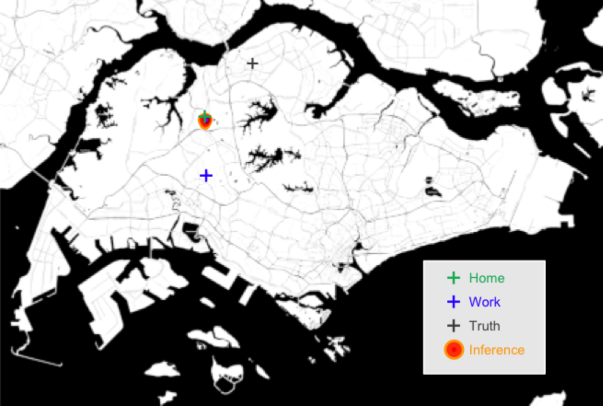
Home
Work
Truth
Inference

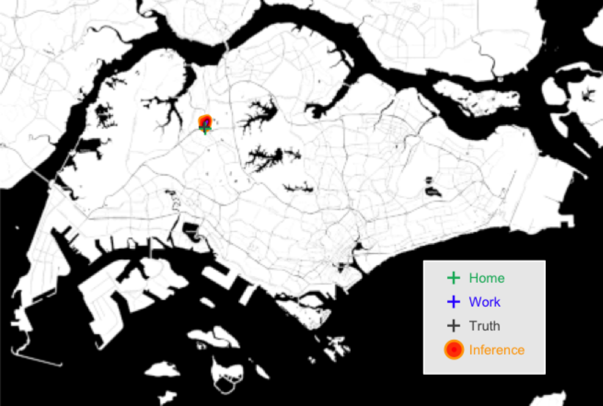

Home
Work
Truth
Inference

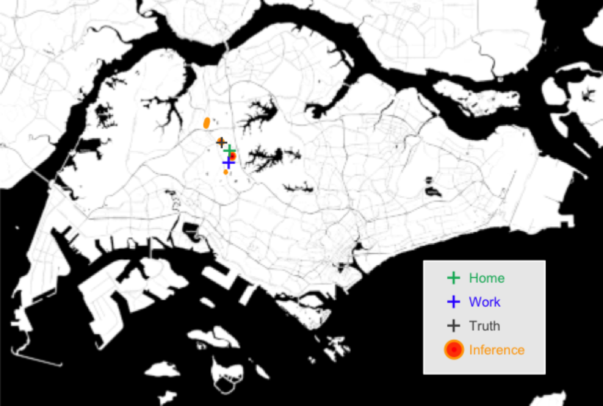
Home
Work
Truth
Inference

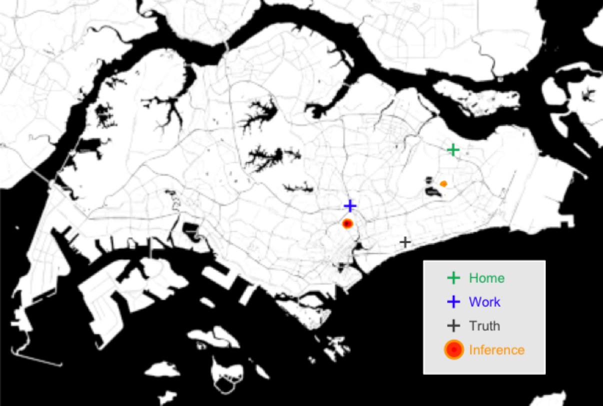
Home
Work
Truth
Inference

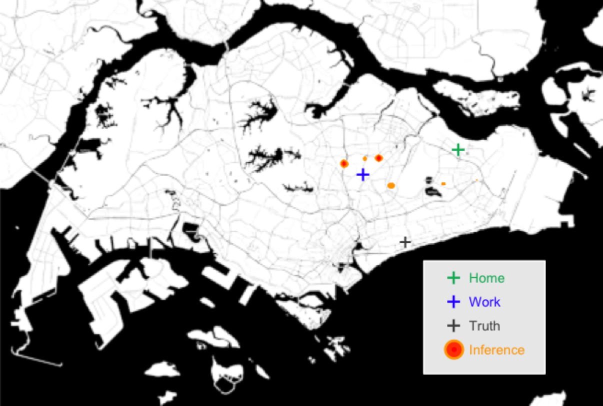
Home
Work
Truth
Inference

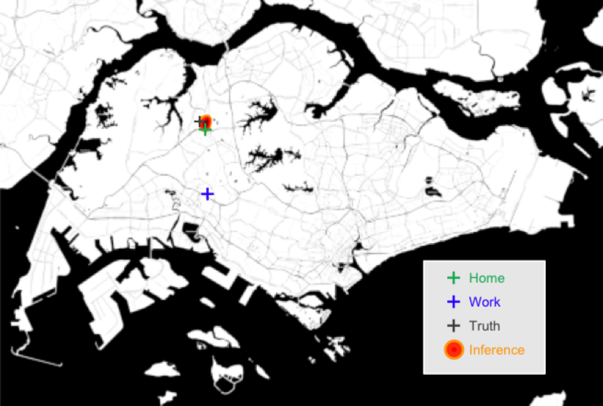
Home
Work
Truth
Inference

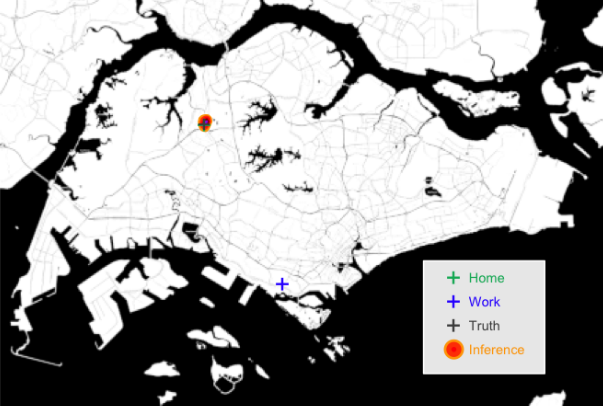
Home
Work
Truth
Inference

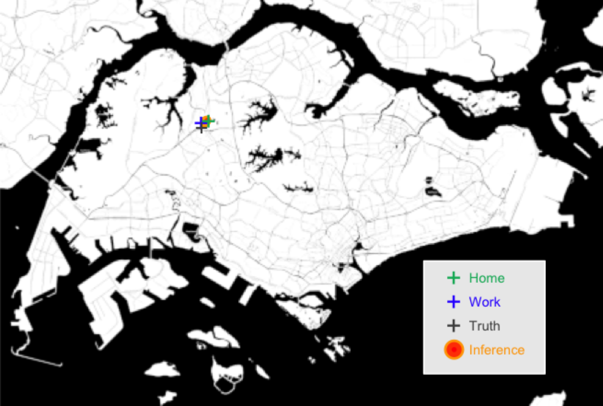
Home
Work
Truth
Inference

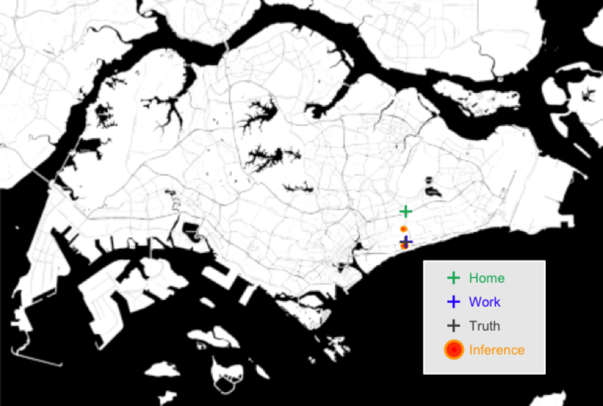
Home
Work
Truth
Inference

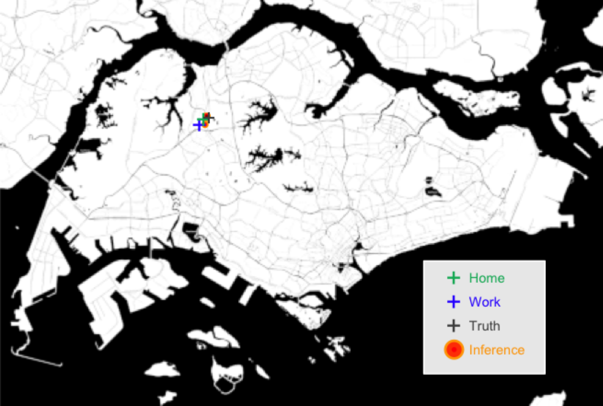
Home
Work
Truth
Inference

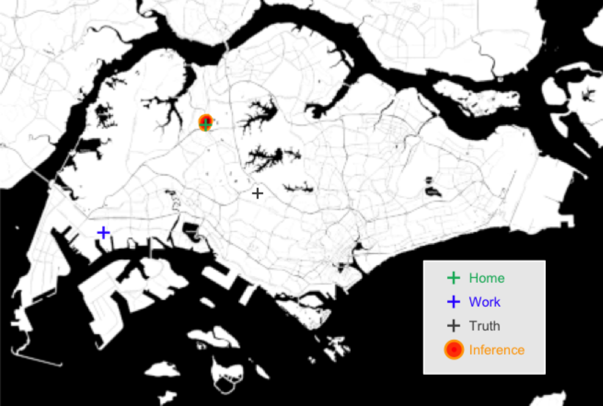
Home
Work
Truth
Inference

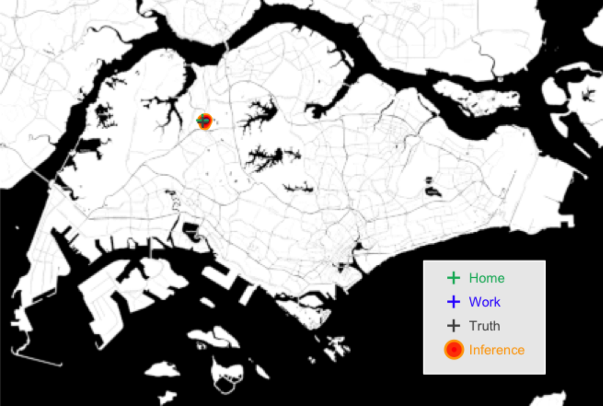
Home
Work
Truth
Inference

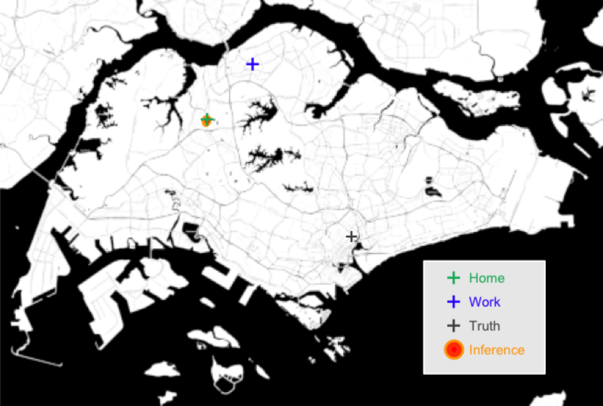
Home
Work
Truth
Inference

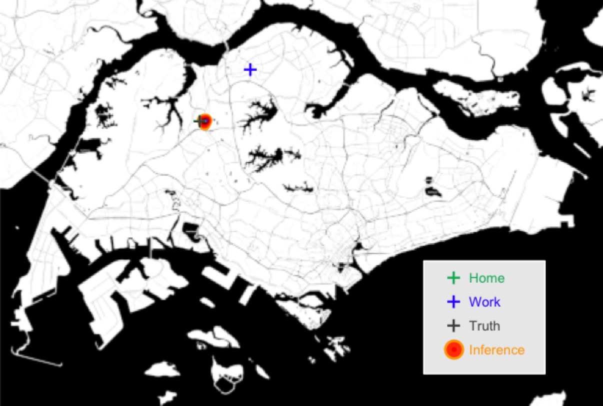
Home
Work
Truth
Inference

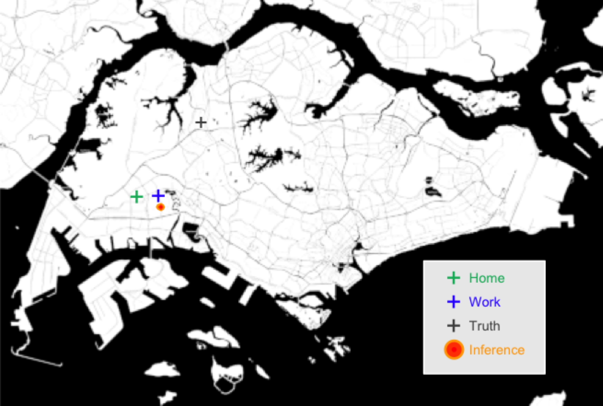

Home
Work
Truth
Inference

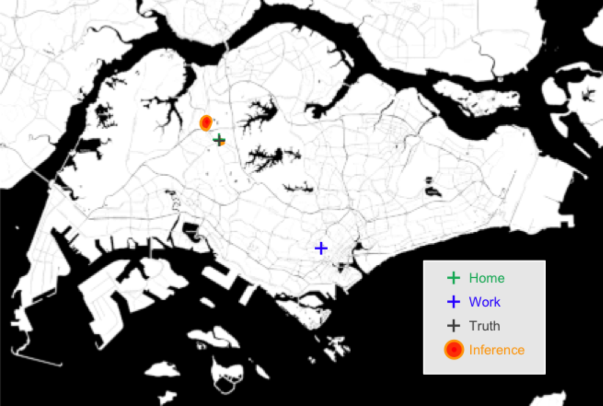
Home
Work
Truth
Inference

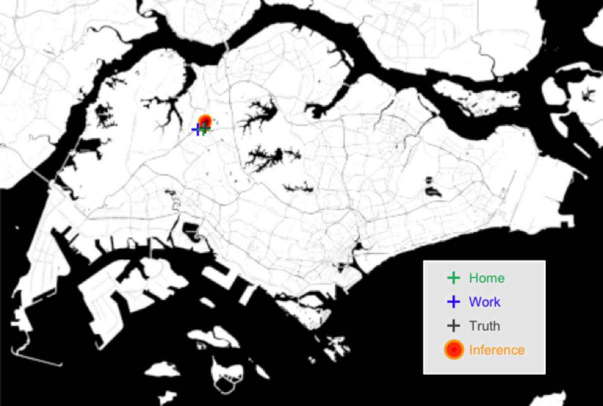
Home
Work
Truth
Inference

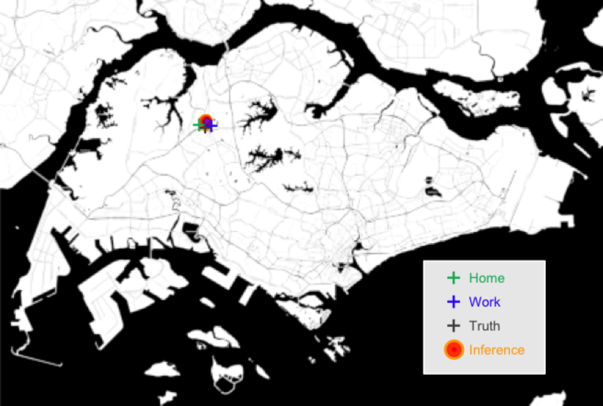
Home
Work
Truth
Inference

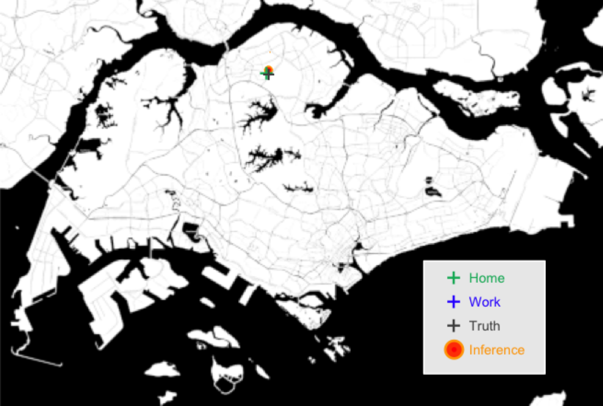
Home
Work
Truth
Inference

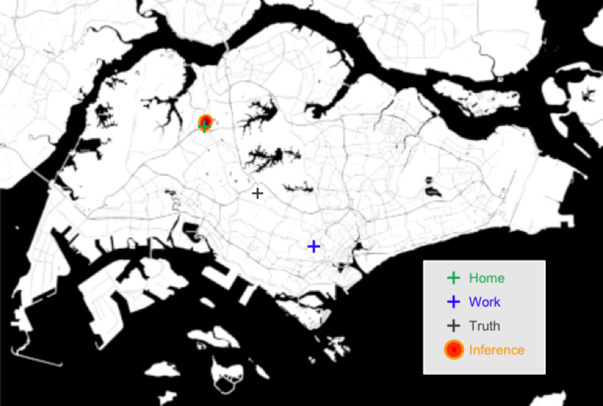
Home
Work
Truth
Inference

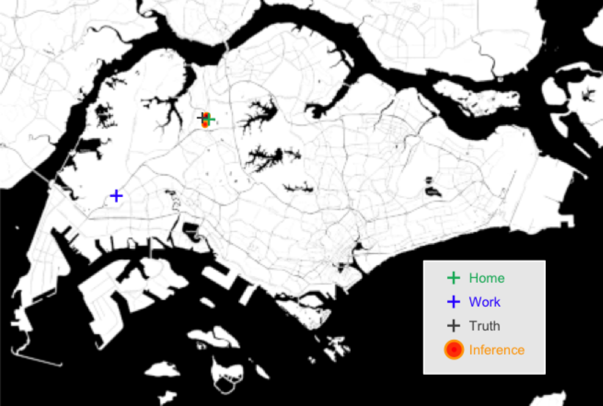
Home
Work
Truth
Inference

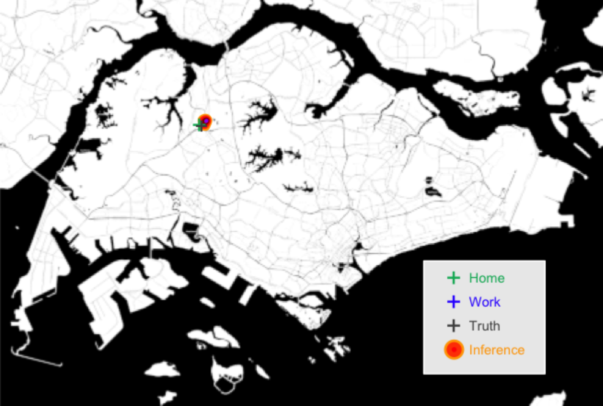
Home
Work
Truth
Inference

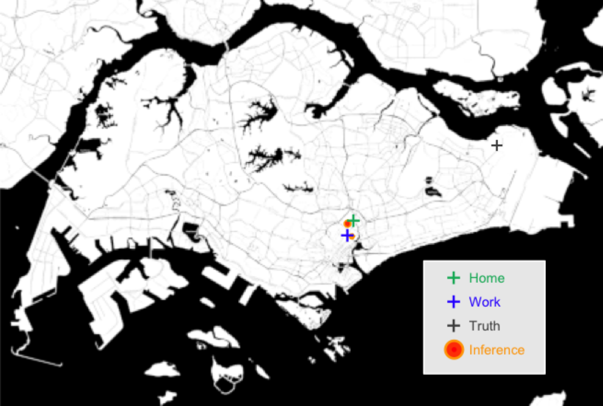

Home
Work
Truth
Inference

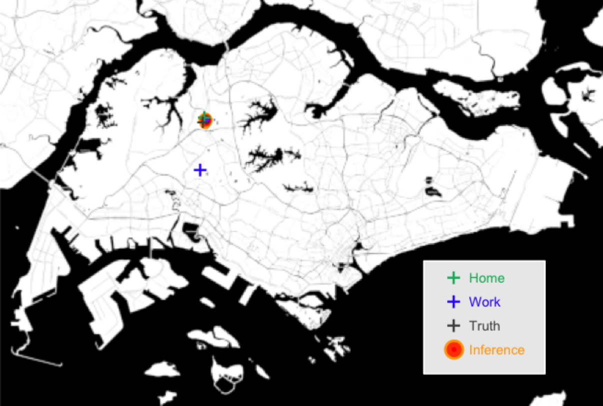

Home
Work
Truth
Inference

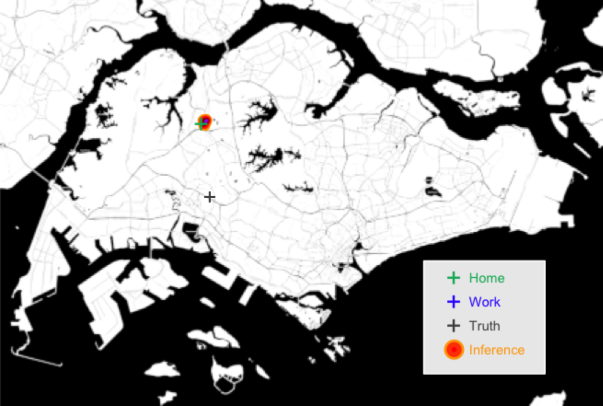
Home
Work
Truth
Inference

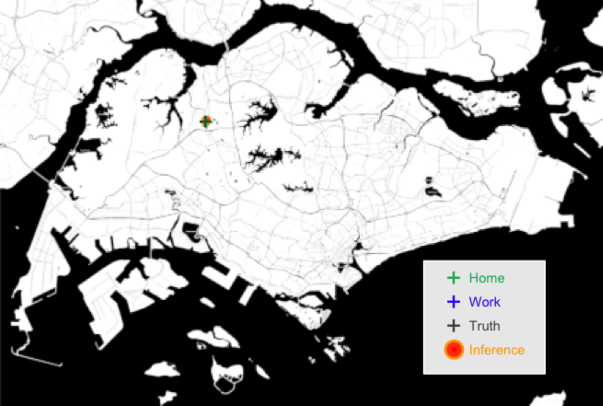

Home
Work
Truth
Inference

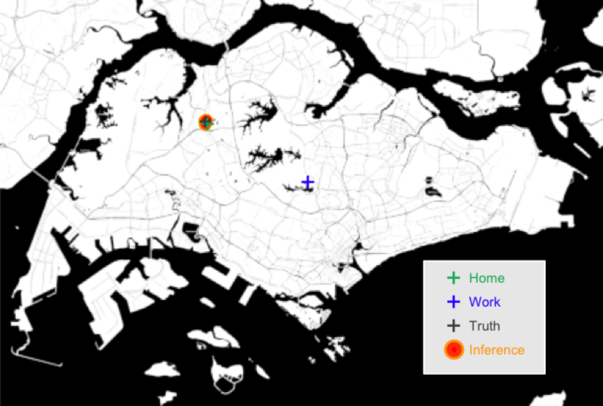
Home
Work
Truth
Inference

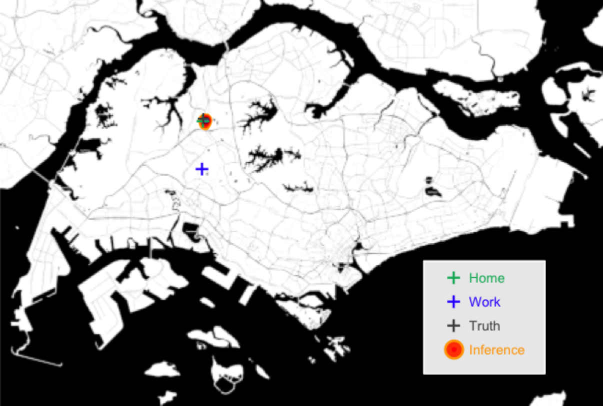
Home
Work
Truth
Inference

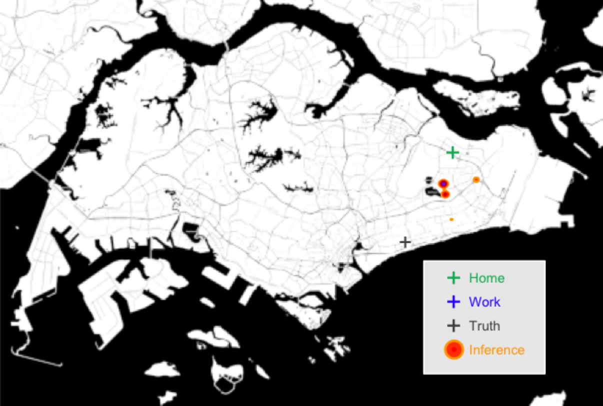

Home
Work
Truth
Inference

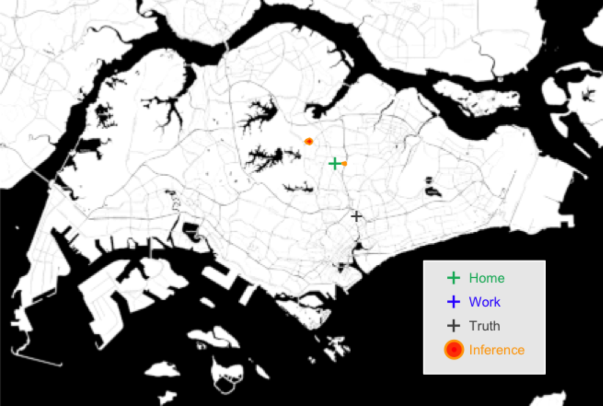

Home
Work
Truth
Inference

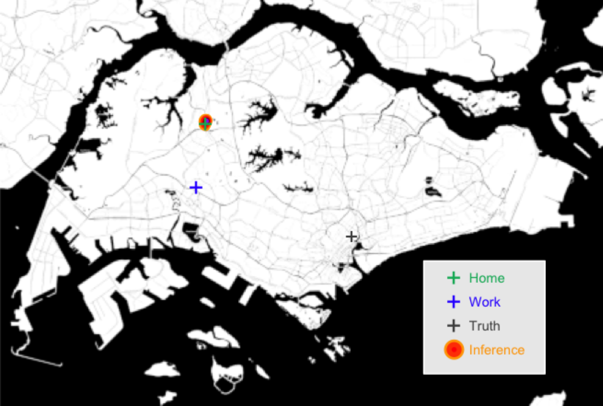

Home
Work
Truth
Inference

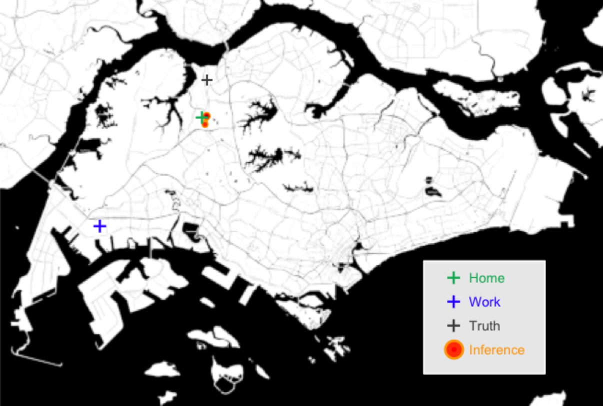
Home
Work
Truth
Inference

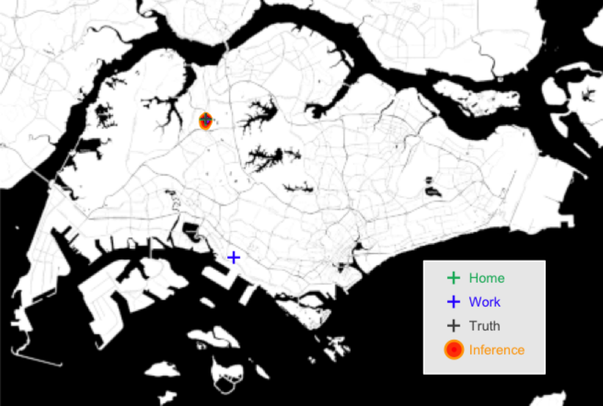
Home
Work
Truth
Inference

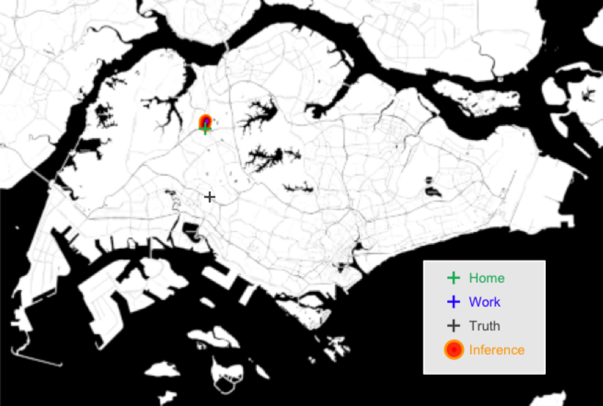
Home
Work
Truth
Inference

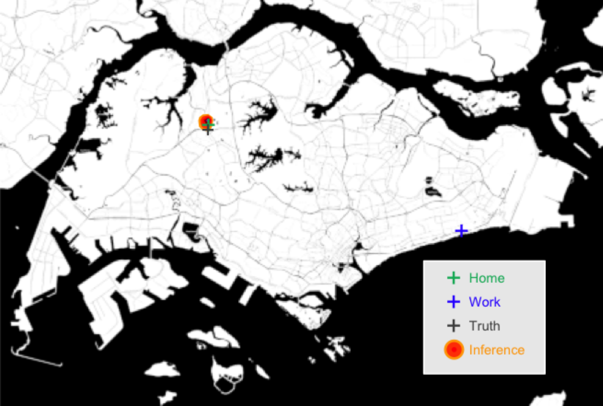
Home
Work
Truth
Inference

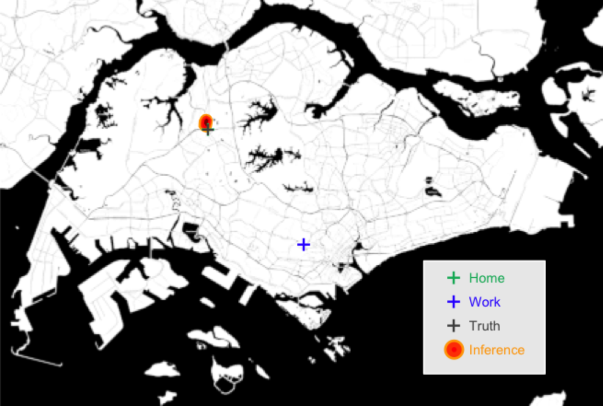
Home
Work
Truth
Inference

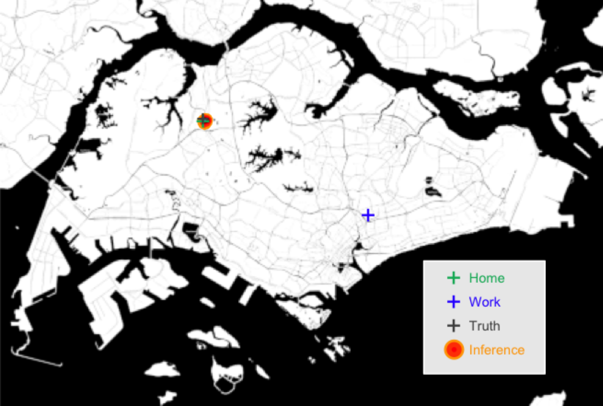
Home
Work
Truth
Inference

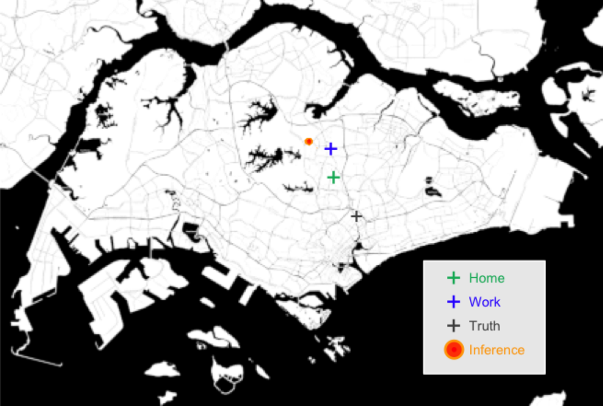

Home
Work
Truth
Inference

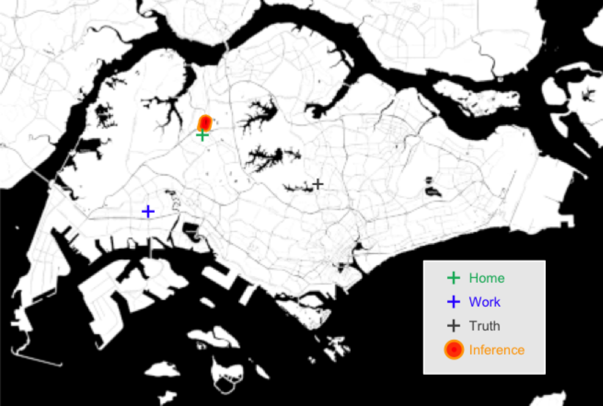
Home
Work
Truth
Inference

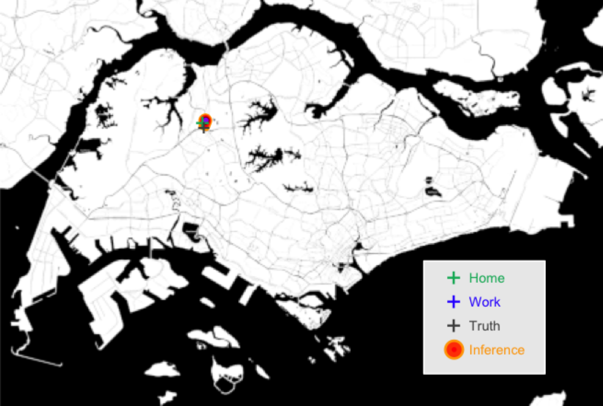
Home
Work
Truth
Inference

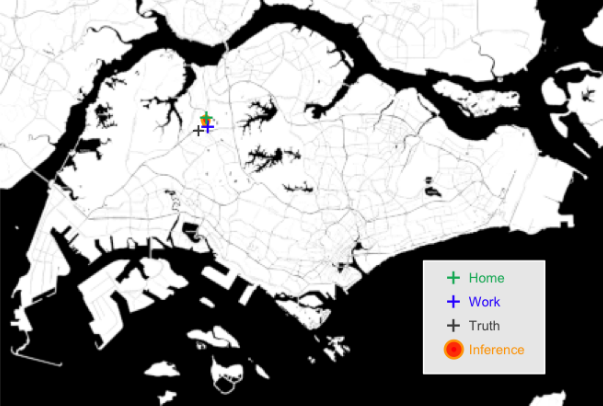
Home
Work
Truth
Inference

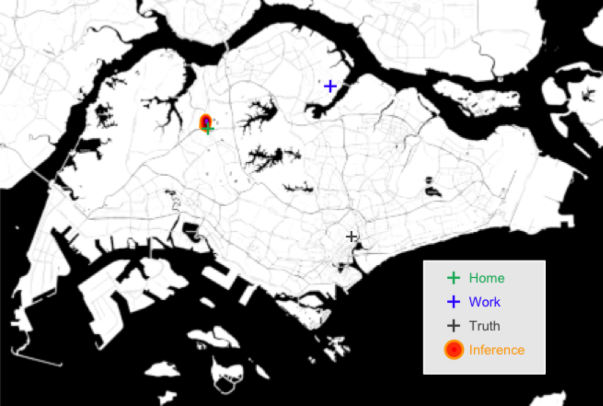
Home
Work
Truth
Inference

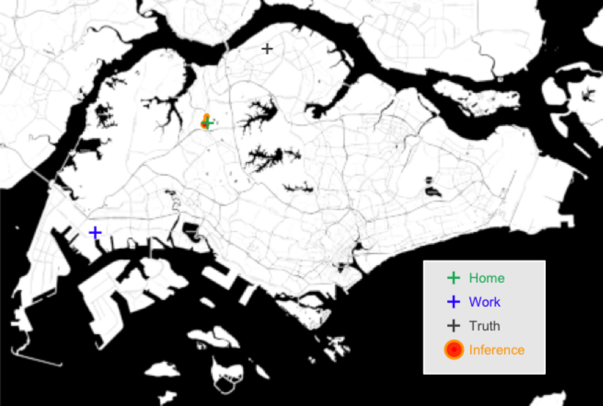
Home
Work
Truth
Inference

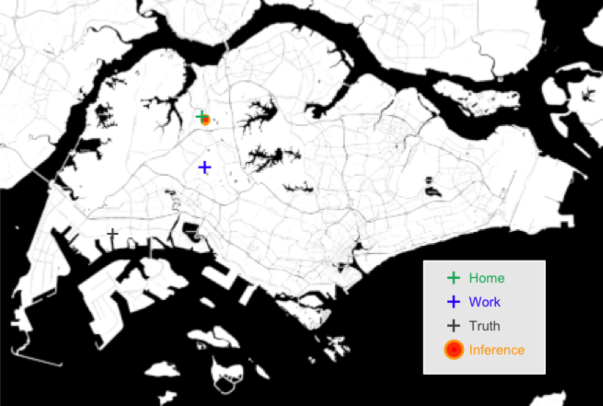
Home
Work
Truth
Inference

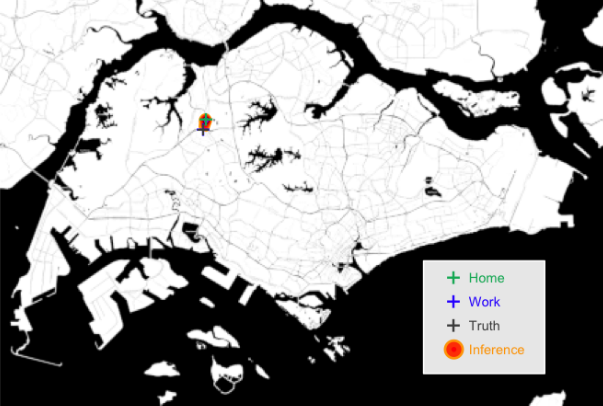
Home
Work
Truth
Inference

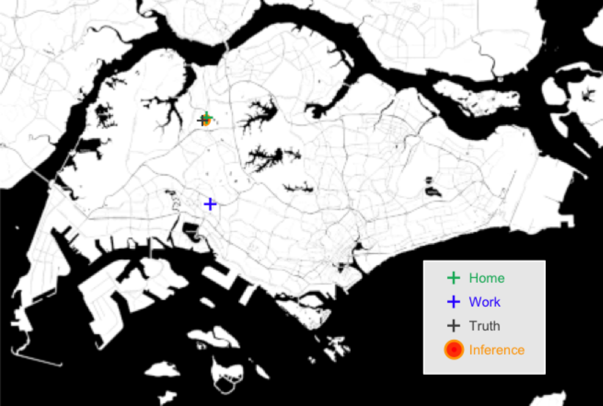
Home
Work
Truth
Inference

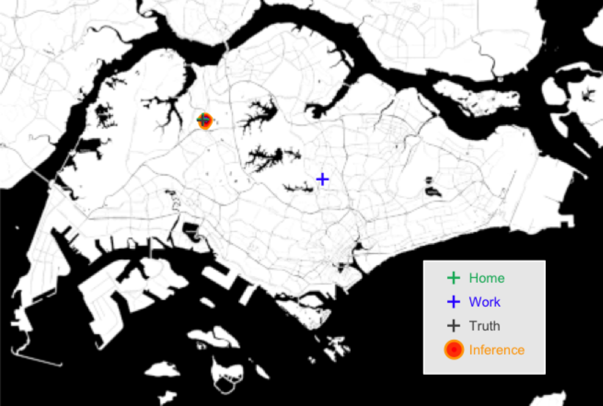
Home
Work
Truth
Inference

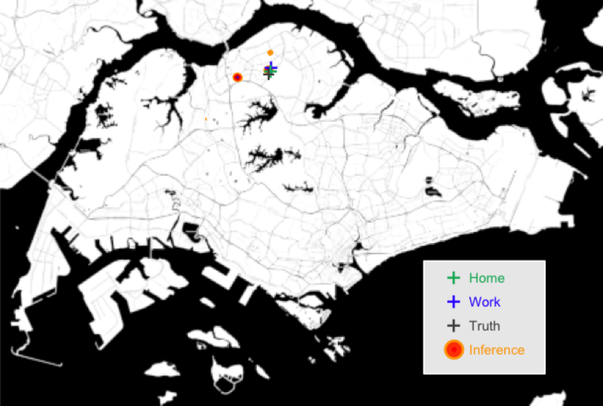
Home
Work
Truth
Inference

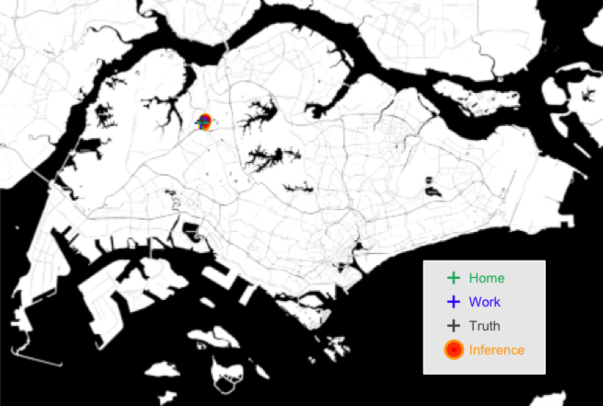
Home
Work
Truth
Inference

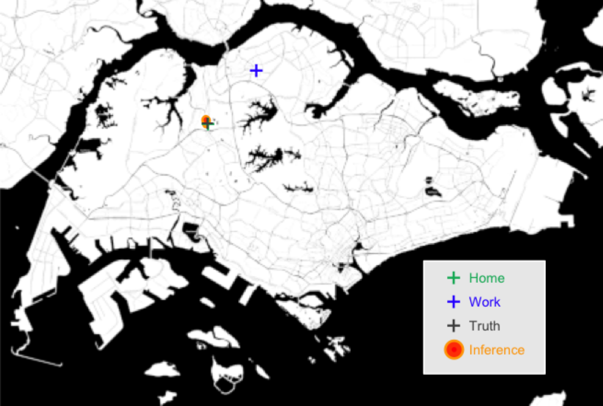

Home
Work
Truth
Inference

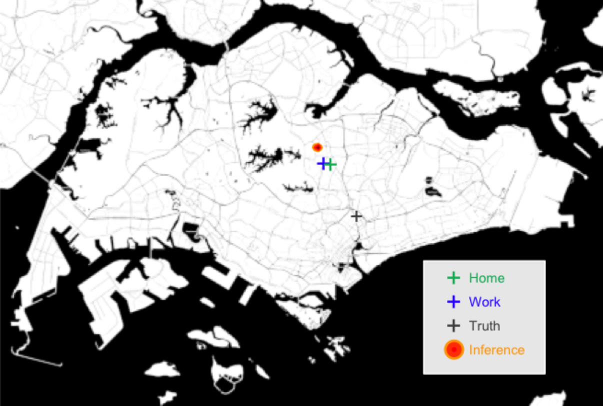

Home
Work
Truth
Inference

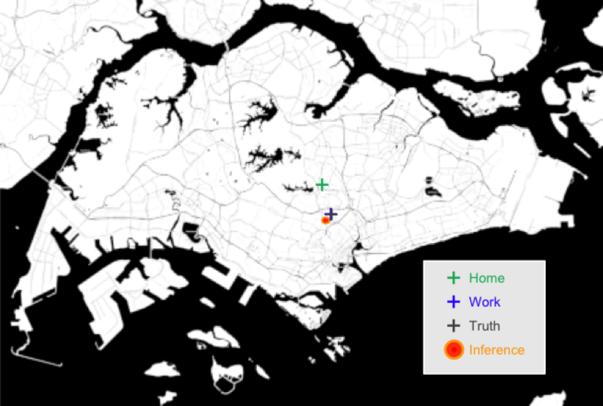
Home
Work
Truth
Inference

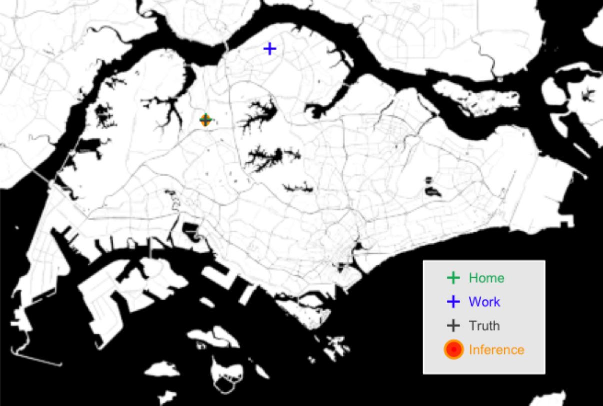
Home
Work
Truth
Inference

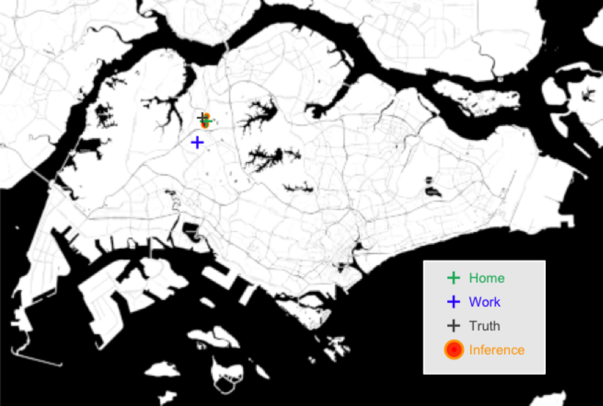

Home
Work
Truth
Inference

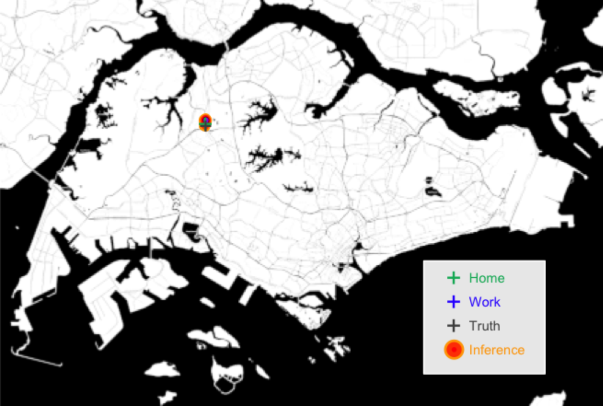
Home
Work
Truth
Inference

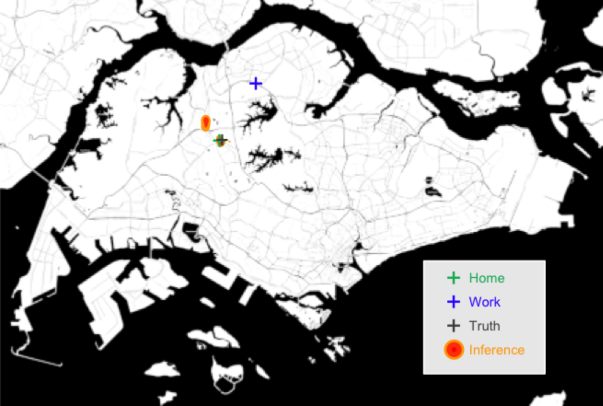
Home
Work
Truth
Inference

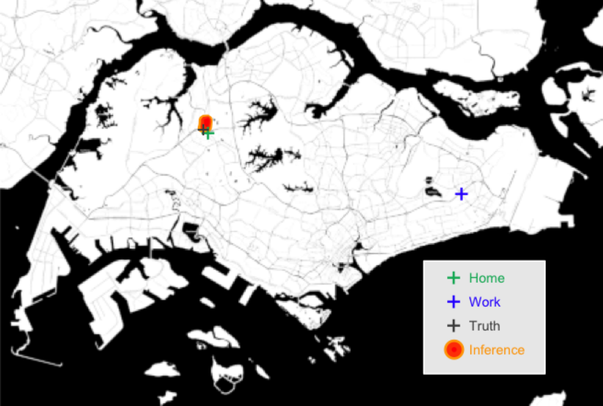
Home
Work
Truth
Inference

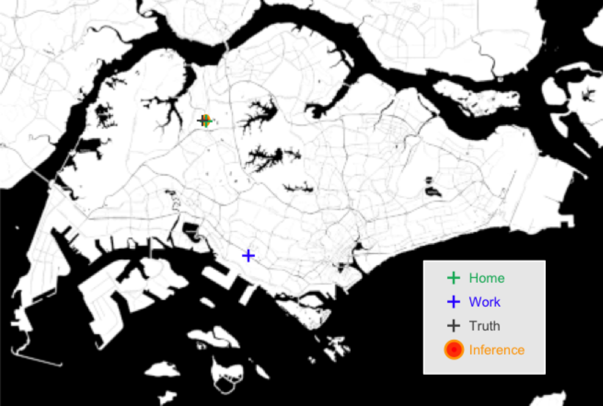
Home
Work
Truth
Inference

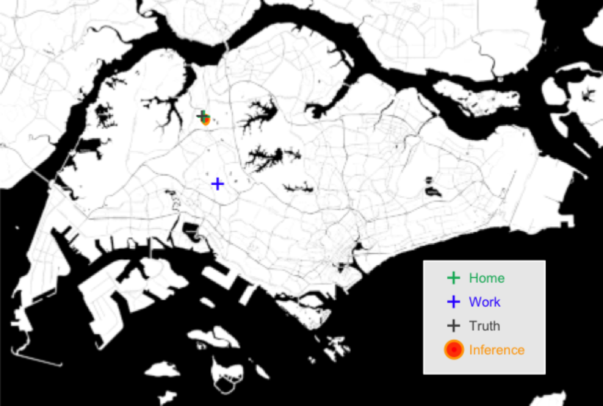
Home
Work
Truth
Inference

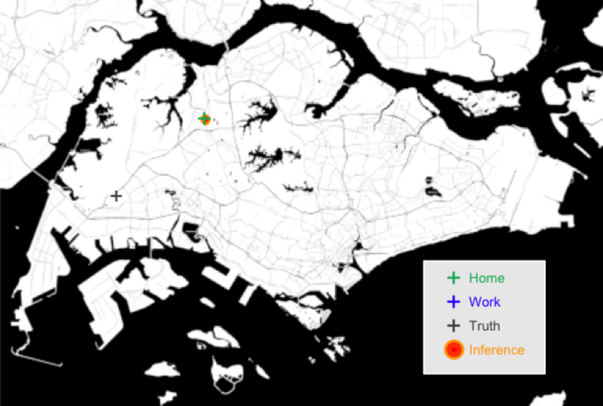

Home
Work
Truth
Inference

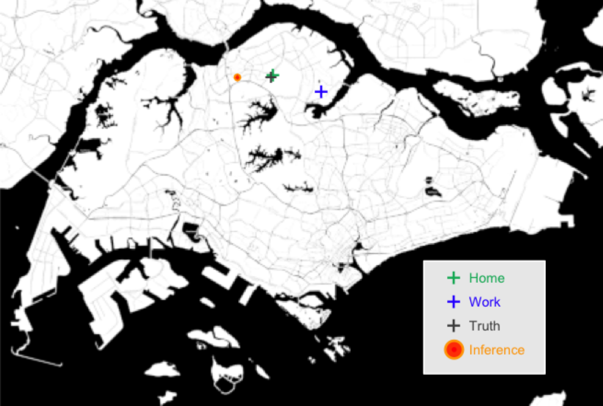

Home
Work
Truth
Inference

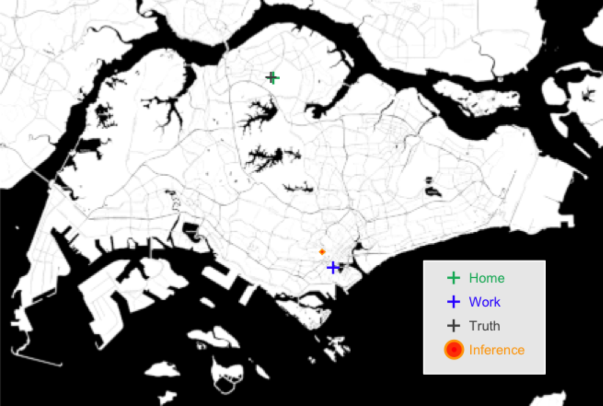

Home
Work
Truth
Inference

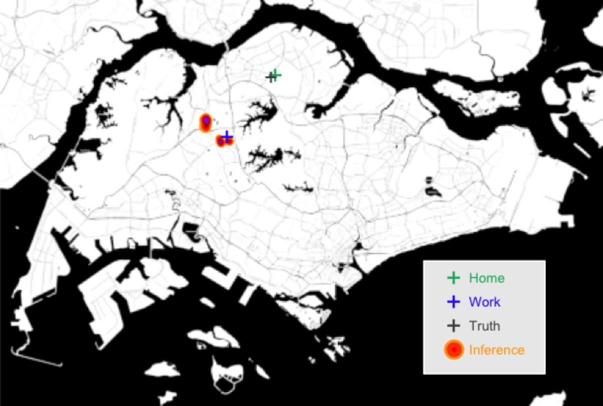
Home
Work
Truth
Inference

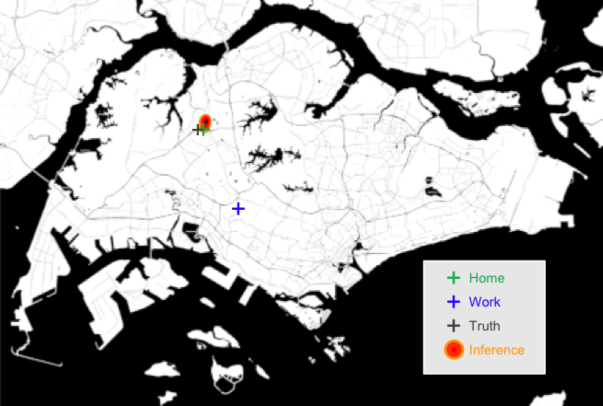
Home
Work
Truth
Inference

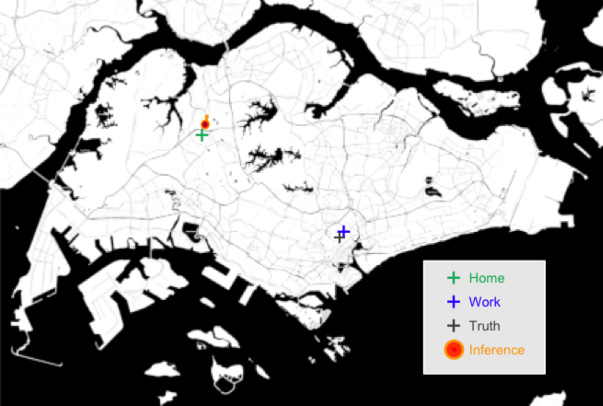
Home
Work
Truth
Inference

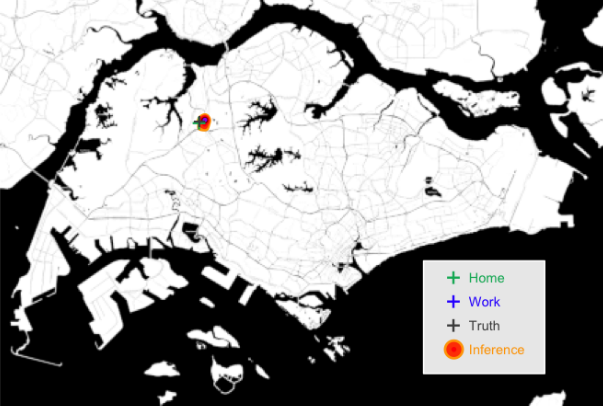
Home
Work
Truth
Inference

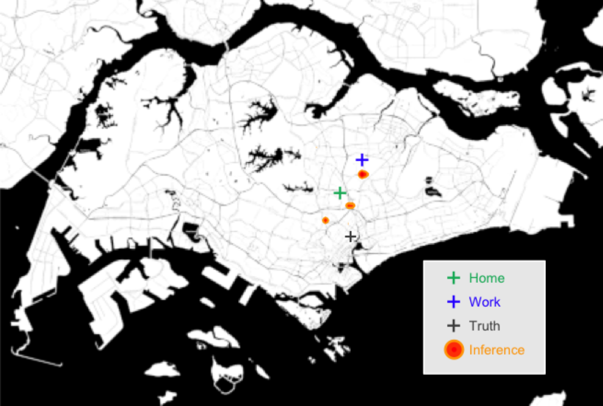
Home
Work
Truth
Inference

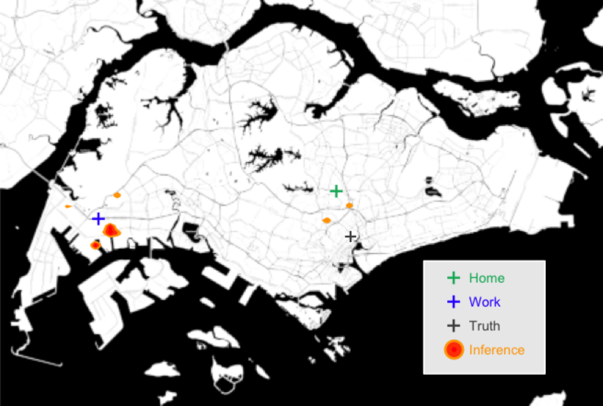
Home
Work
Truth
Inference

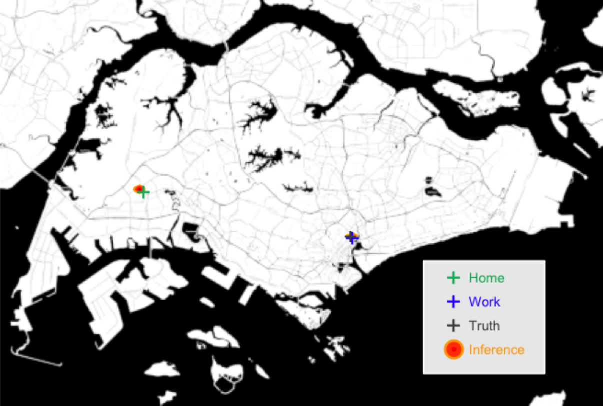
Home
Work
Truth
Inference

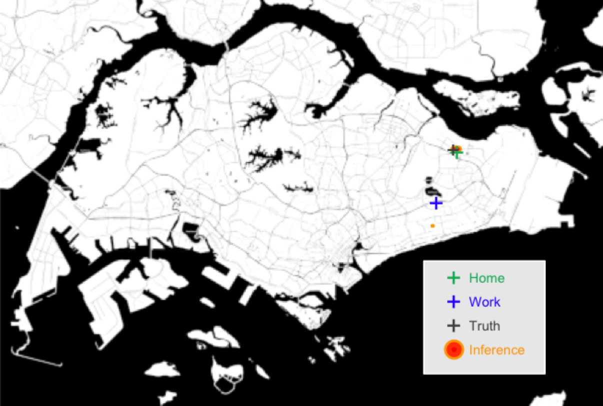
Home
Work
Truth
Inference

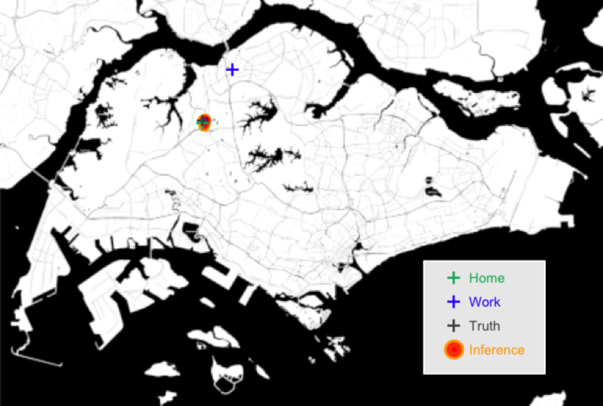
Home
Work
Truth
Inference

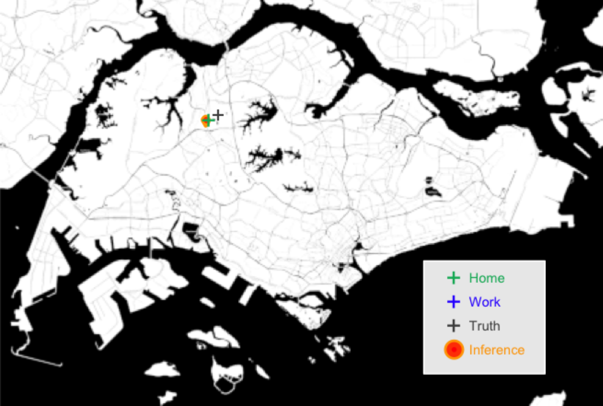
Home
Work
Truth
Inference

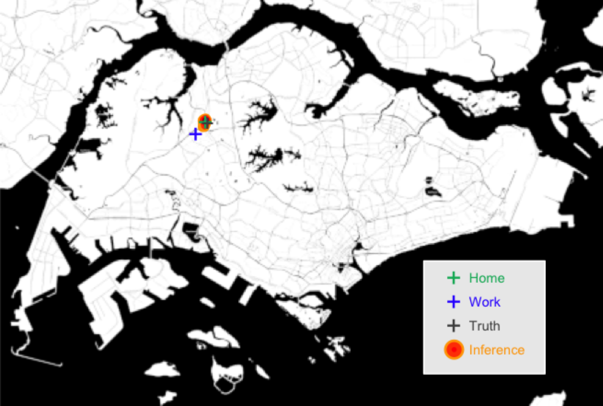
Home
Work
Truth
Inference

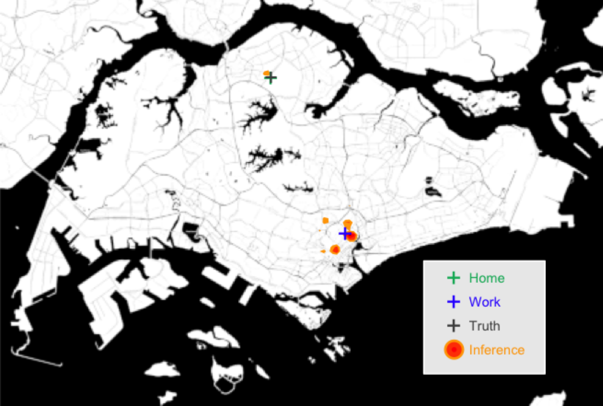
Home
Work
Truth
Inference

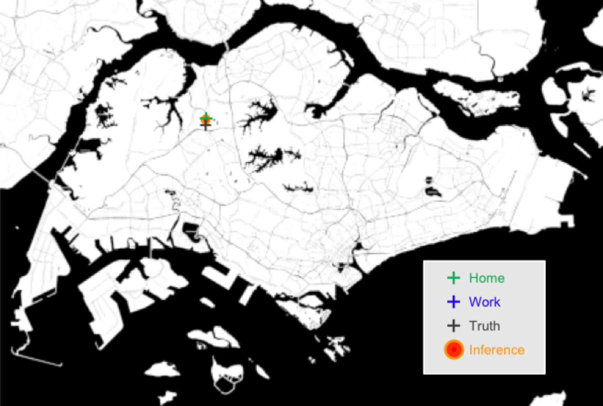
Home
Work
Truth
Inference

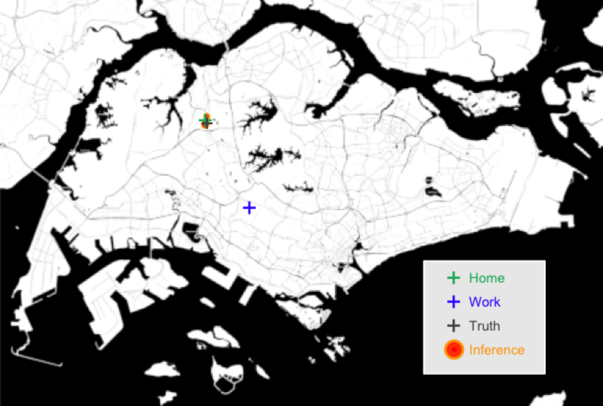
Home
Work
Truth
Inference

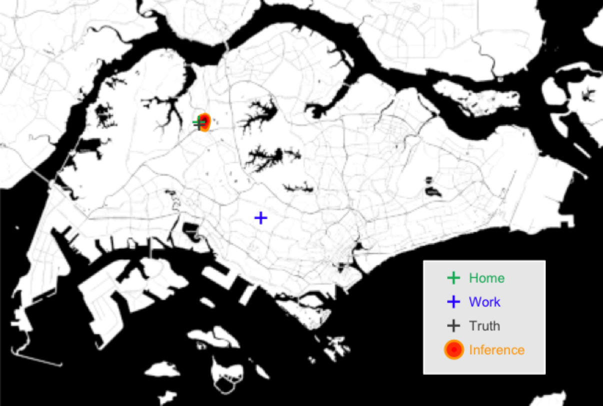
Home
Work
Truth
Inference

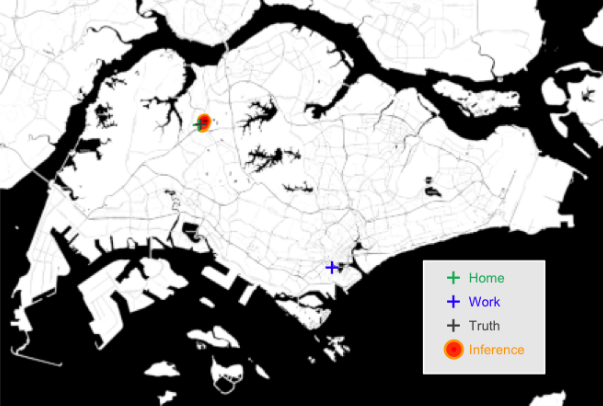
Home
Work
Truth
Inference

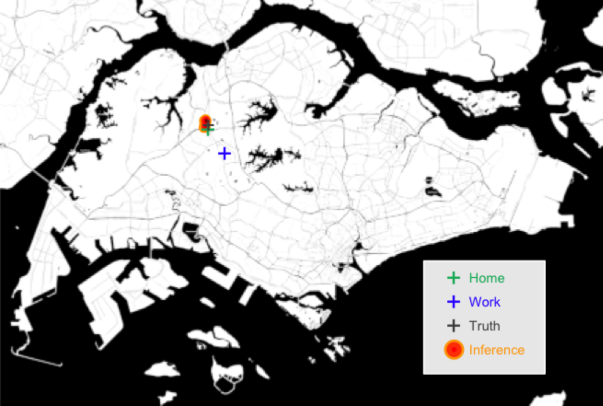

Home
Work
Truth
Inference

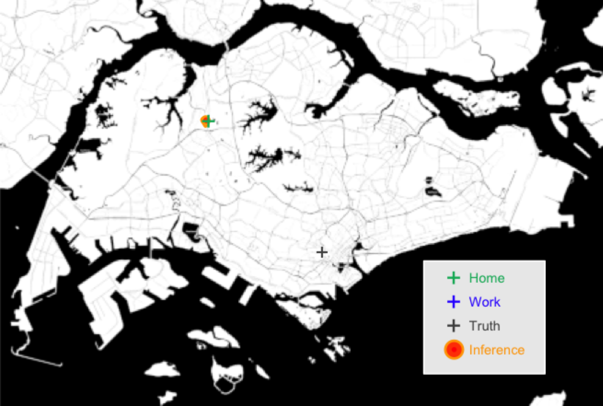

Home
Work
Truth
Inference

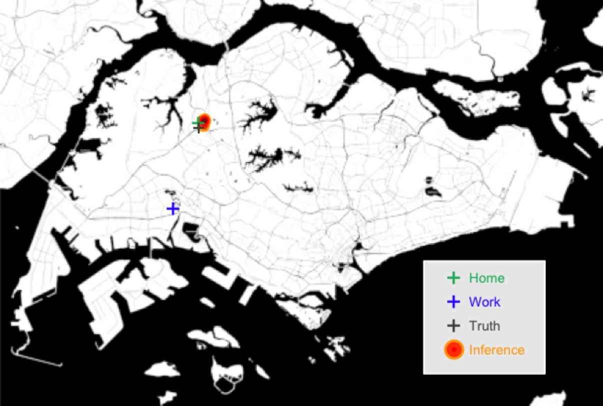
Home
Work
Truth
Inference

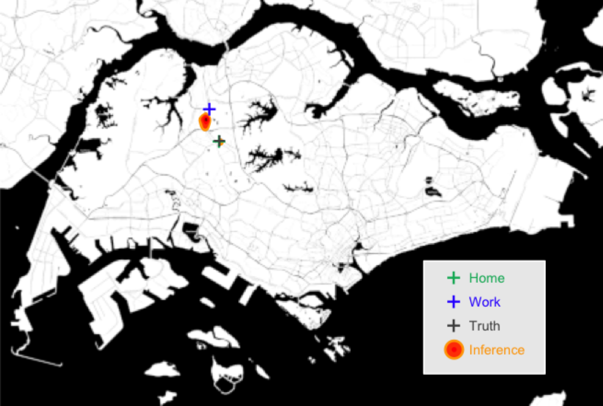
Home
Work
Truth
Inference

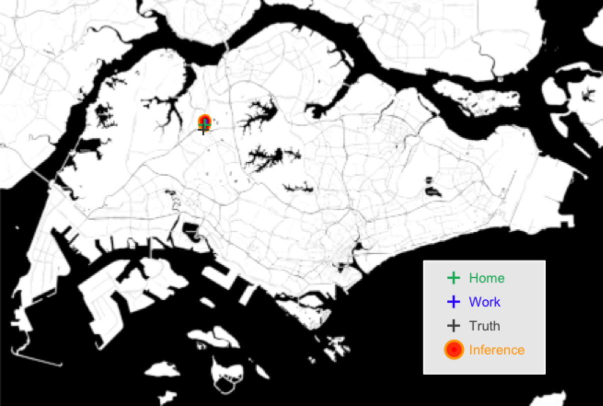

Home
Work
Truth
Inference

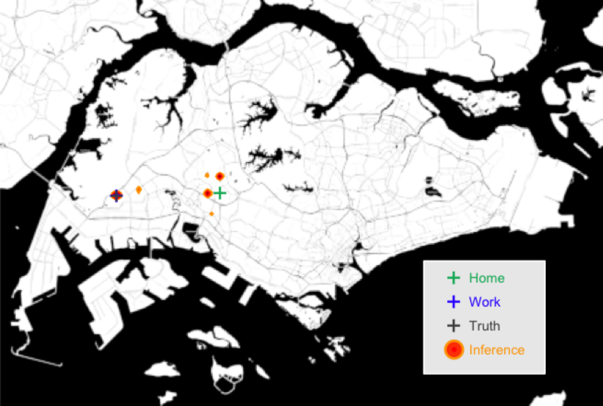
Home
Work
Truth
Inference

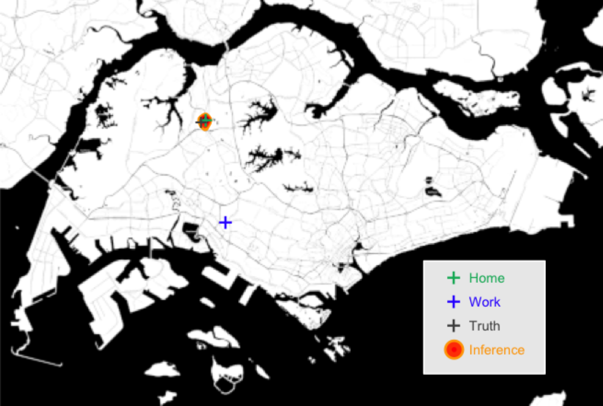
Home
Work
Truth
Inference

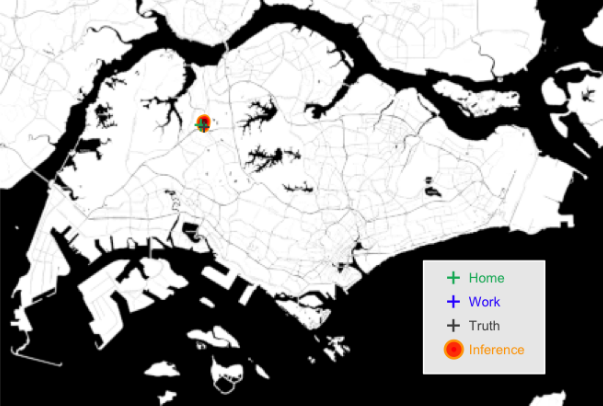
Home
Work
Truth
Inference

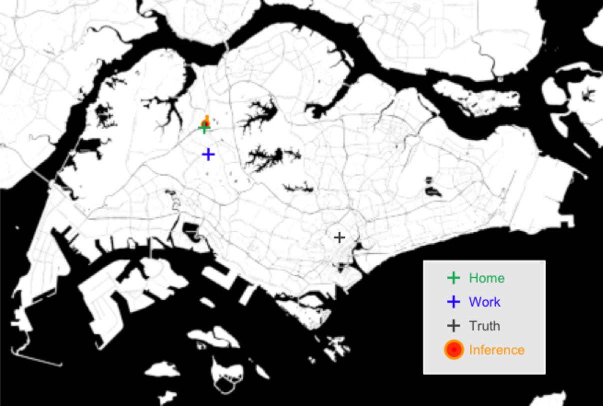
Home
Work
Truth
Inference

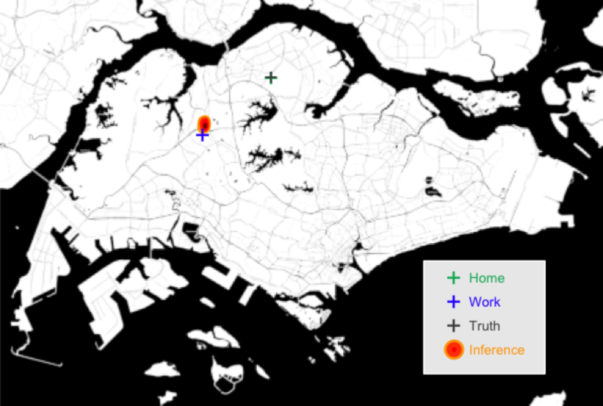
Home
Work
Truth
Inference

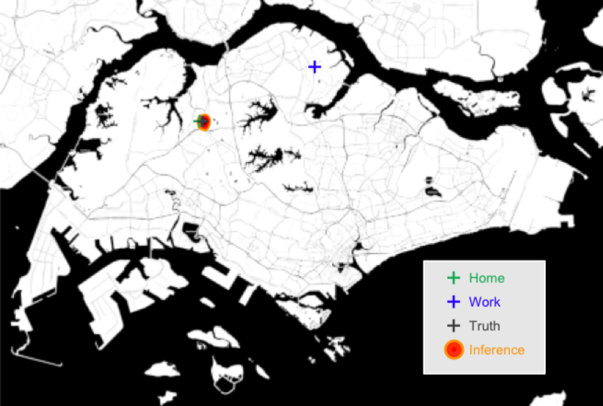

Home
Work
Truth
Inference

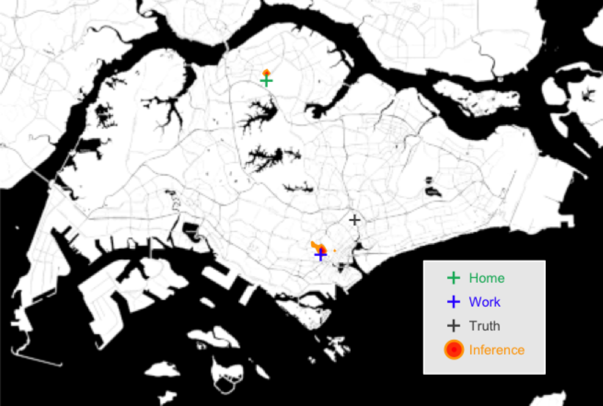
Home
Work
Truth
Inference

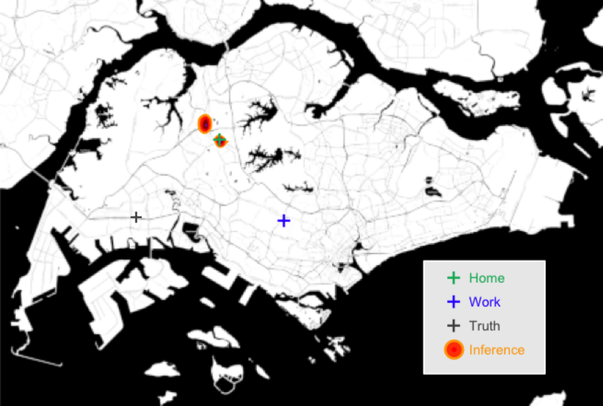

Home
Work
Truth
Inference

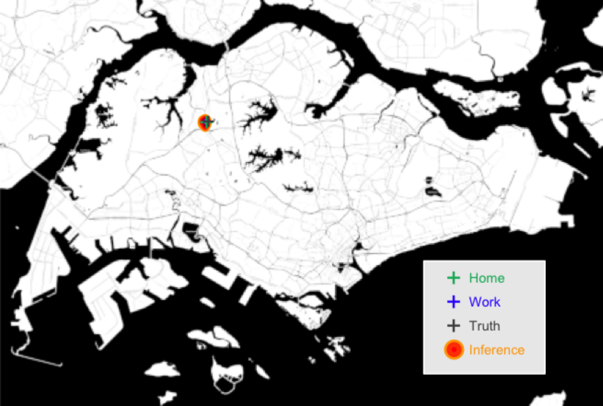
Home
Work
Truth
Inference

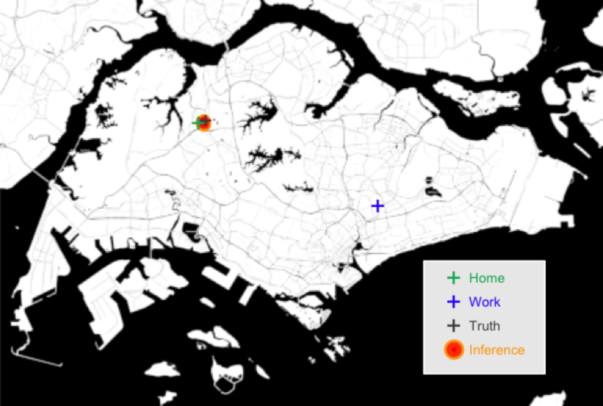
Home
Work
Truth
Inference

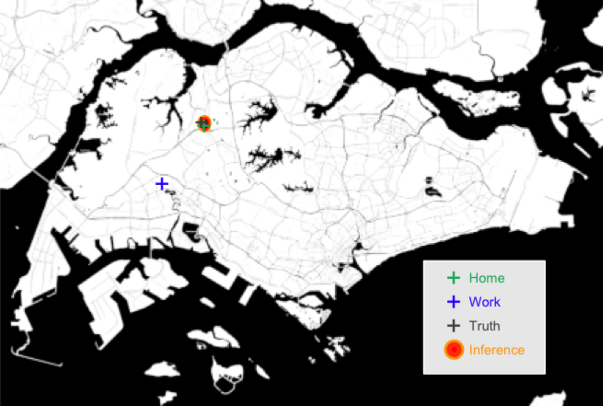
Home
Work
Truth
Inference

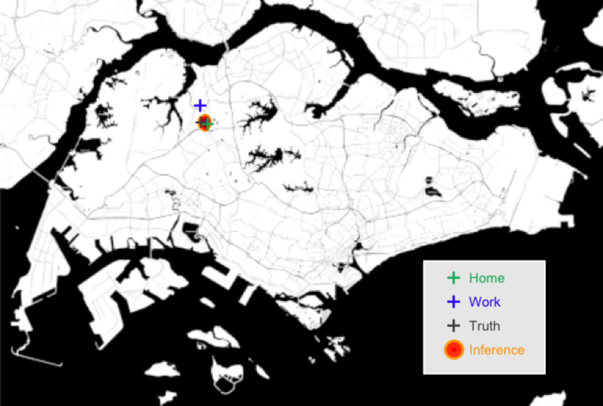
Home
Work
Truth
Inference

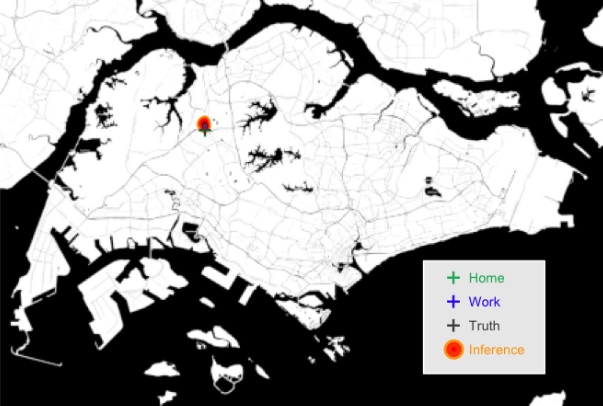
Home
Work
Truth
Inference

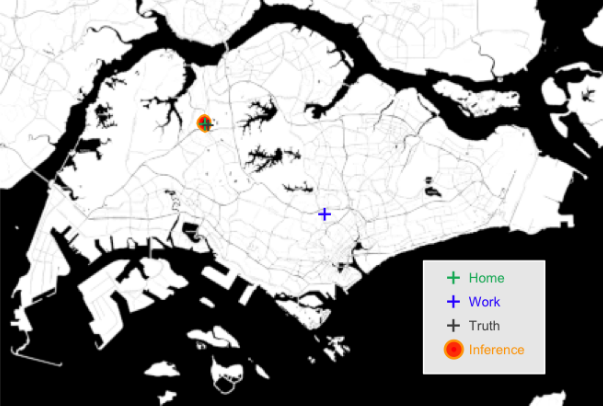
Home
Work
Truth
Inference

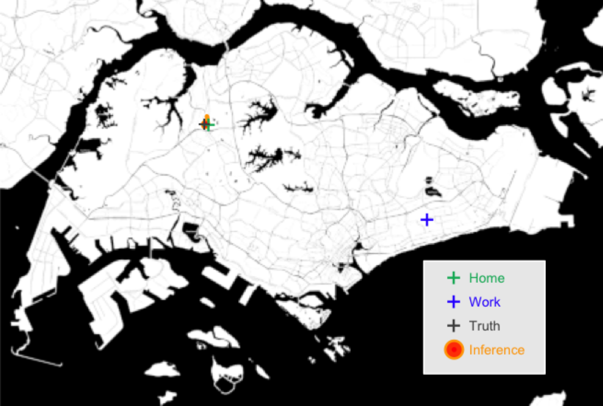
Home
Work
Truth
Inference

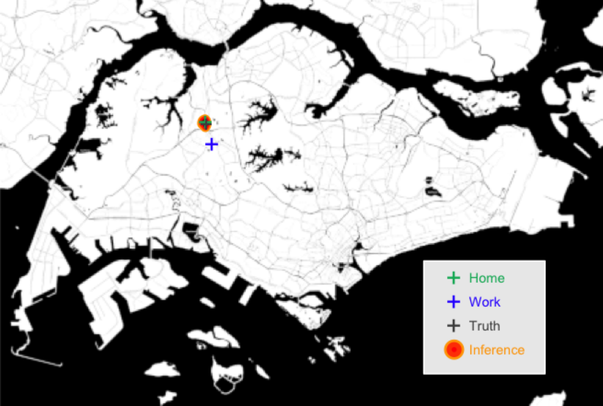
Home
Work
Truth
Inference

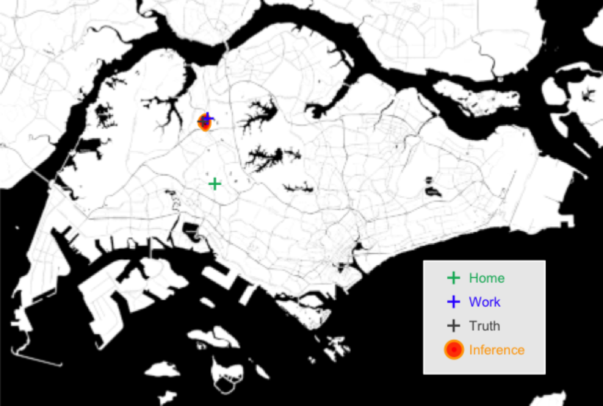
Home
Work
Truth
Inference

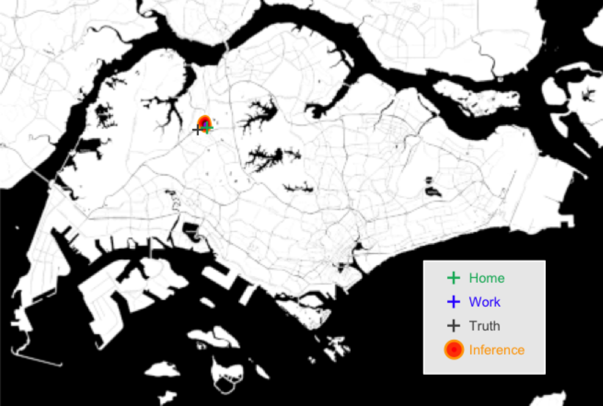
Home
Work
Truth
Inference

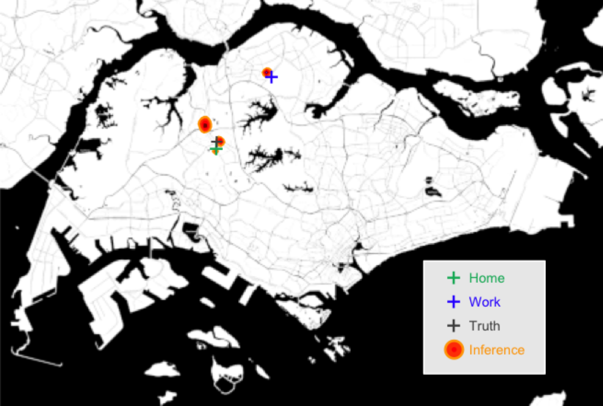
Home
Work
Truth
Inference

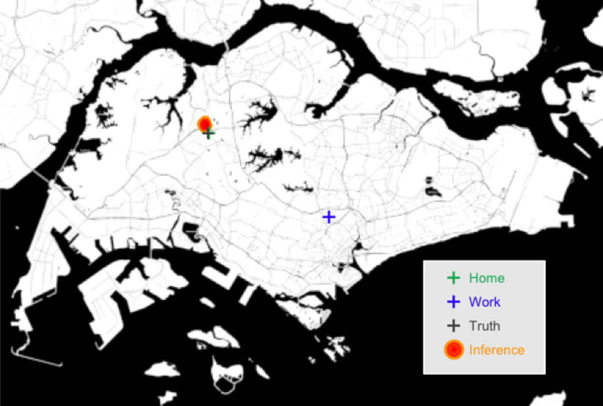
Home
Work
Truth
Inference

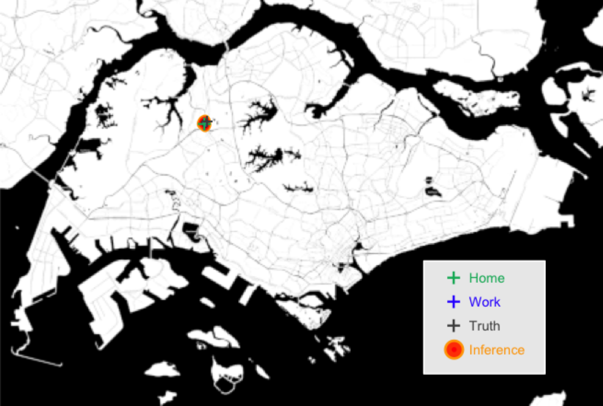
Home
Work
Truth
Inference

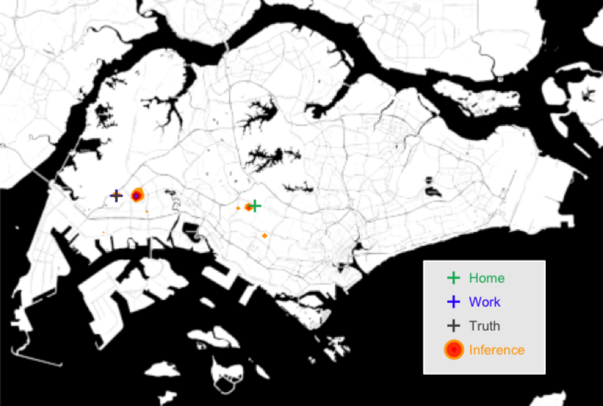
Home
Work
Truth
Inference

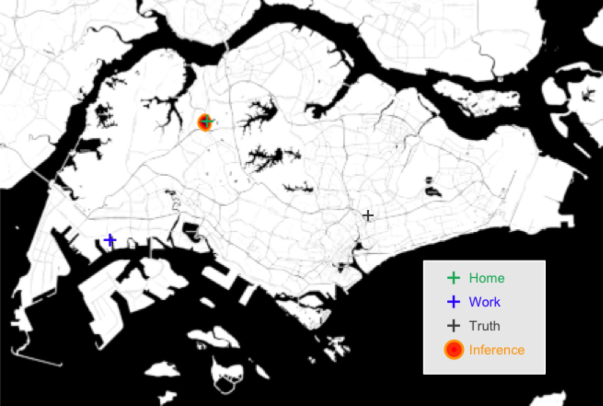

Home
Work
Truth
Inference

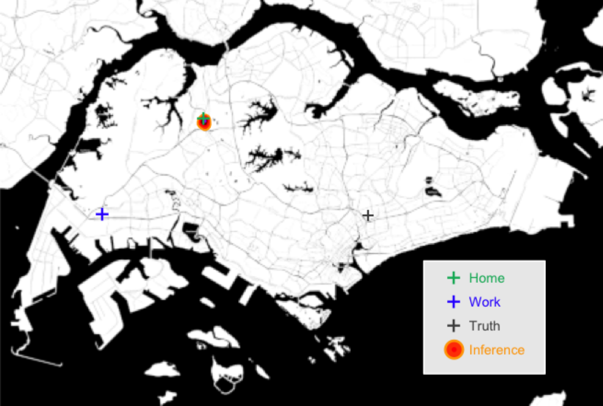
Home
Work
Truth
Inference

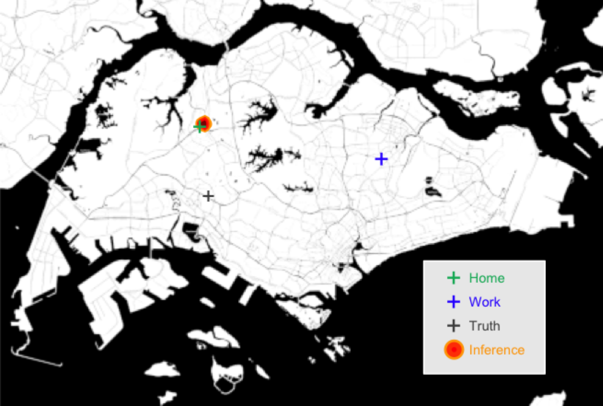

Home
Work
Truth
Inference

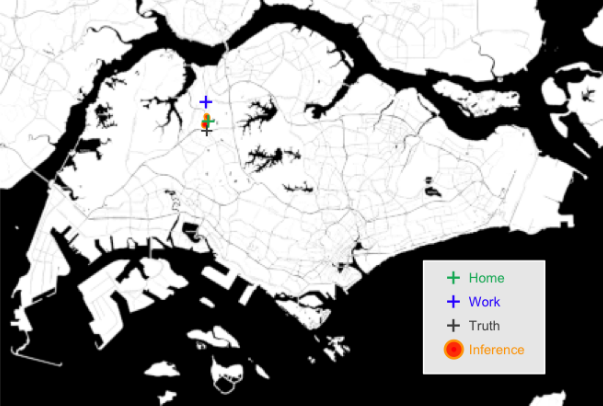
Home
Work
Truth
Inference

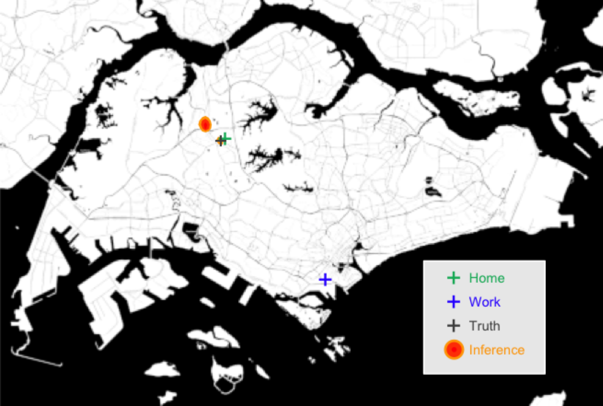
Home
Work
Truth
Inference

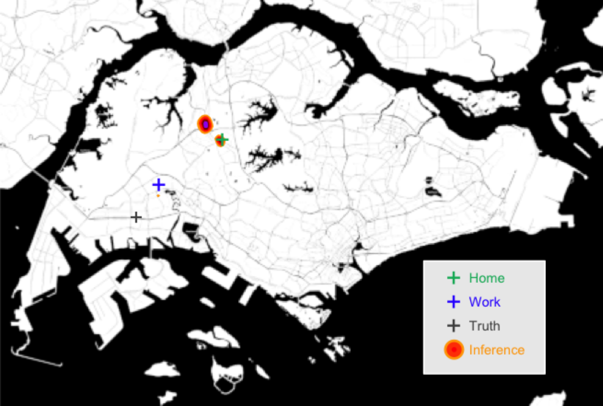
Home
Work
Truth
Inference

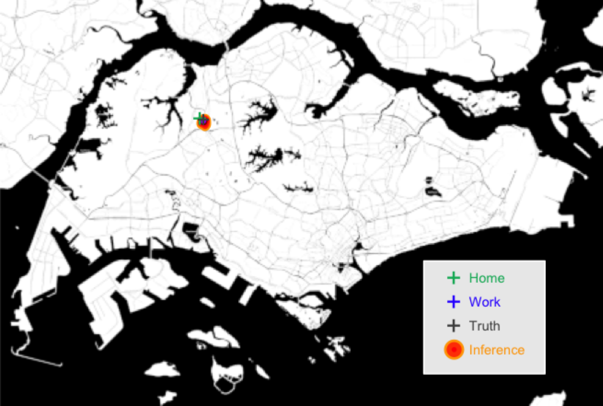
Home
Work
Truth
Inference

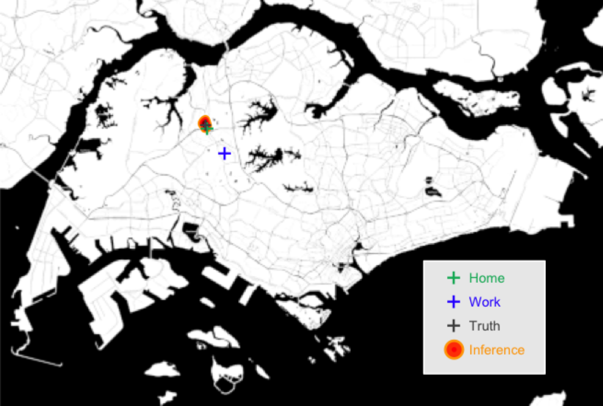
Home
Work
Truth
Inference

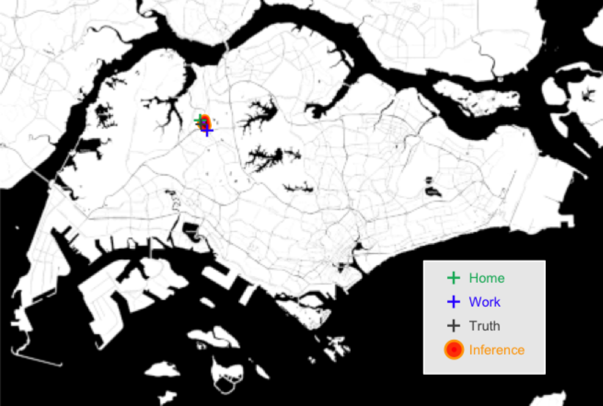
Home
Work
Truth
Inference

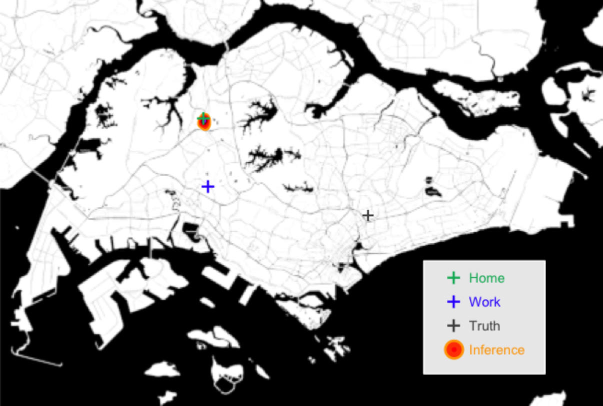
Home
Work
Truth
Inference

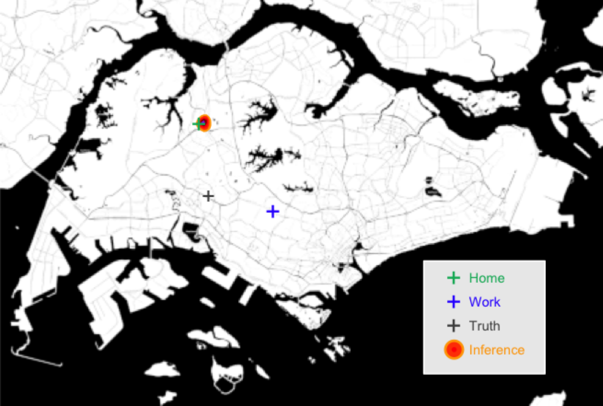
Home
Work
Truth
Inference

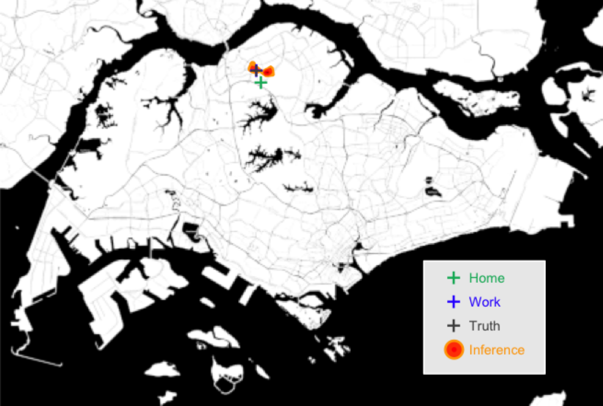
Home
Work
Truth
Inference

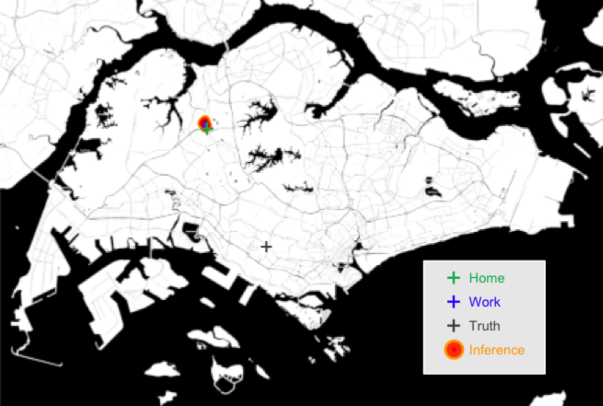
Home
Work
Truth
Inference

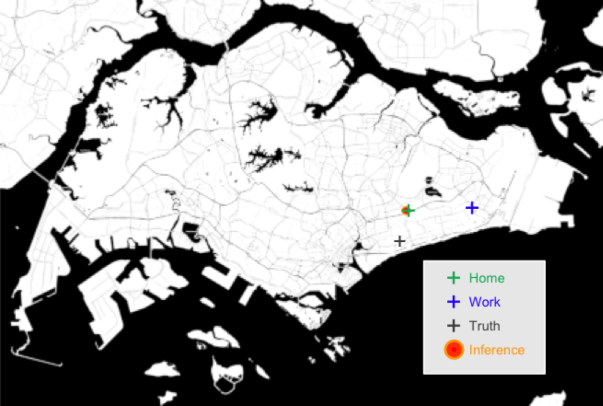
Home
Work
Truth
Inference

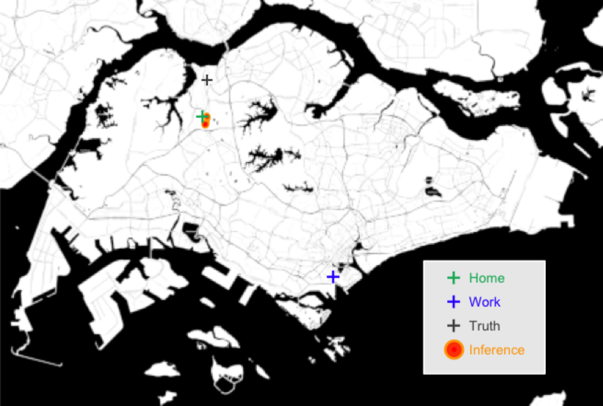
Home
Work
Truth
Inference

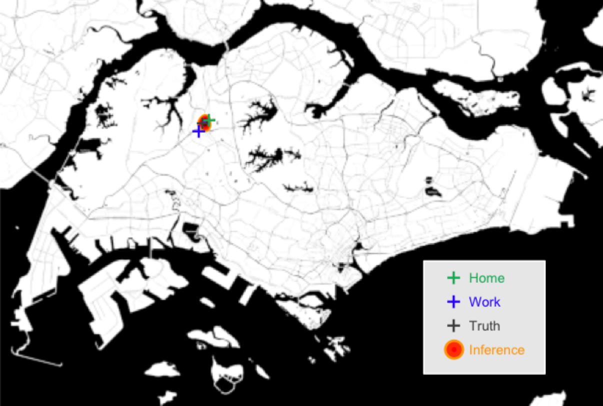
Home
Work
Truth
Inference

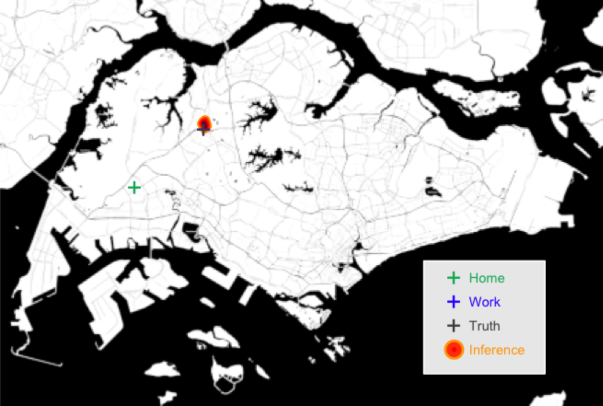
Home
Work
Truth
Inference

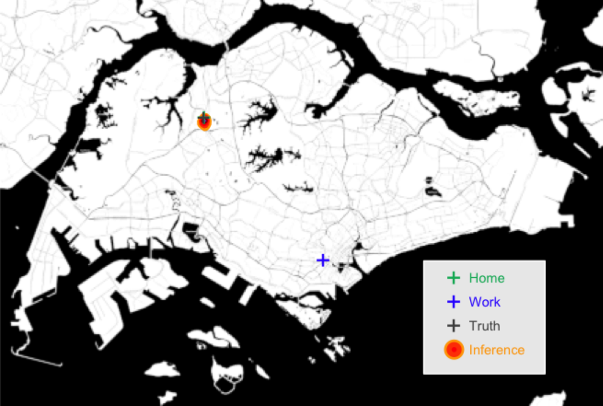

Home
Work
Truth
Inference

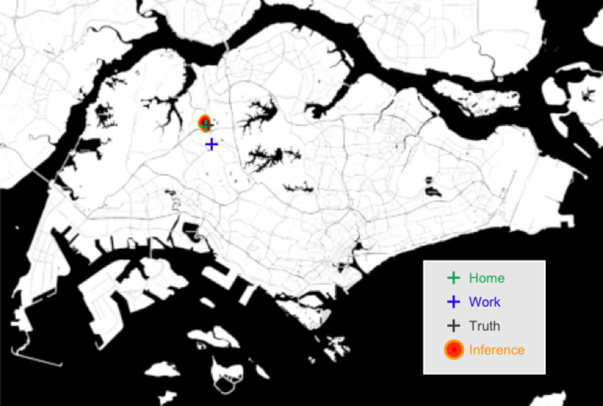

Home
Work
Truth
Inference

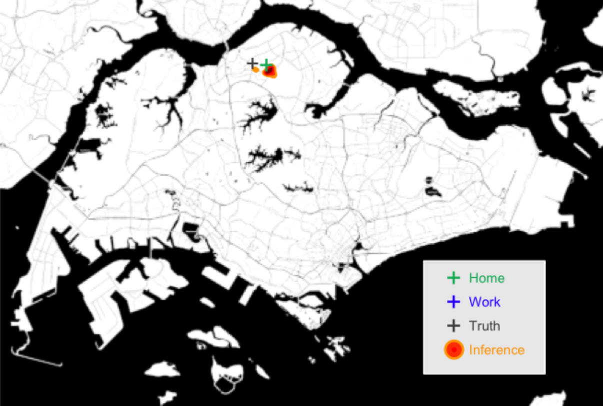
Home
Work
Truth
Inference

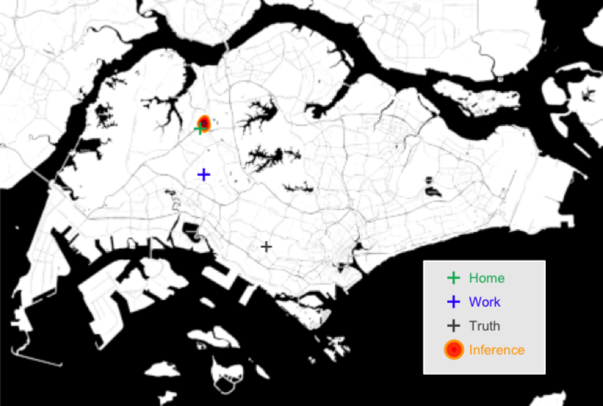

Home
Work
Truth
Inference

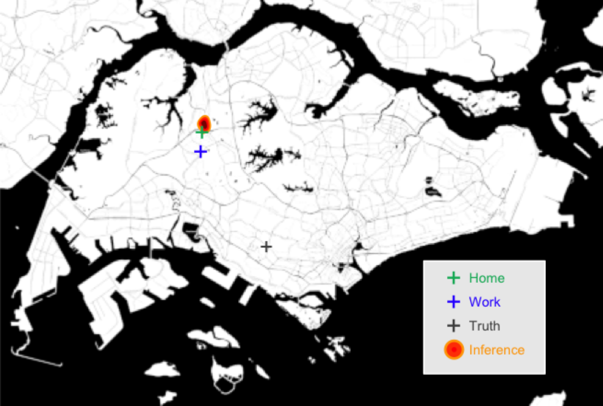

Home
Work
Truth
Inference

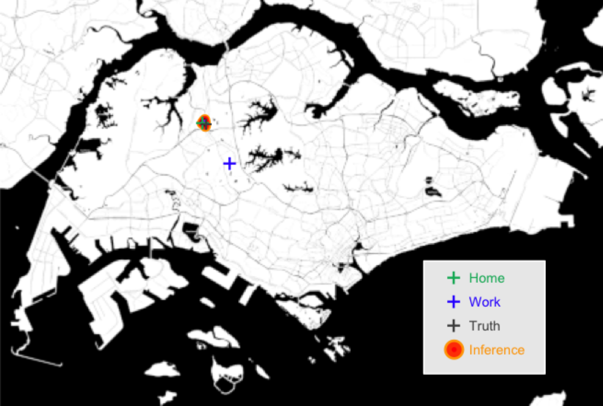
Home
Work
Truth
Inference

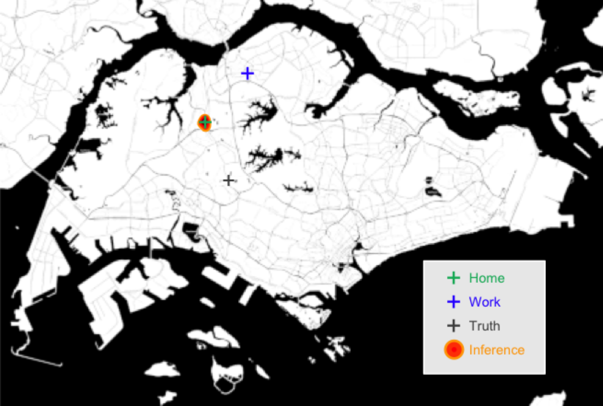

Home
Work
Truth
Inference

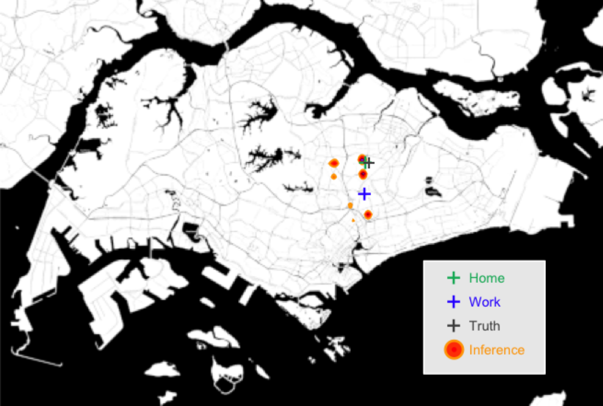
Home
Work
Truth
Inference

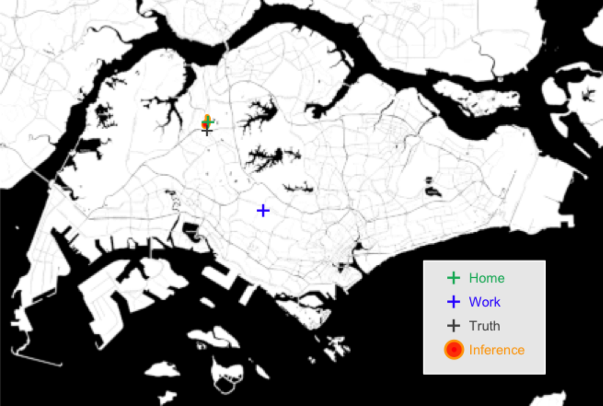
Home
Work
Truth
Inference

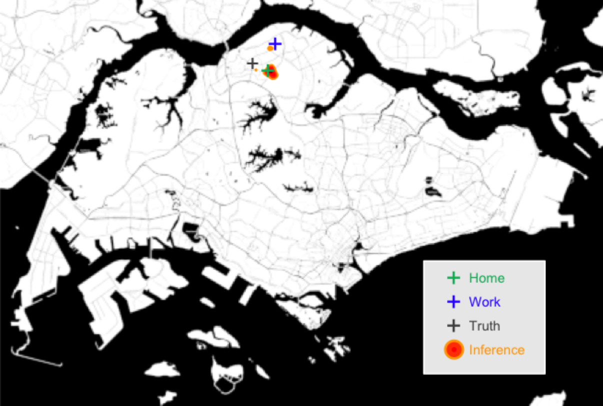
Home
Work
Truth
Inference

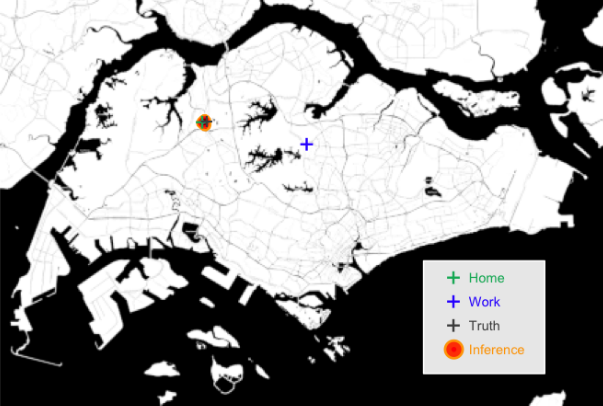
Home
Work
Truth
Inference

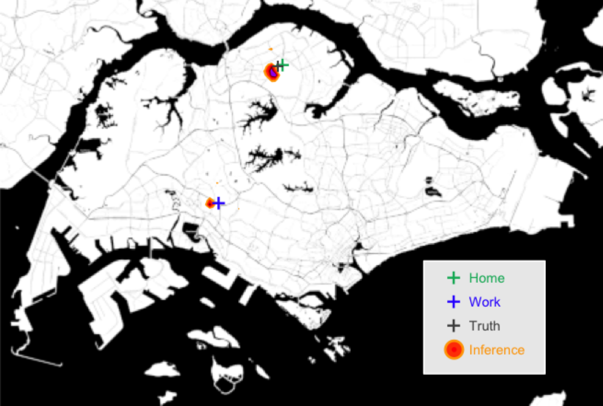
Home
Work
Truth
Inference

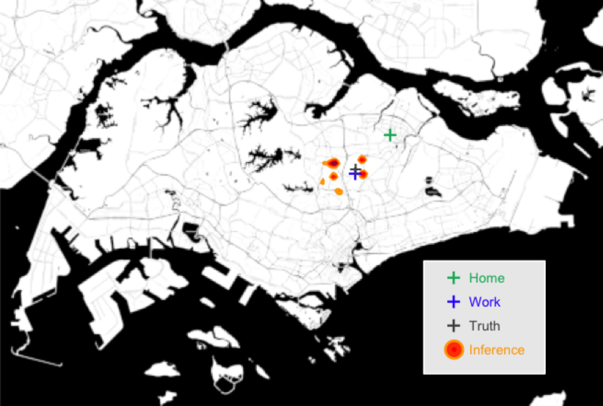

Home
Work
Truth
Inference

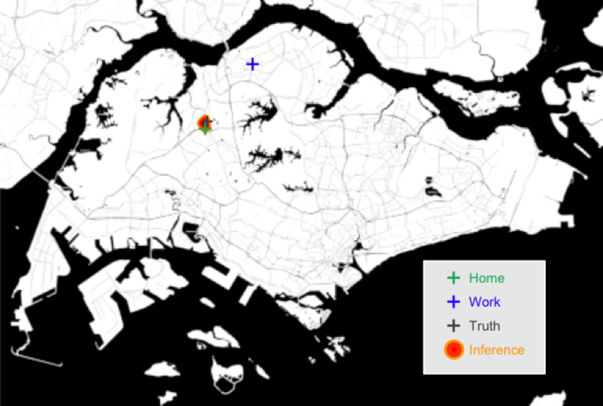
Home
Work
Truth
Inference

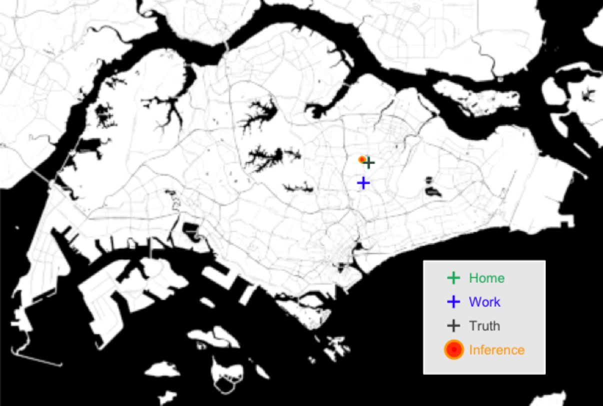
Home
Work
Truth
Inference

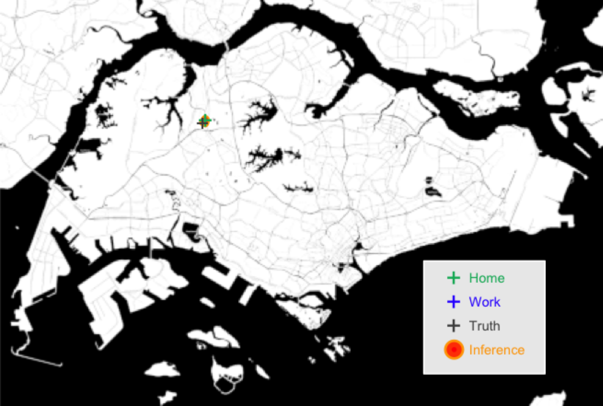

Home
Work
Truth
Inference

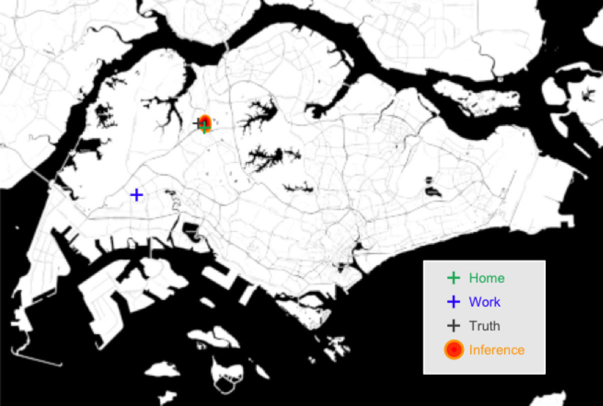
Home
Work
Truth
Inference

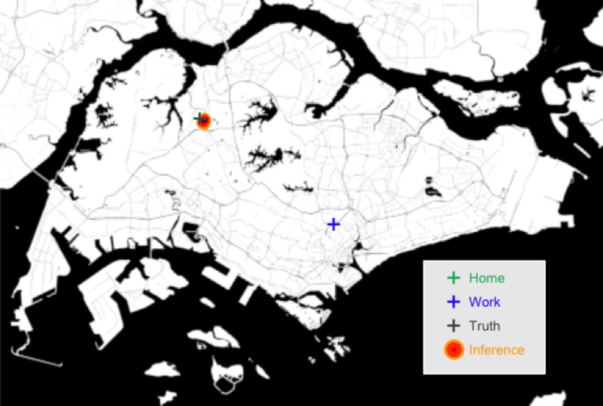
Home
Work
Truth
Inference

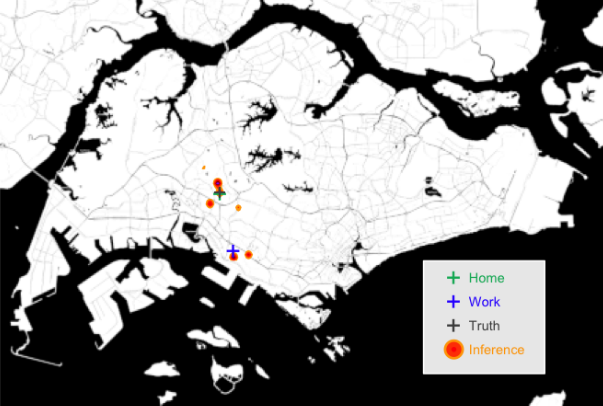
Home
Work
Truth
Inference

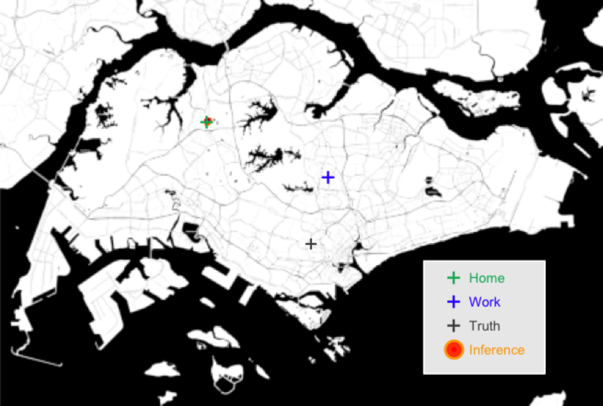
Home
Work
Truth
Inference

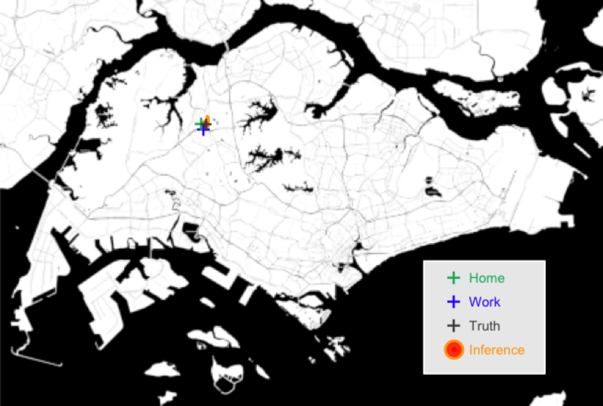
Home
Work
Truth
Inference

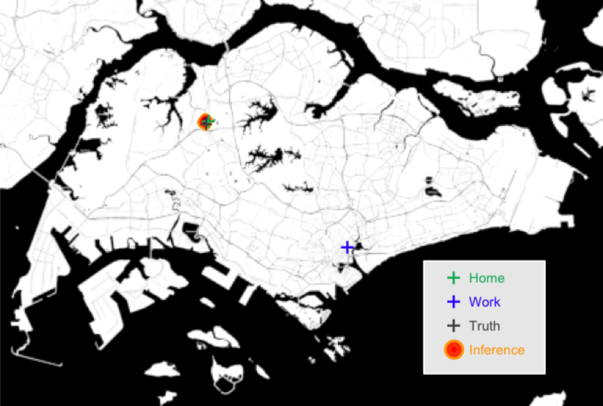
Home
Work
Truth
Inference

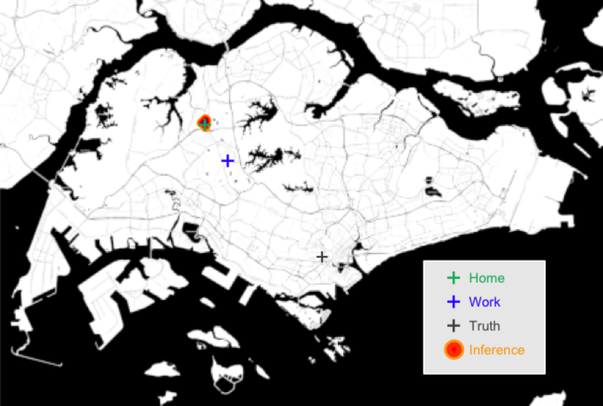
Home
Work
Truth
Inference

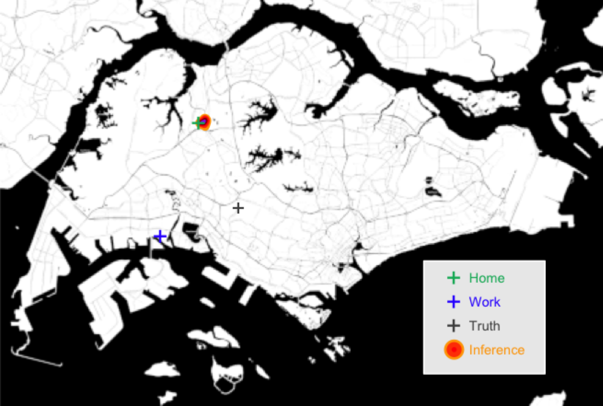
Home
Work
Truth
Inference

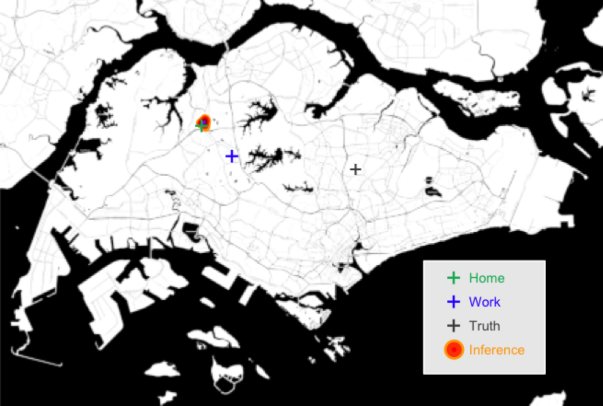
Home
Work
Truth
Inference

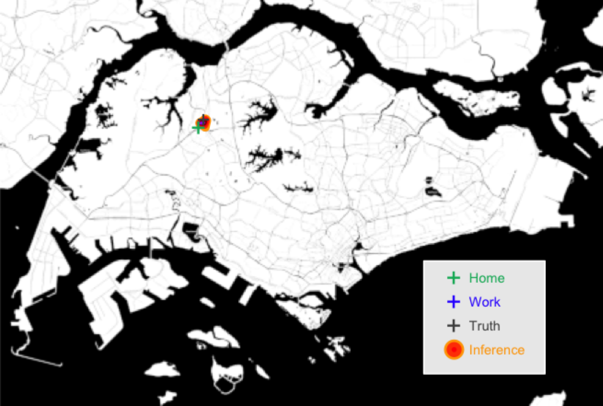
Home
Work
Truth
Inference

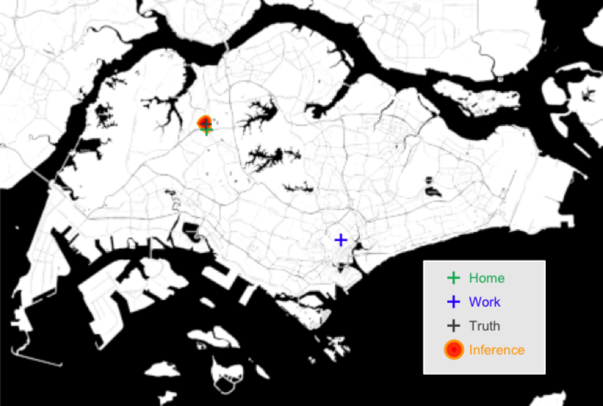
Home
Work
Truth
Inference

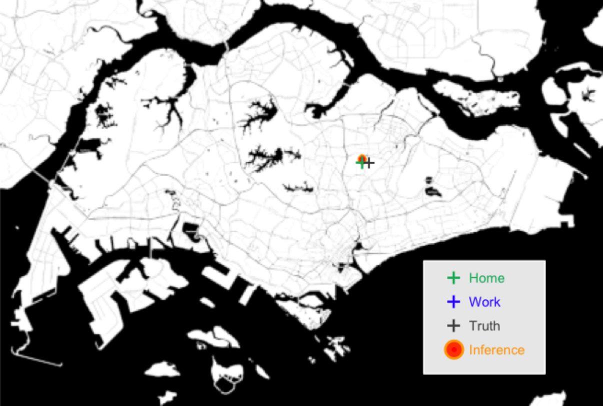
Home
Work
Truth
Inference

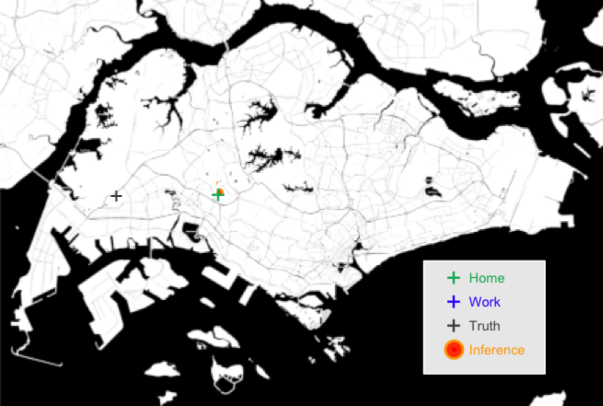
Home
Work
Truth
Inference

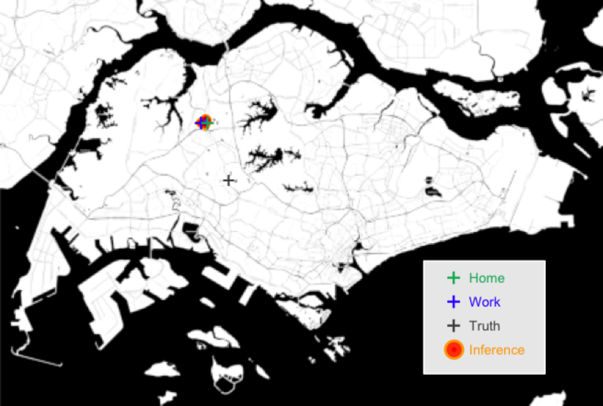
Home
Work
Truth
Inference

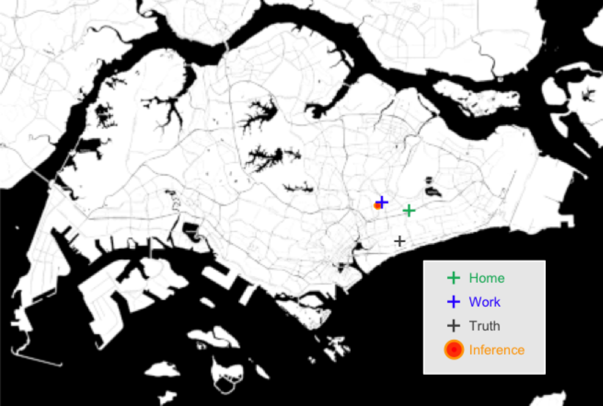

Home
Work
Truth
Inference

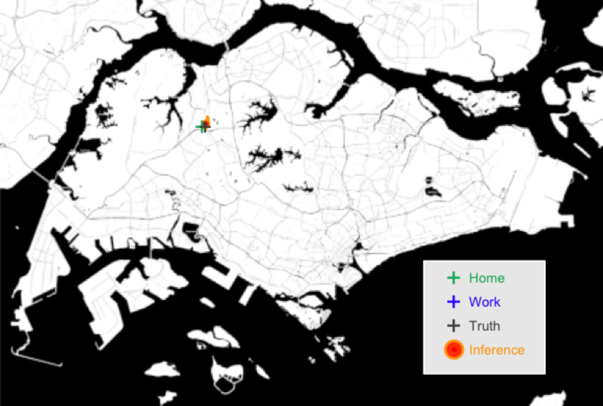

Home
Work
Truth
Inference

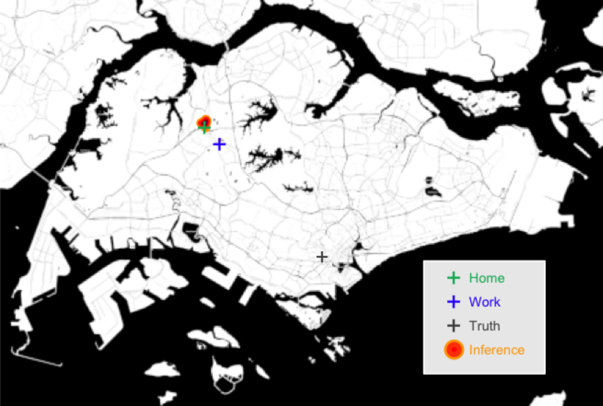

Home
Work
Truth
Inference

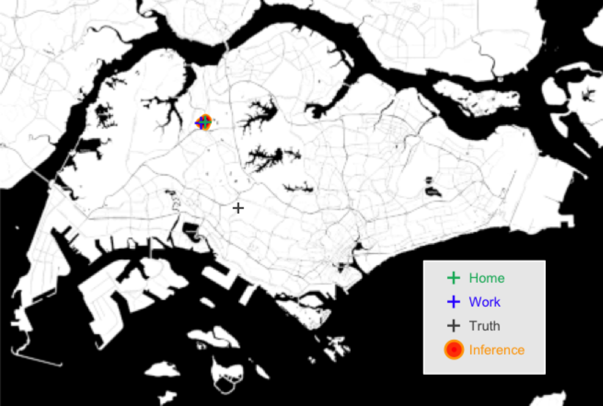
Home
Work
Truth
Inference

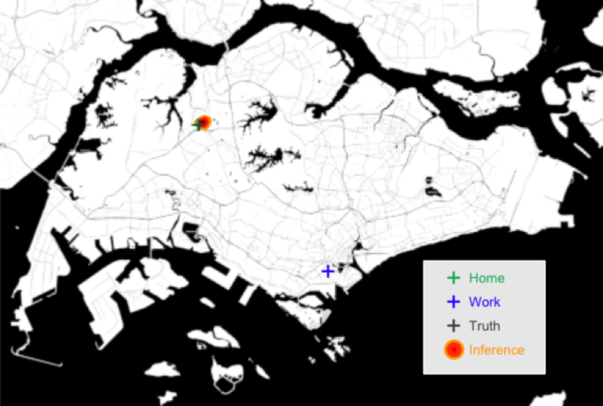
Home
Work
Truth
Inference

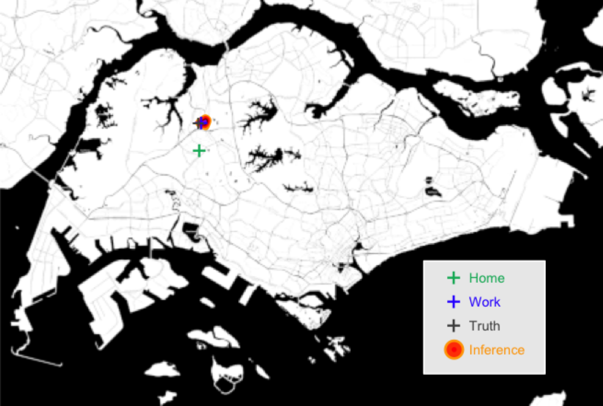
Home
Work
Truth
Inference

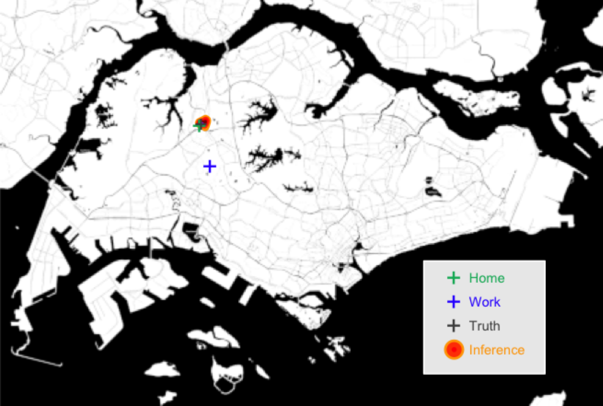
Home
Work
Truth
Inference

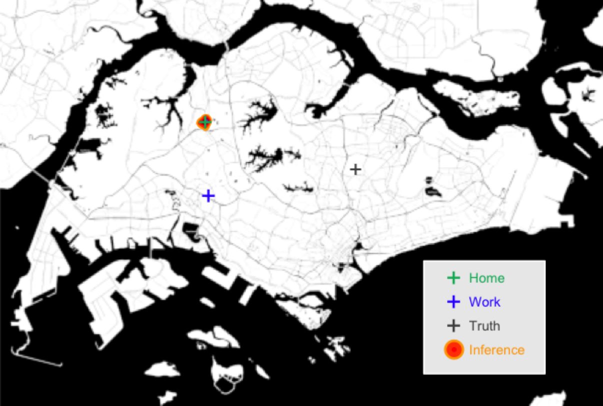

Home
Work
Truth
Inference

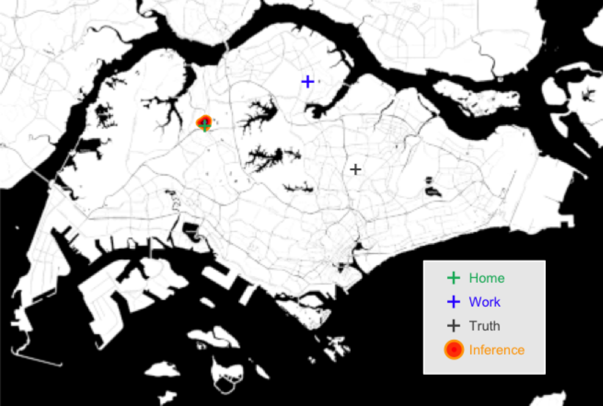
Home
Work
Truth
Inference

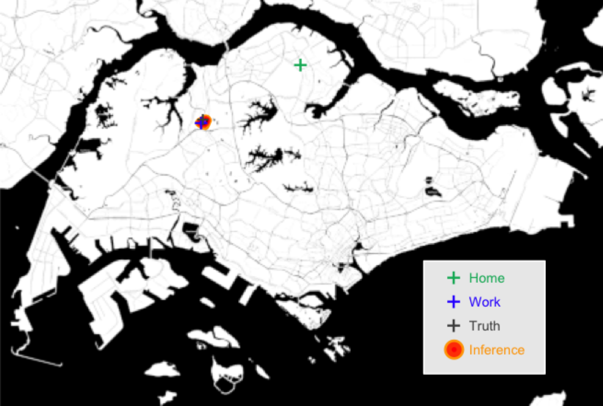
Home
Work
Truth
Inference

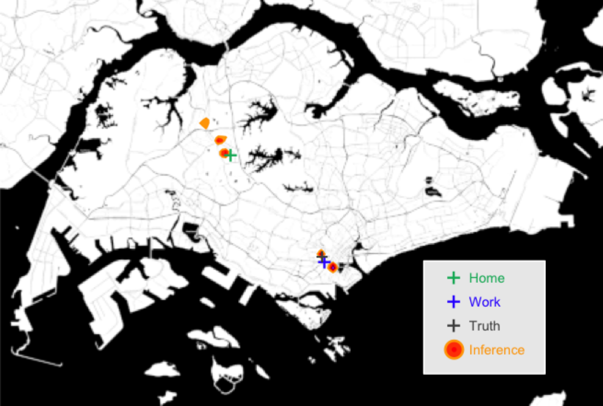
Home
Work
Truth
Inference

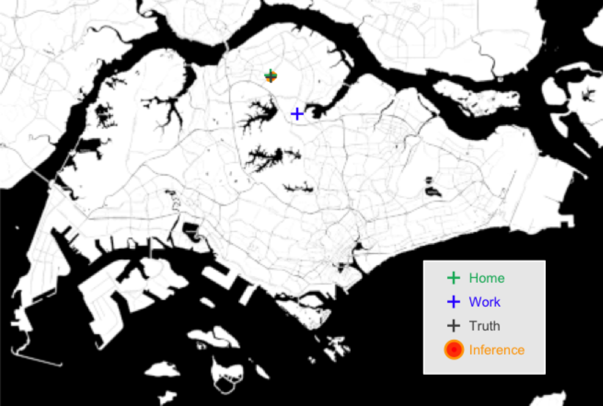
Home
Work
Truth
Inference

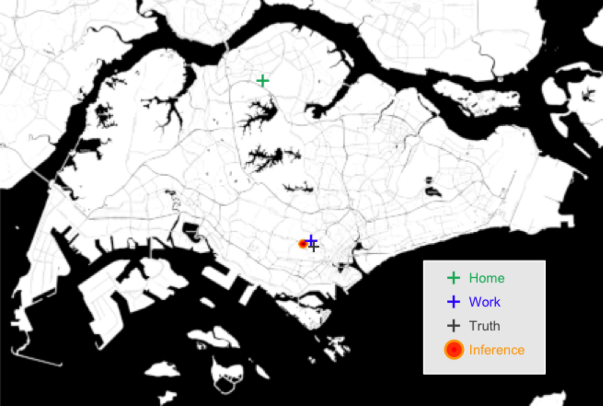
Home
Work
Truth
Inference

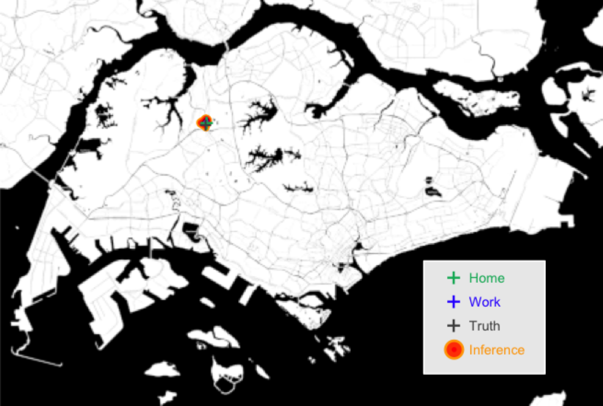
Home
Work
Truth
Inference

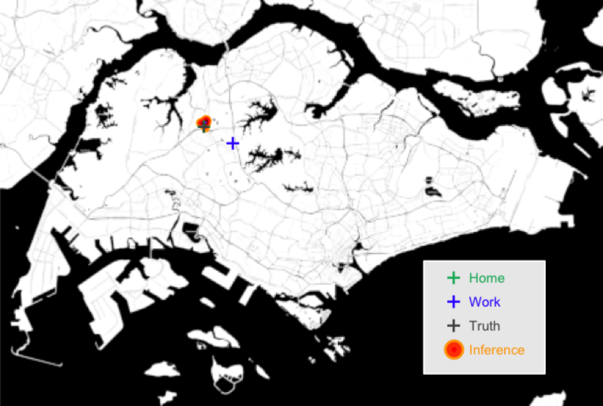
Home
Work
Truth
Inference

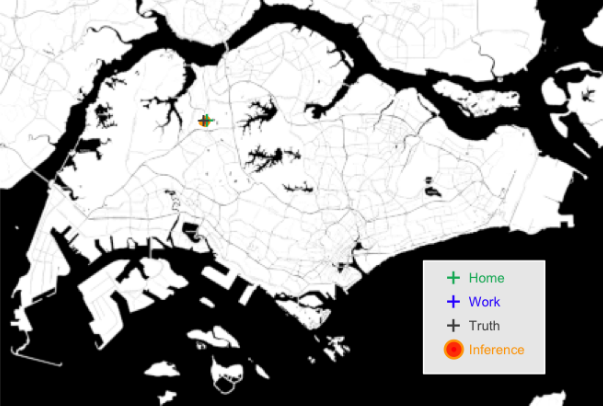

Home
Work
Truth
Inference

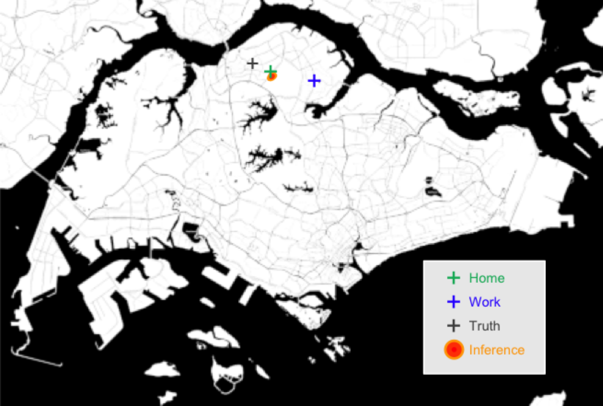
Home
Work
Truth
Inference

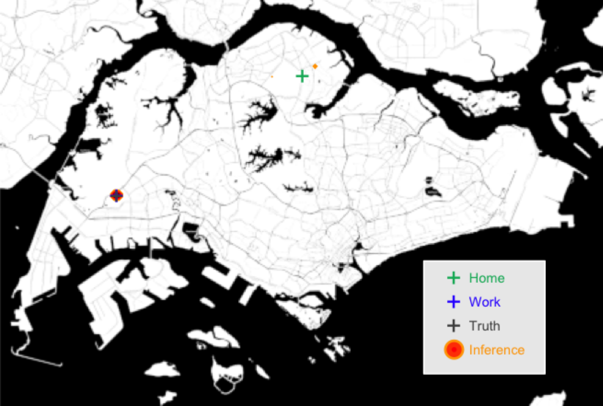
Home
Work
Truth
Inference

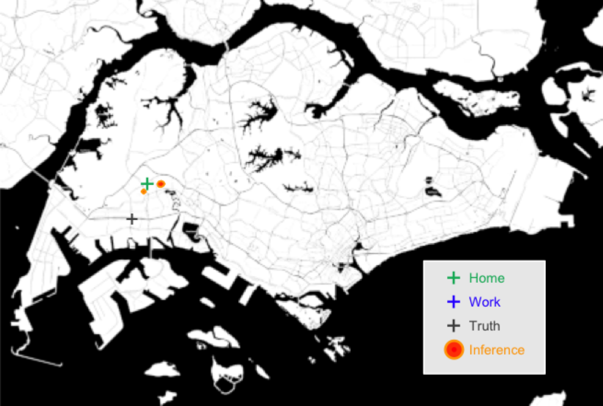
Home
Work
Truth
Inference

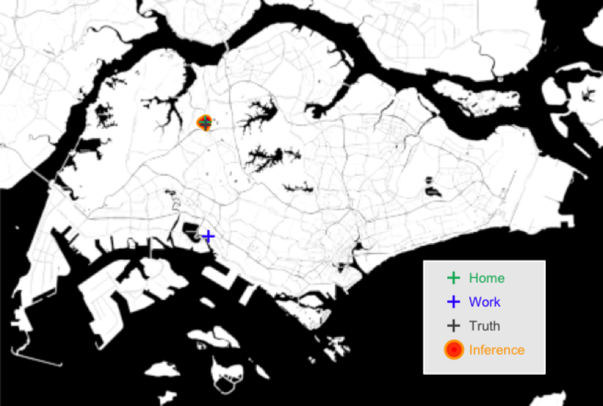

Home
Work
Truth
Inference

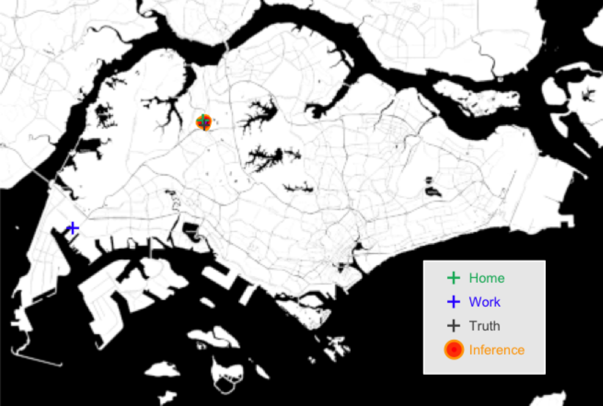
Home
Work
Truth
Inference

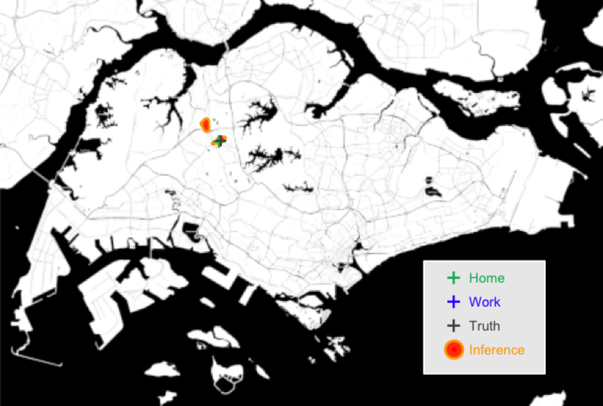
Home
Work
Truth
Inference

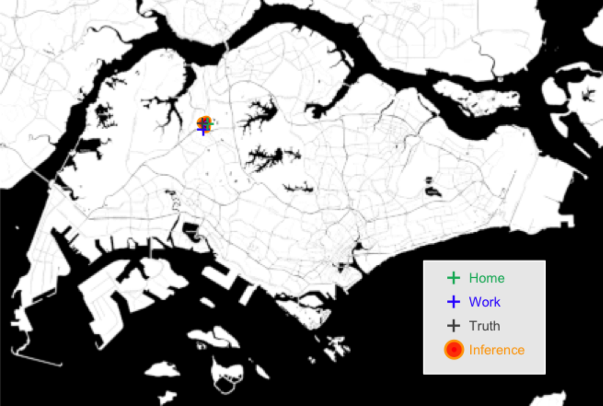
Home
Work
Truth
Inference

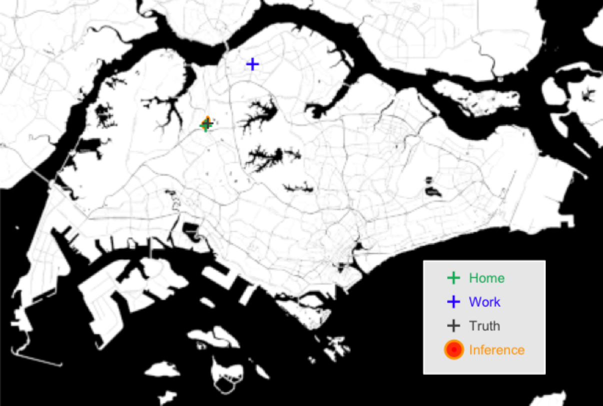
Home
Work
Truth
Inference

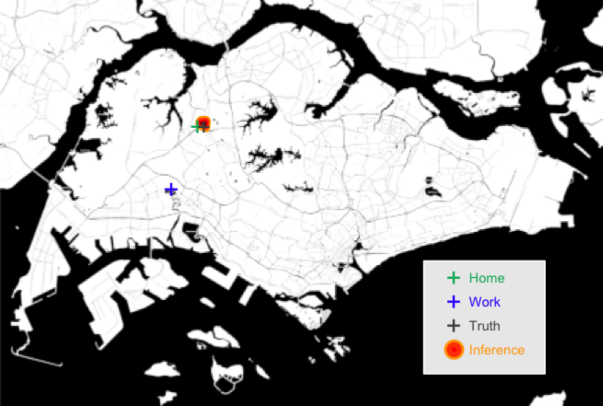
Home
Work
Truth
Inference

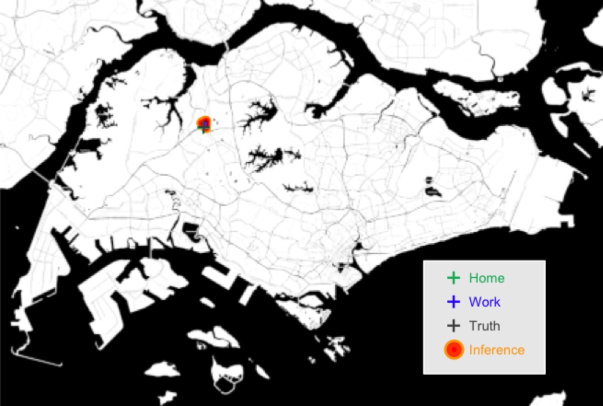
Home
Work
Truth
Inference

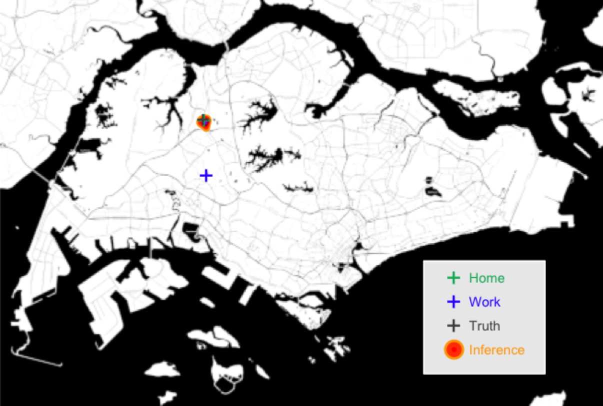
Home
Work
Truth
Inference

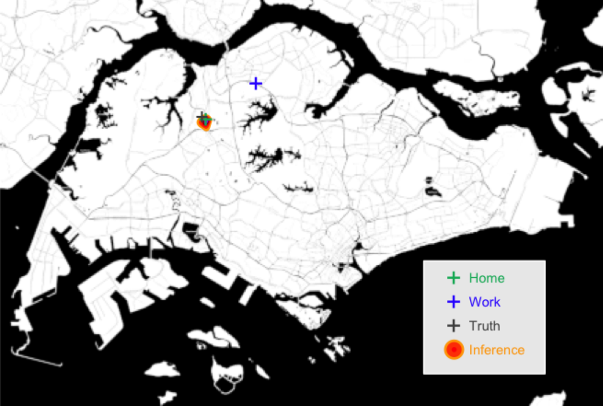
Home
Work
Truth
Inference

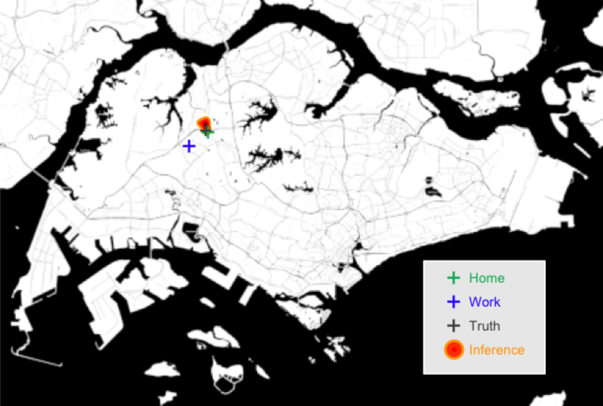
Home
Work
Truth
Inference

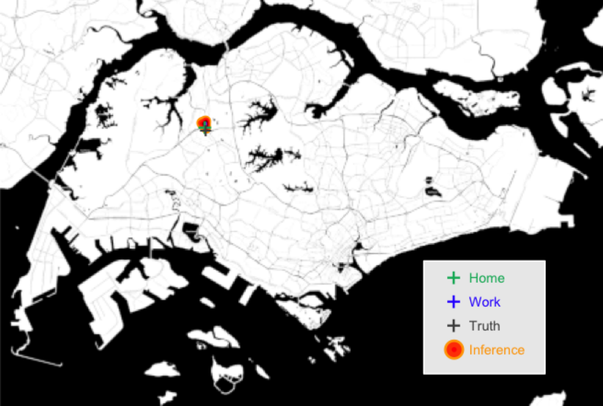
Home
Work
Truth
Inference

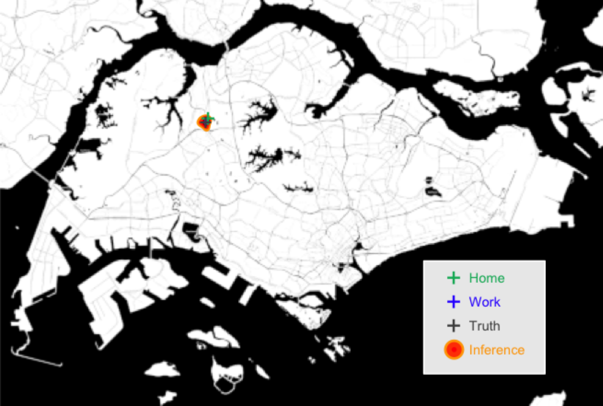
Home
Work
Truth
Inference

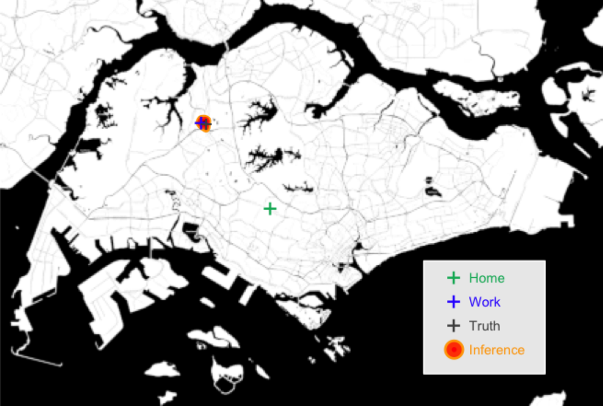
Home
Work
Truth
Inference

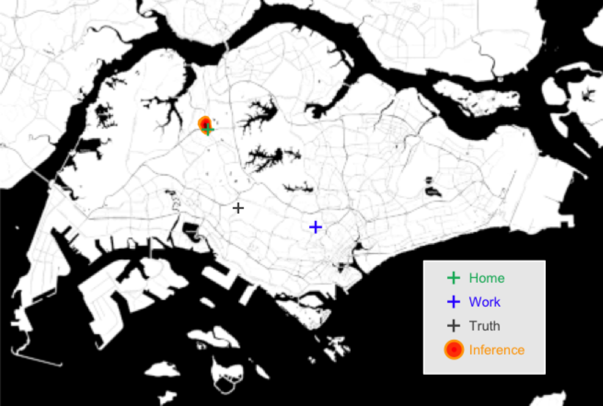
Home
Work
Truth
Inference

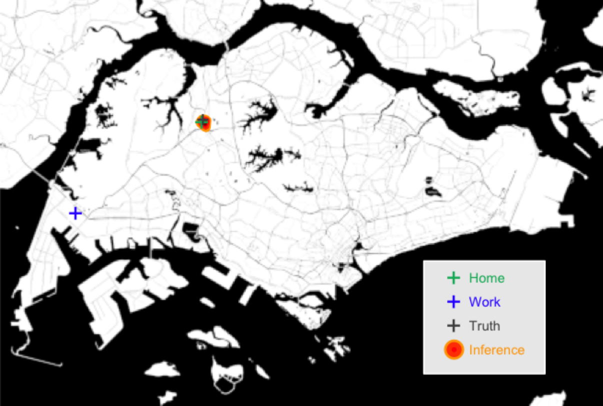
Home
Work
Truth
Inference

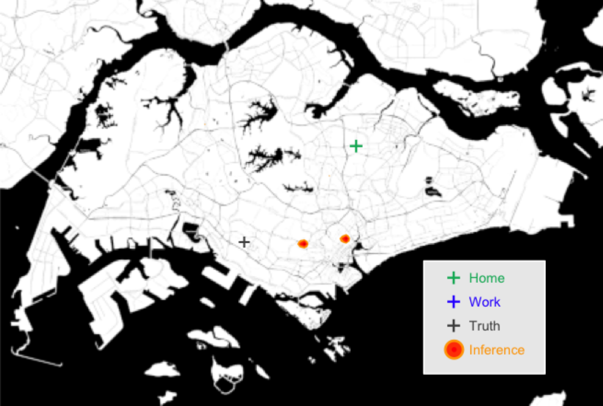
Home
Work
Truth
Inference

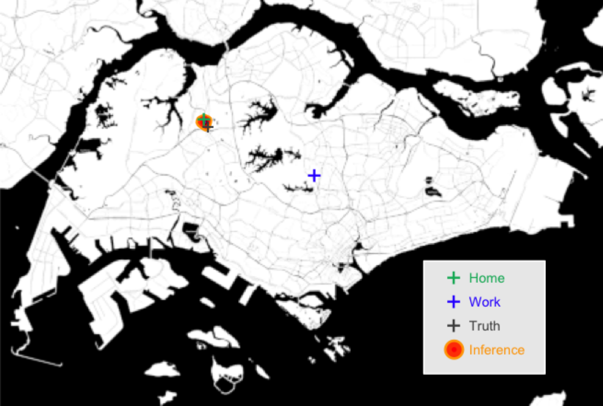
Home
Work
Truth
Inference

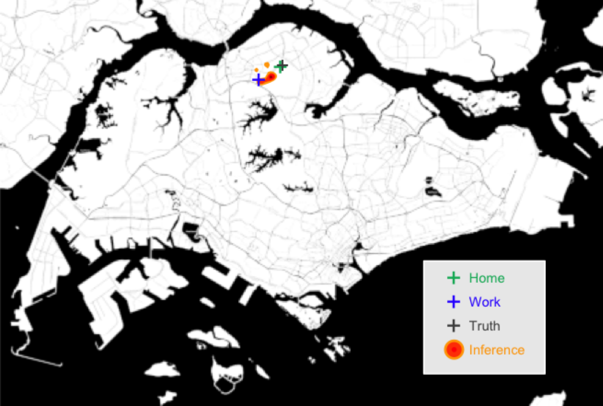
Home
Work
Truth
Inference

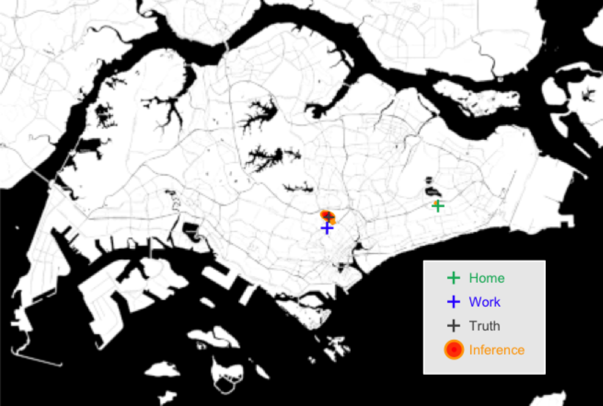

Home
Work
Truth
Inference

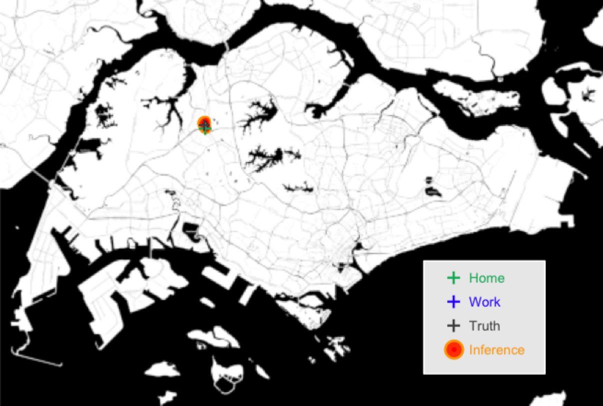

Home
Work
Truth
Inference

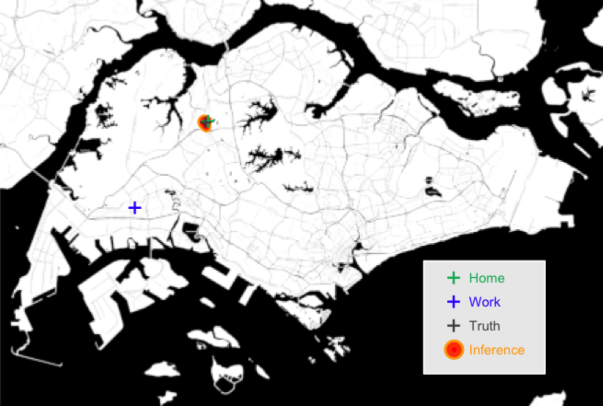

Home
Work
Truth
Inference

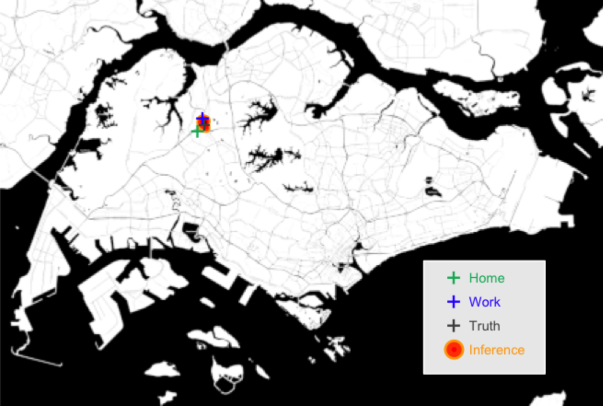
Home
Work
Truth
Inference

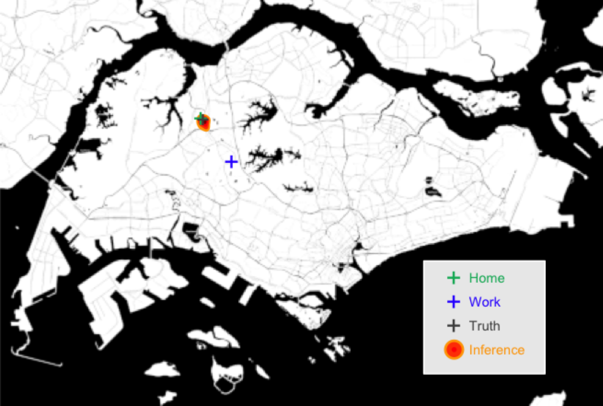

Home
Work
Truth
Inference

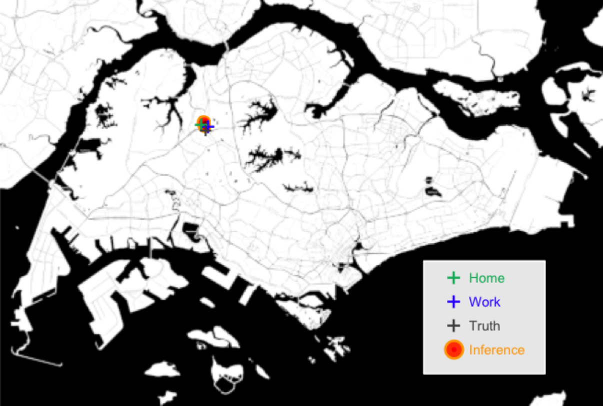
Home
Work
Truth
Inference

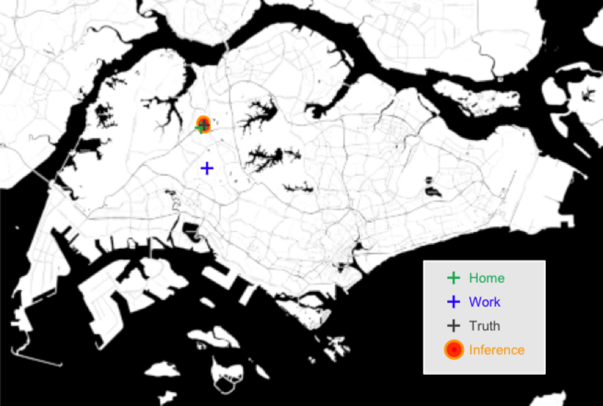

Home
Work
Truth
Inference

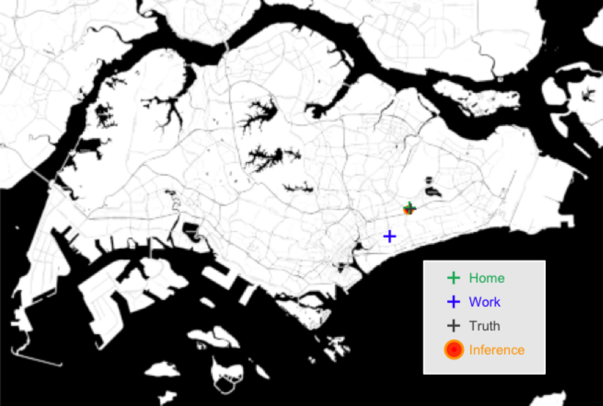

Home
Work
Truth
Inference

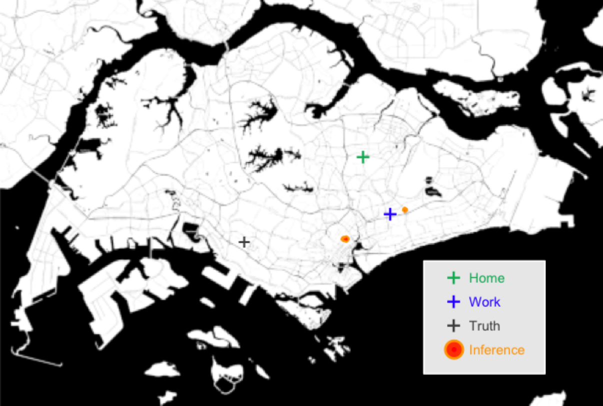
Home
Work
Truth
Inference

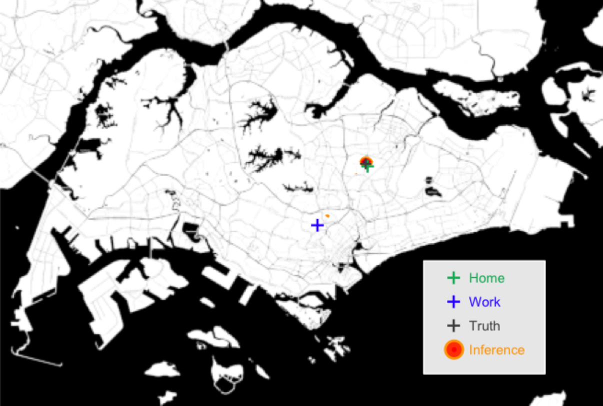
Home
Work
Truth
Inference

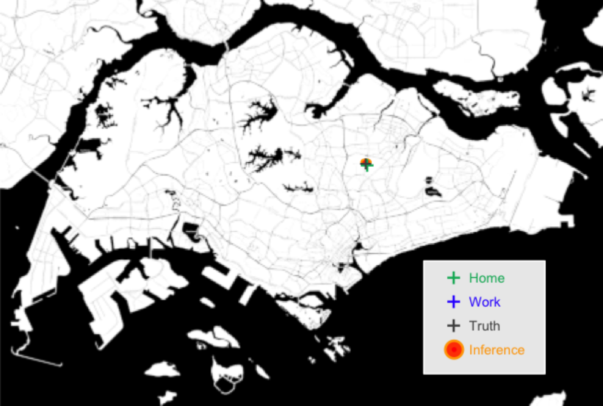
Home
Work
Truth
Inference

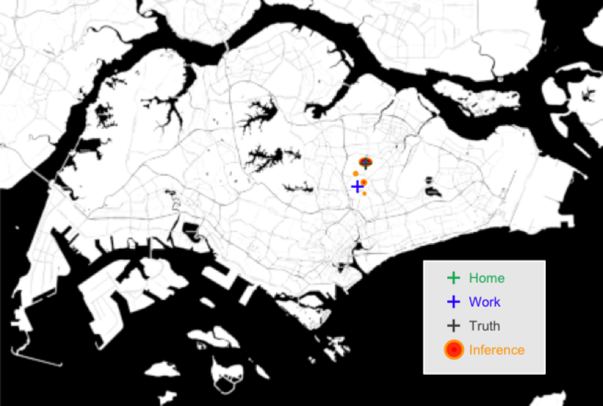
Home
Work
Truth
Inference

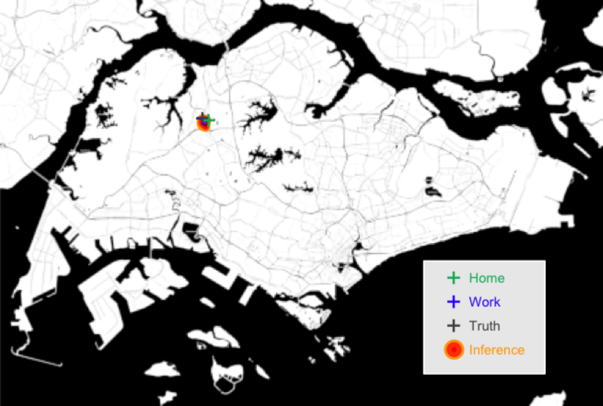

Home
Work
Truth
Inference

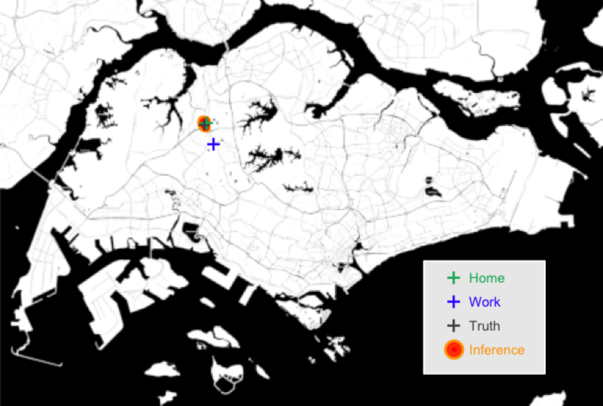
Home
Work
Truth
Inference

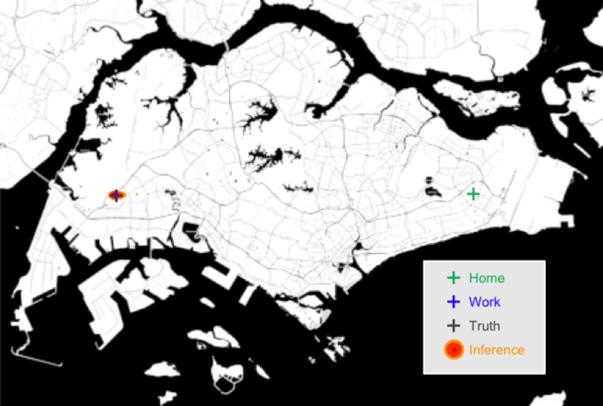
Home
Work
Truth
Inference

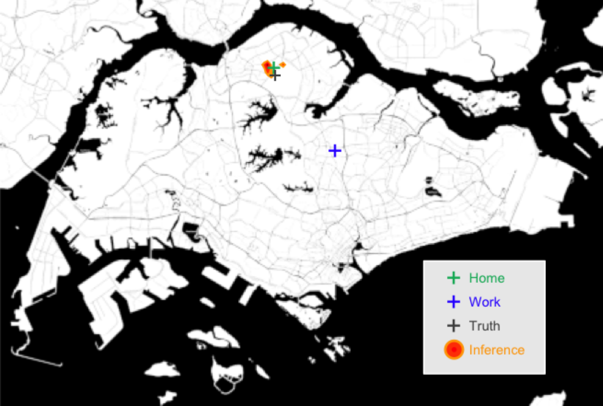

Home
Work
Truth
Inference

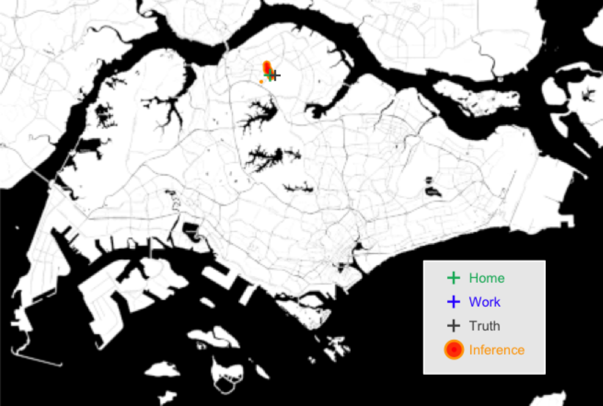
Home
Work
Truth
Inference

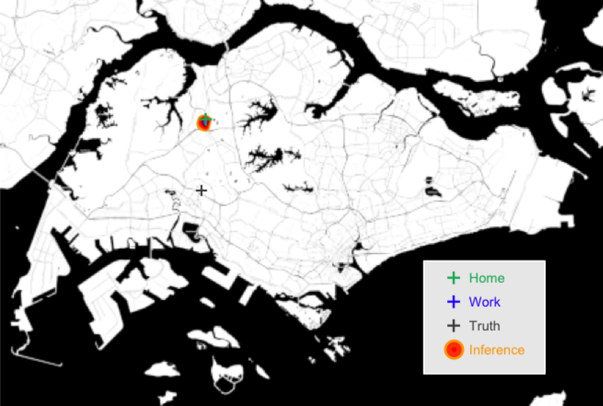
Home
Work
Truth
Inference

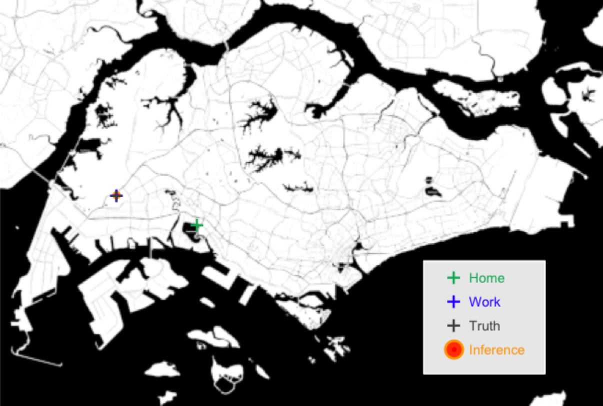
Home
Work
Truth
Inference

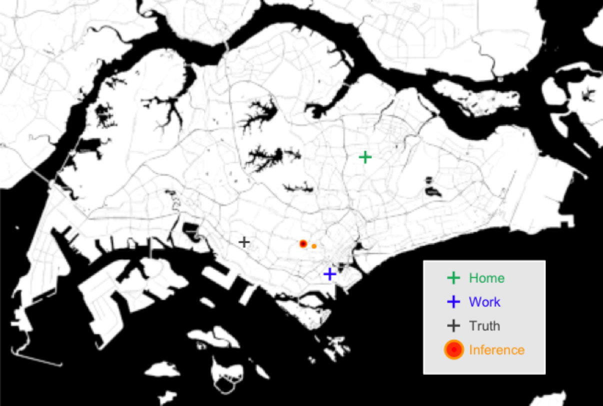
Home
Work
Truth
Inference

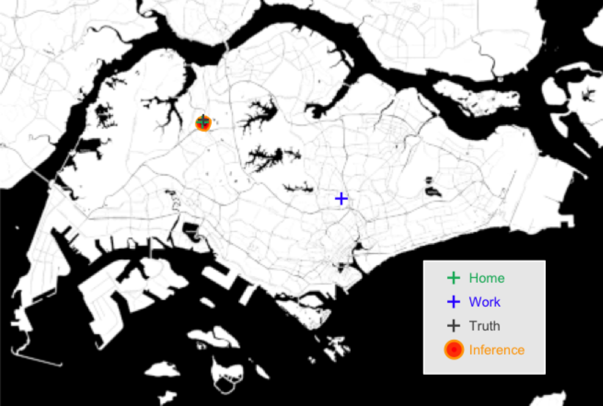
Home
Work
Truth
Inference

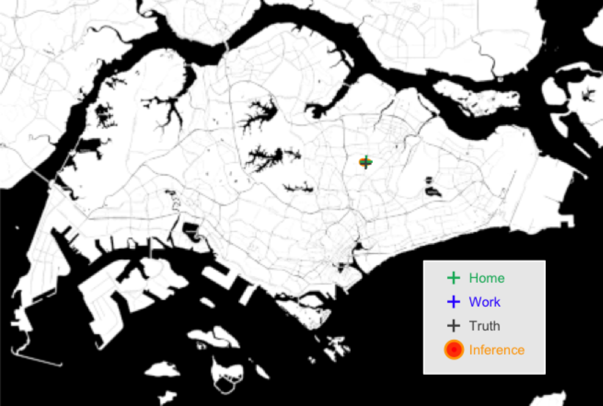
Home
Work
Truth
Inference

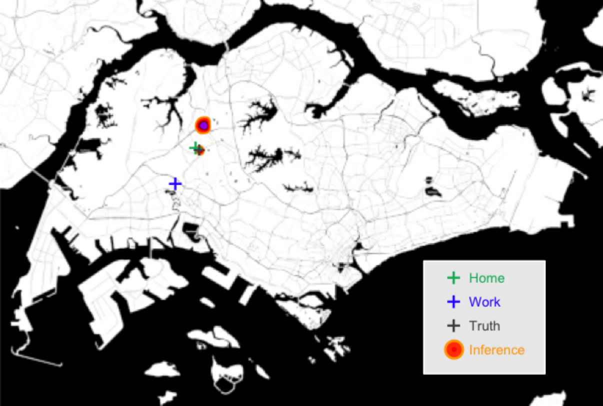
Home
Work
Truth
Inference

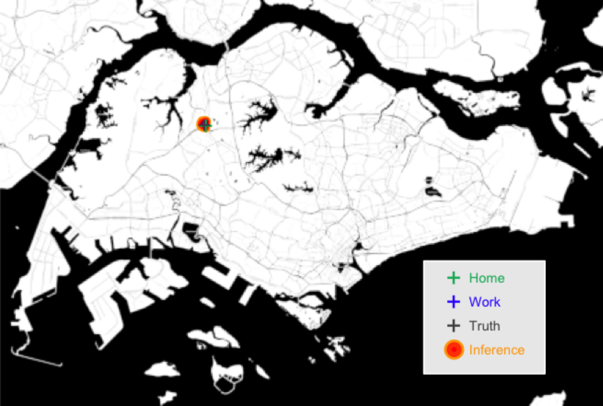
Home
Work
Truth
Inference

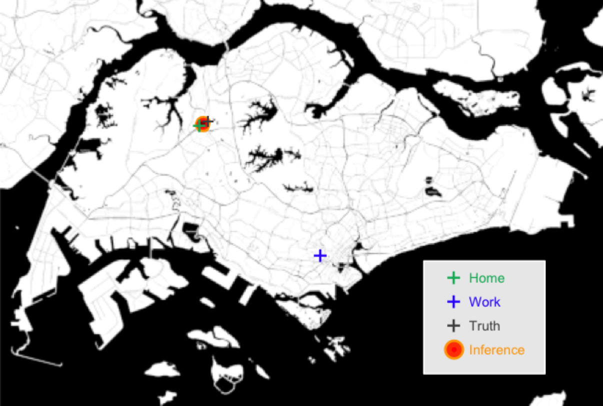
Home
Work
Truth
Inference

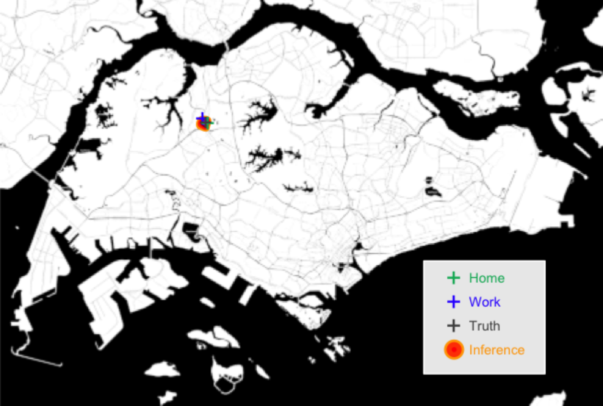
Home
Work
Truth
Inference

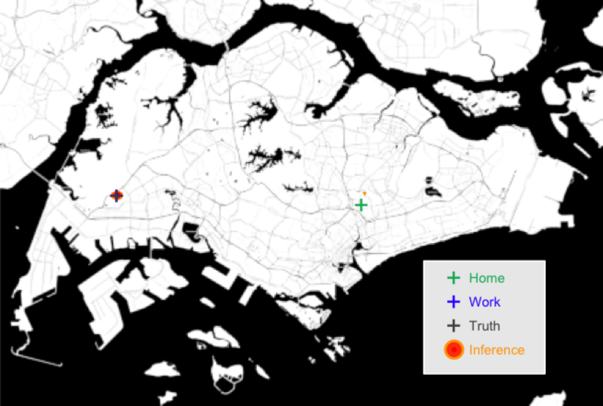
Home
Work
Truth
Inference

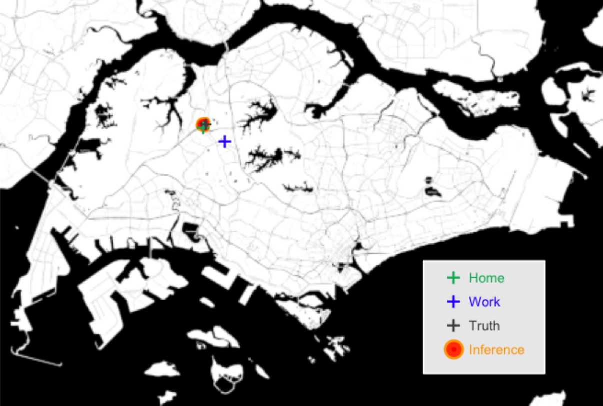
Home
Work
Truth
Inference

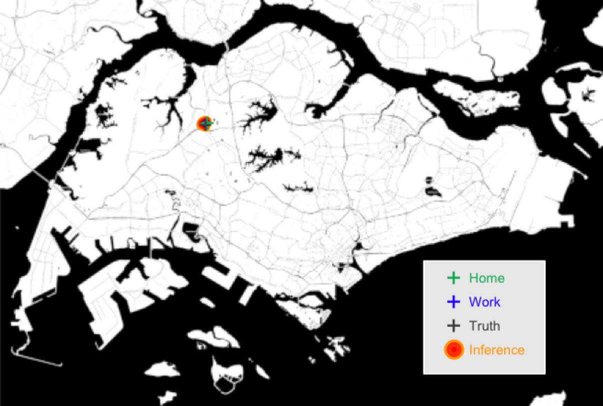
Home
Work
Truth
Inference

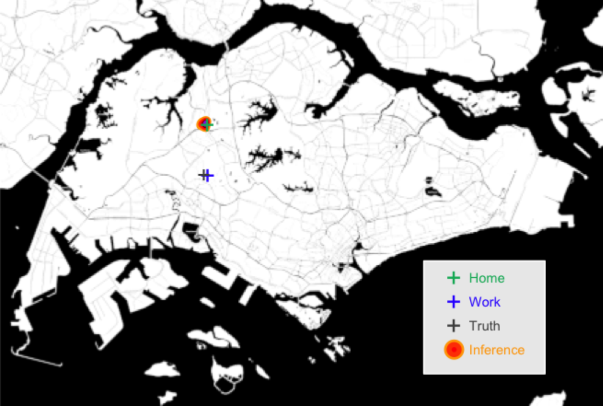
Home
Work
Truth
Inference

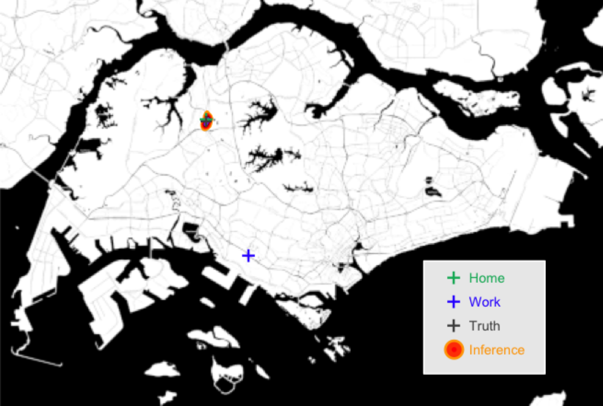

Home
Work
Truth
Inference

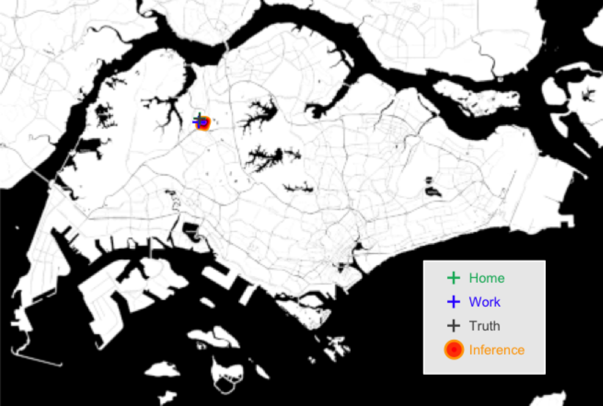
Home
Work
Truth
Inference

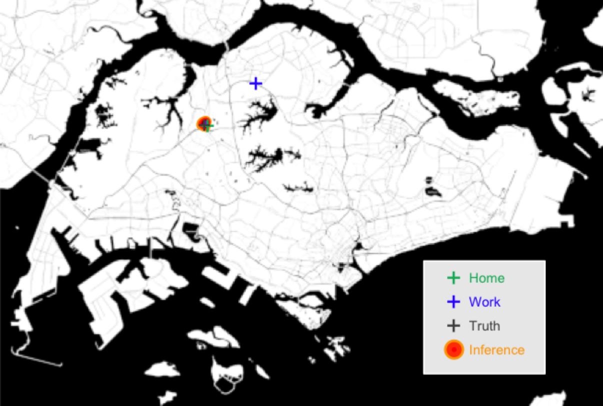
Home
Work
Truth
Inference

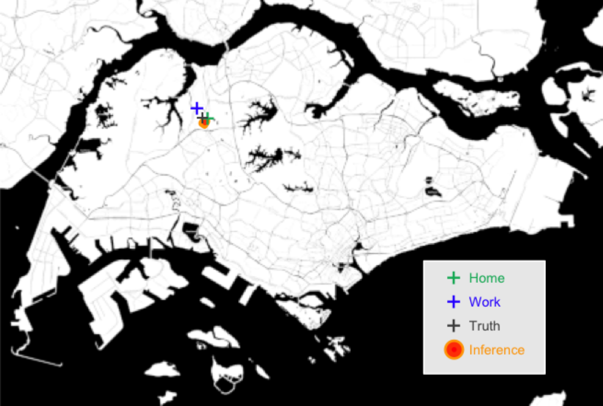
Home
Work
Truth
Inference

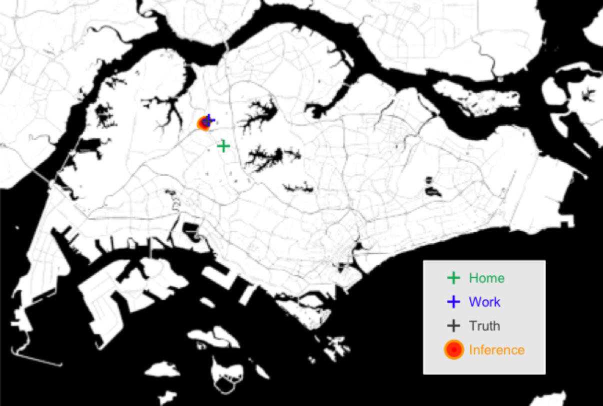
Home
Work
Truth
Inference

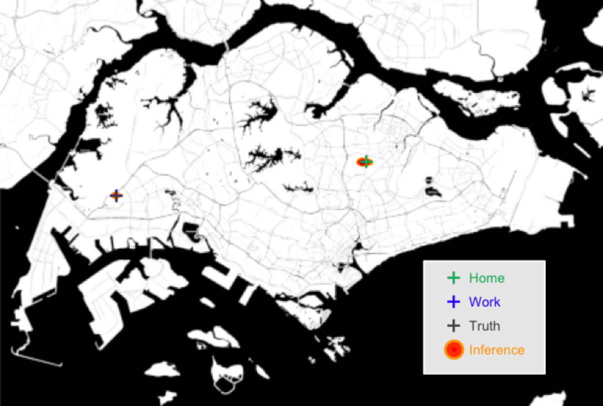
Home
Work
Truth
Inference

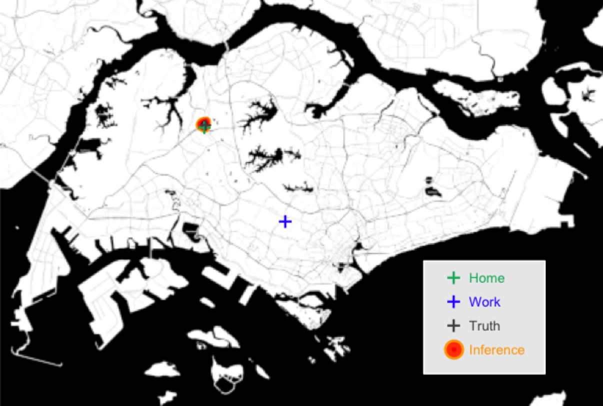
Home
Work
Truth
Inference

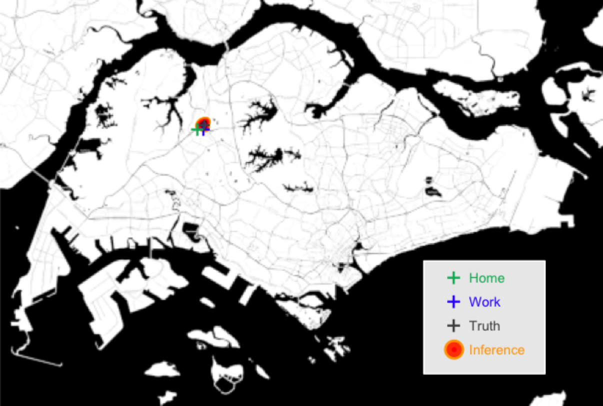
Home
Work
Truth
Inference

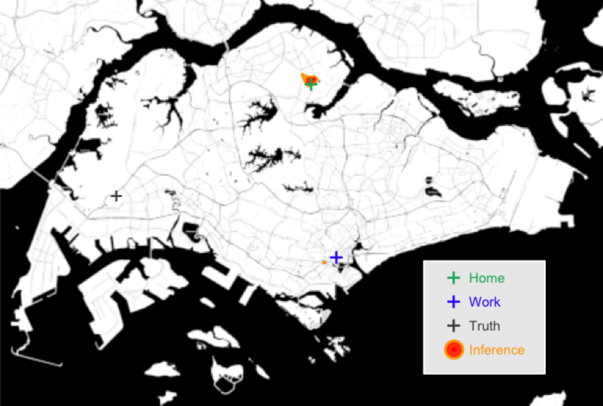

Home
Work
Truth
Inference

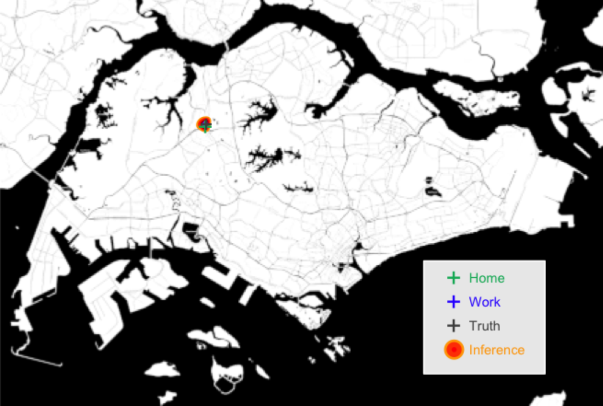
Home
Work
Truth
Inference

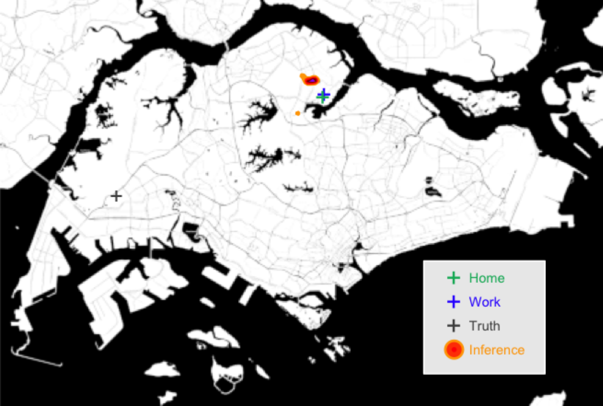
Home
Work
Truth
Inference

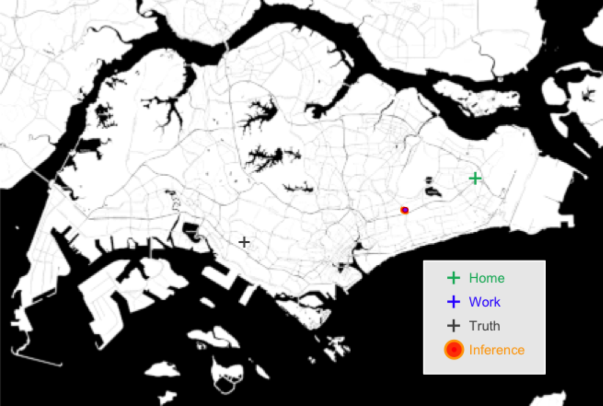

Home
Work
Truth
Inference

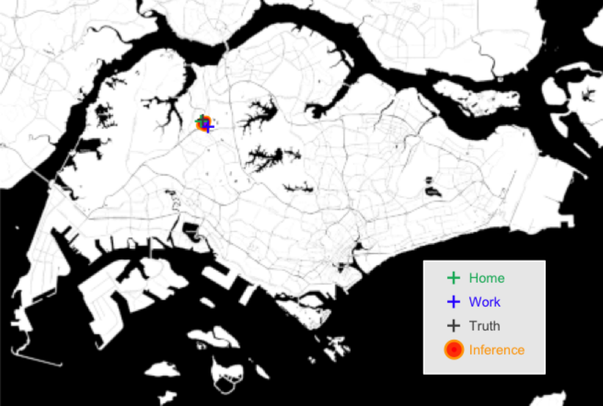
Home
Work
Truth
Inference

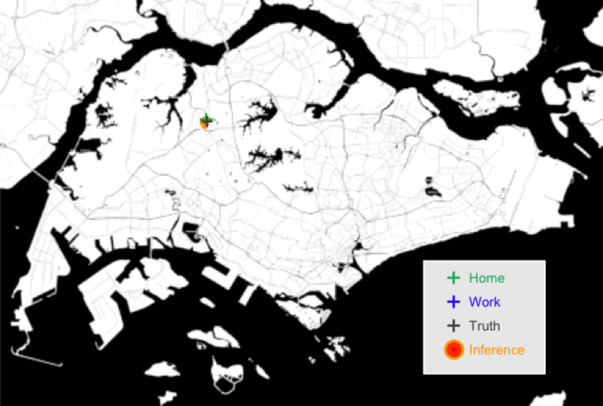

Home
Work
Truth
Inference

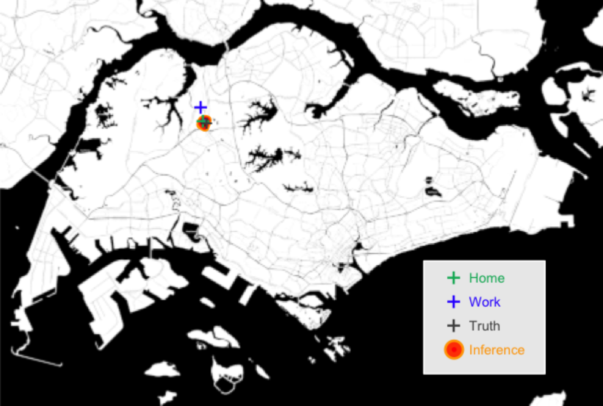
Home
Work
Truth
Inference

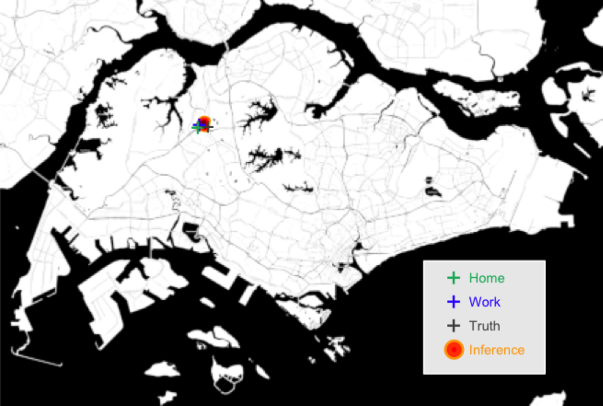
Home
Work
Truth
Inference

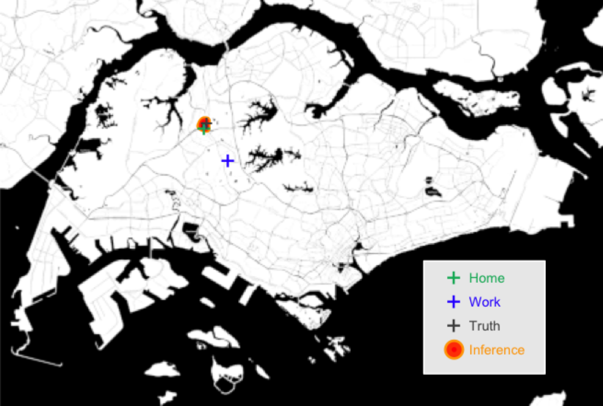

Home
Work
Truth
Inference

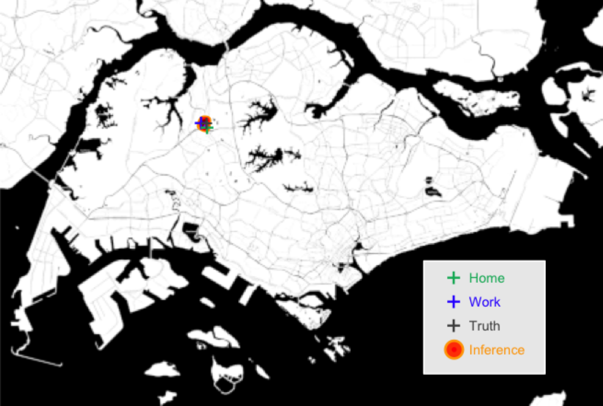
Home
Work
Truth
Inference

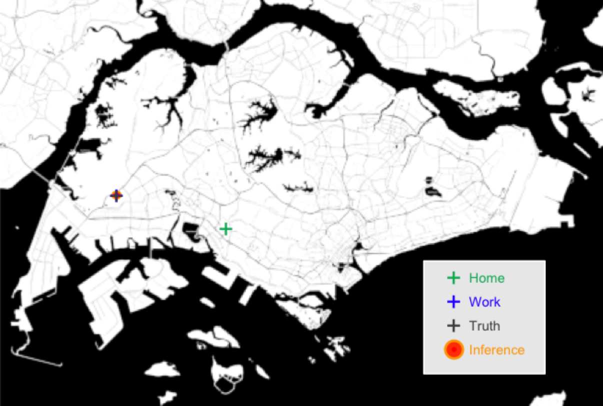

Home
Work
Truth
Inference

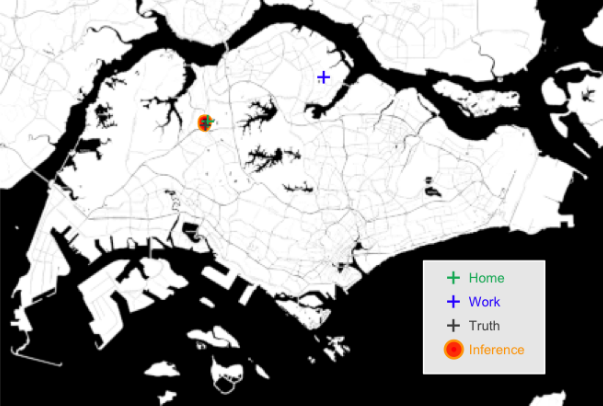
Home
Work
Truth
Inference

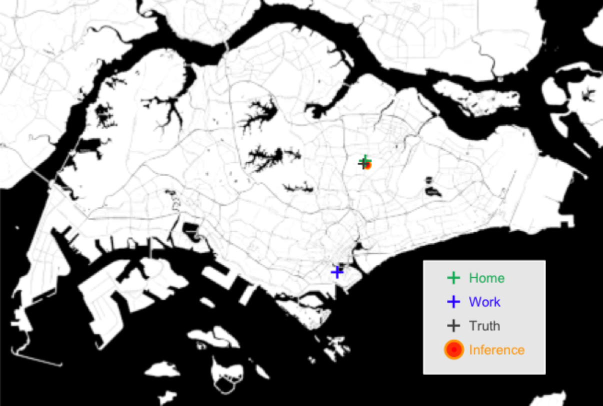
Home
Work
Truth
Inference

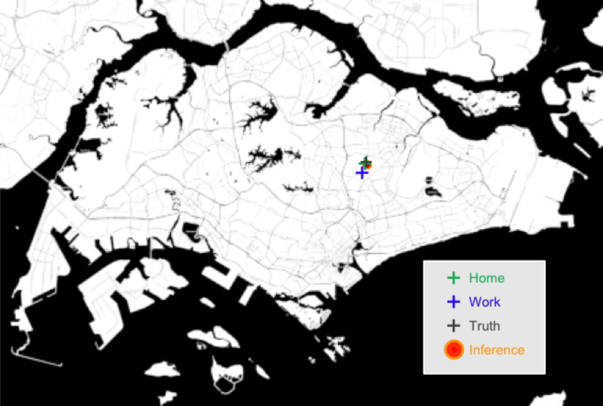
Home
Work
Truth
Inference

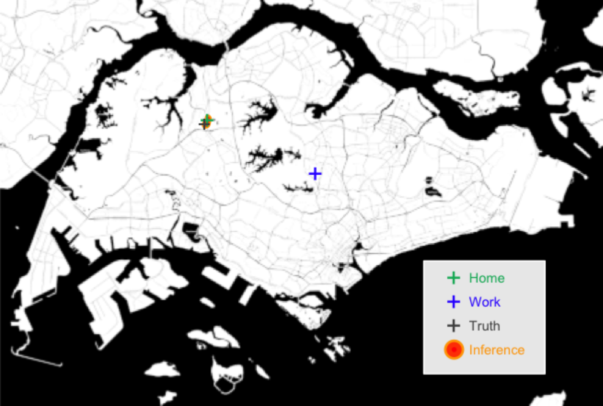

Home
Work
Truth
Inference

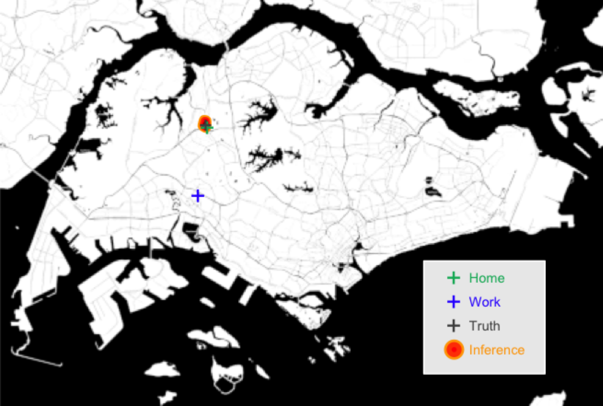
Home
Work
Truth
Inference

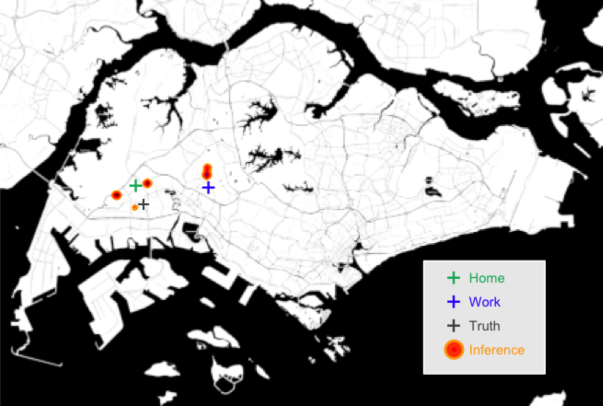
Home
Work
Truth
Inference

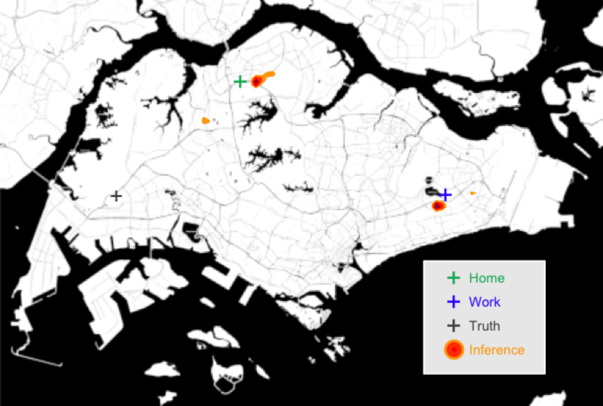
Home
Work
Truth
Inference

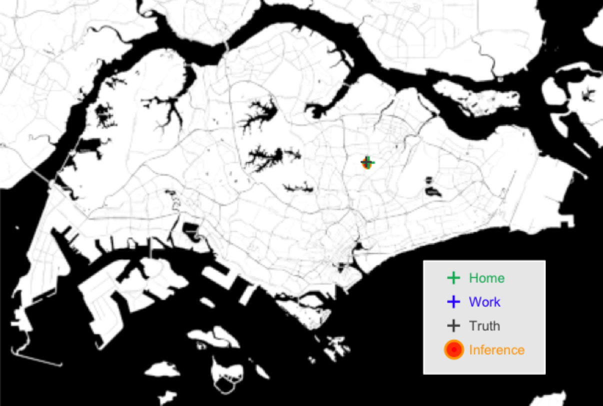

Home
Work
Truth
Inference

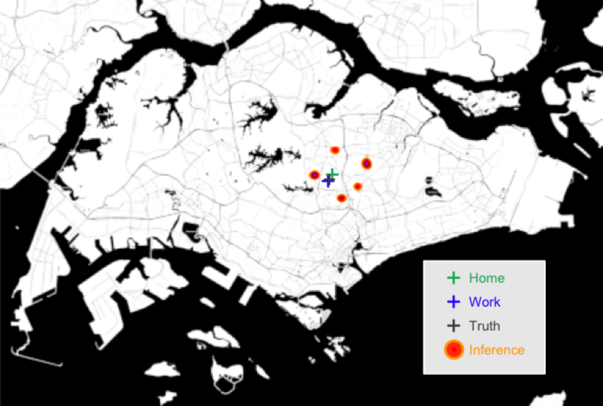
Home
Work
Truth
Inference

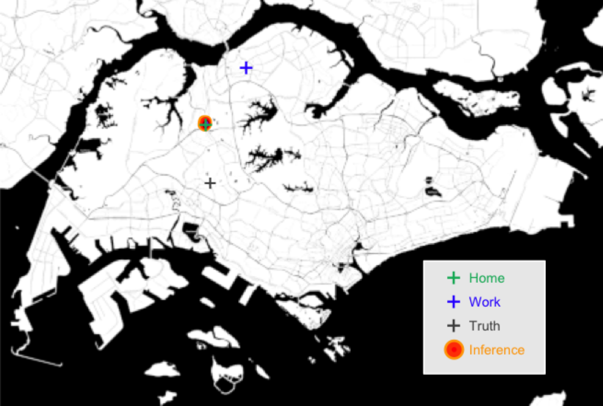
Home
Work
Truth
Inference

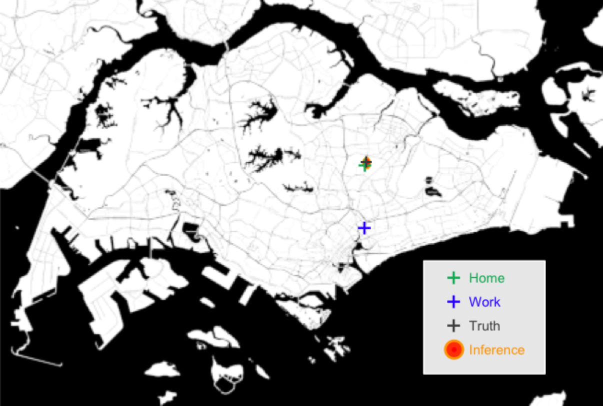
Home
Work
Truth
Inference

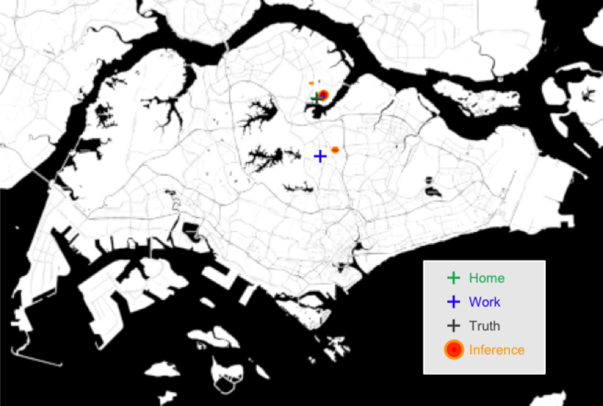
Home
Work
Truth
Inference

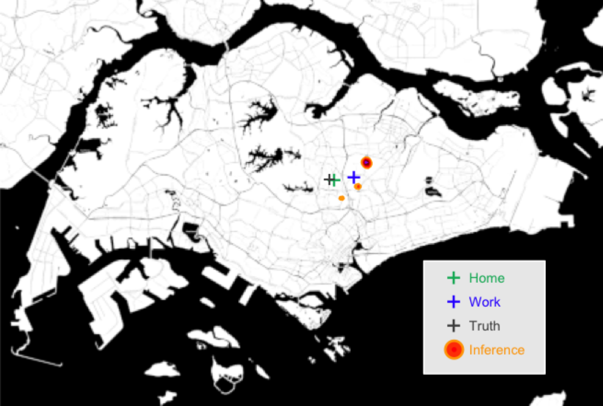
Home
Work
Truth
Inference

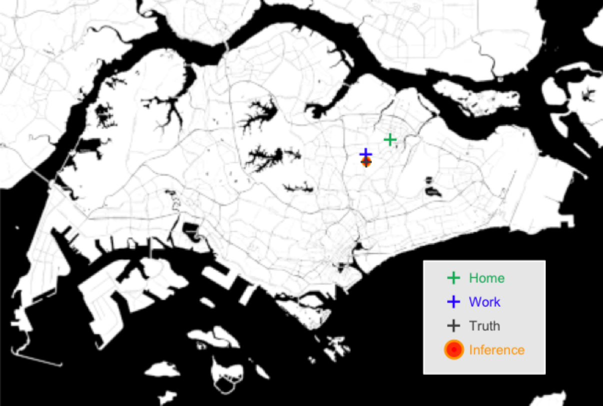
Home
Work
Truth
Inference

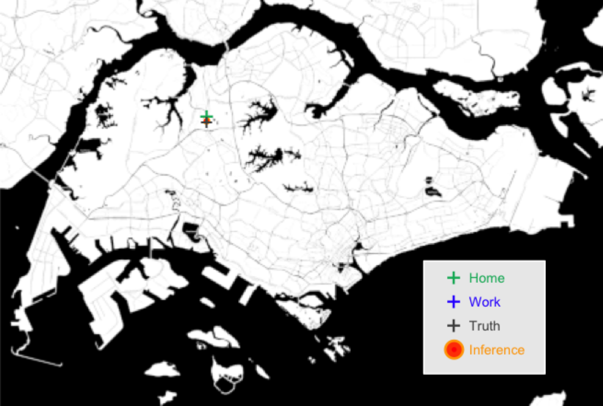

Home
Work
Truth
Inference

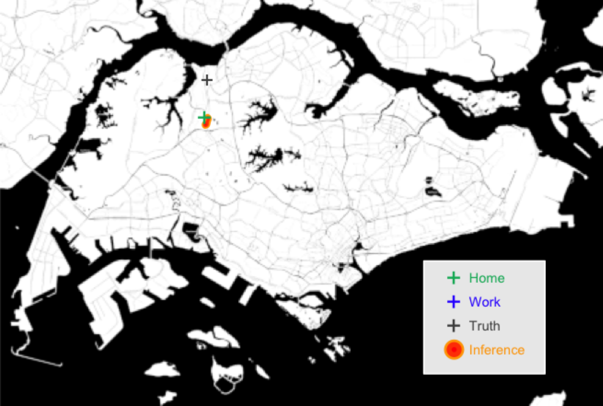

Home
Work
Truth
Inference

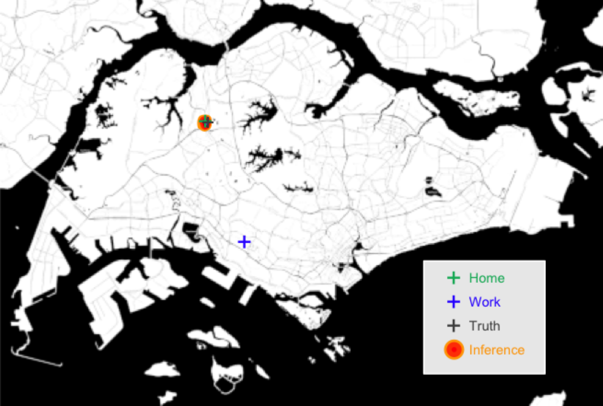
Home
Work
Truth
Inference

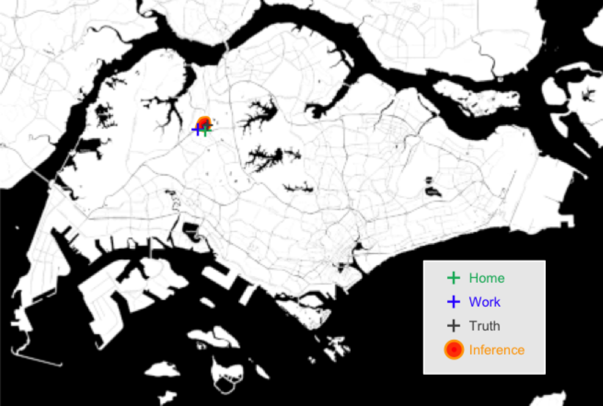
Home
Work
Truth
Inference

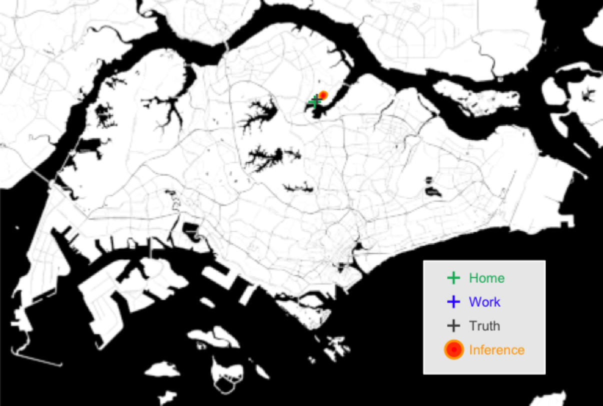
Home
Work
Truth
Inference

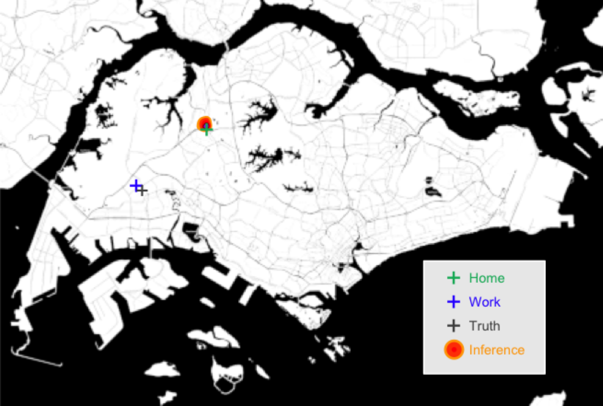

Home
Work
Truth
Inference

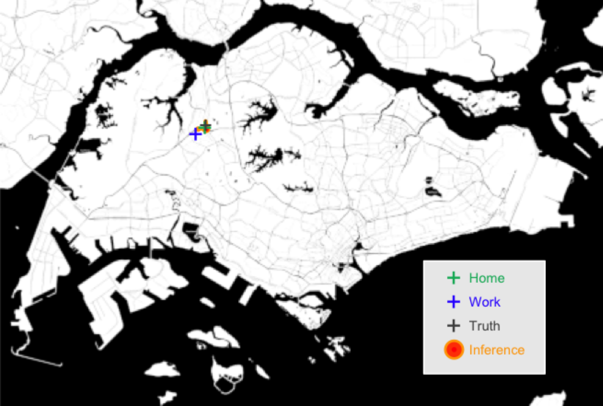

Home
Work
Truth
Inference

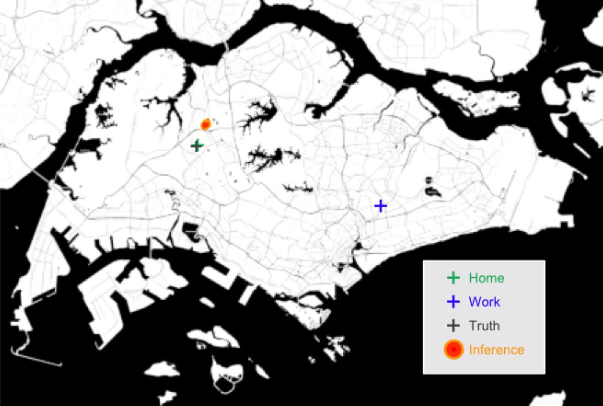
Home
Work
Truth
Inference

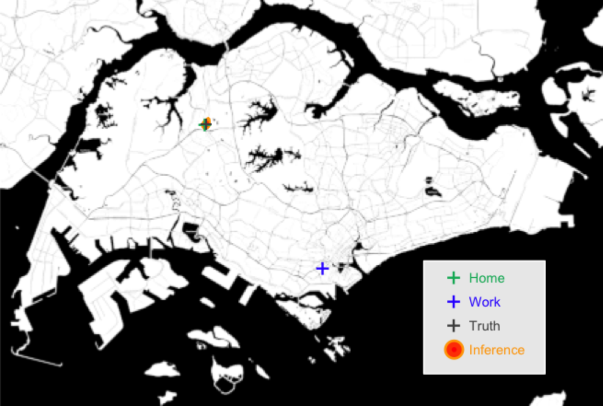
Home
Work
Truth
Inference

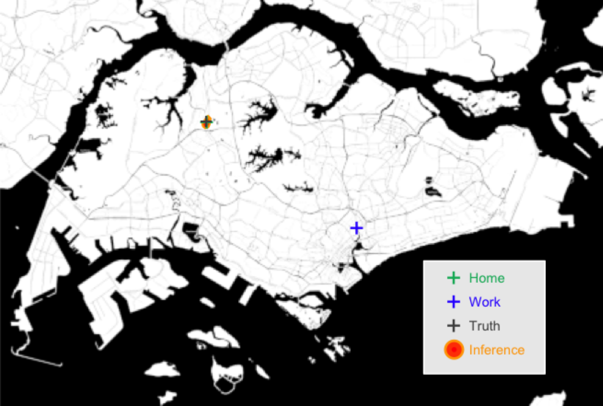
Home
Work
Truth
Inference

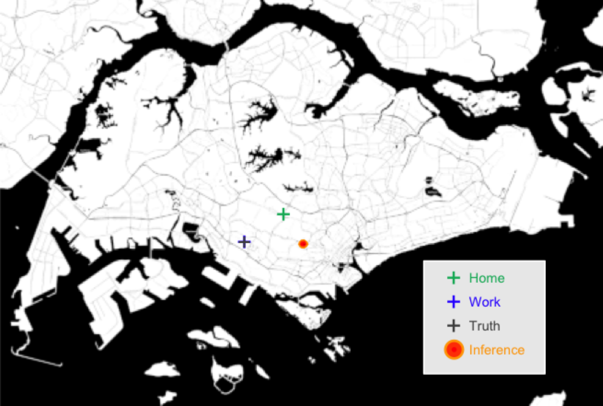
Home
Work
Truth
Inference

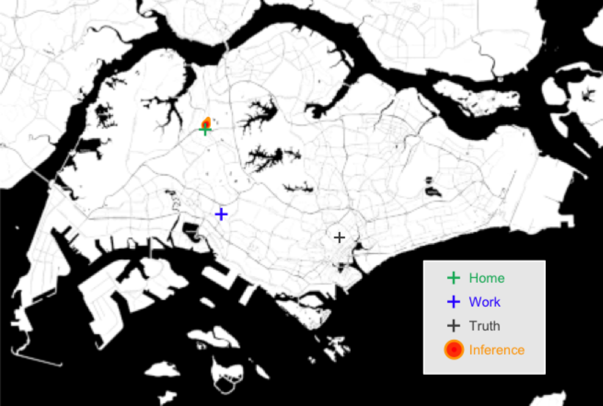
Home
Work
Truth
Inference

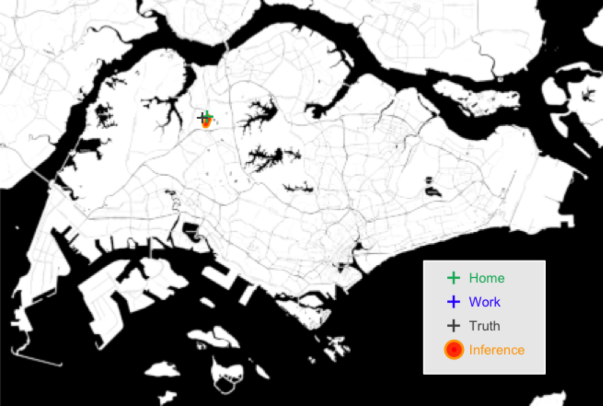

Home
Work
Truth
Inference

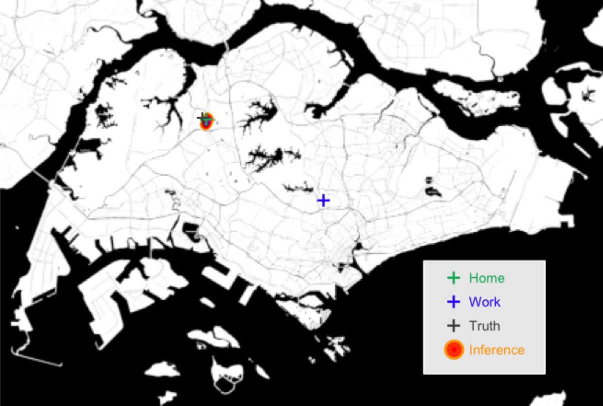
Home
Work
Truth
Inference

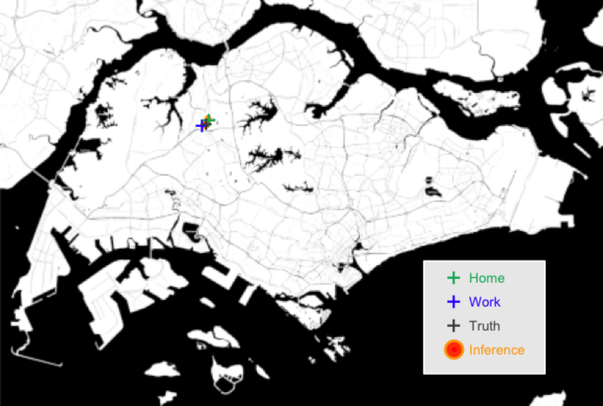

Home
Work
Truth
Inference

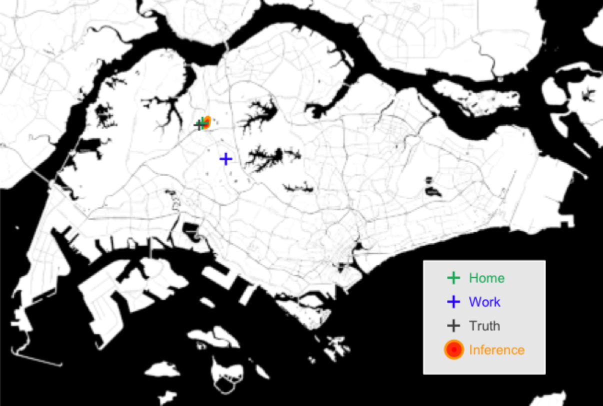
Home
Work
Truth
Inference

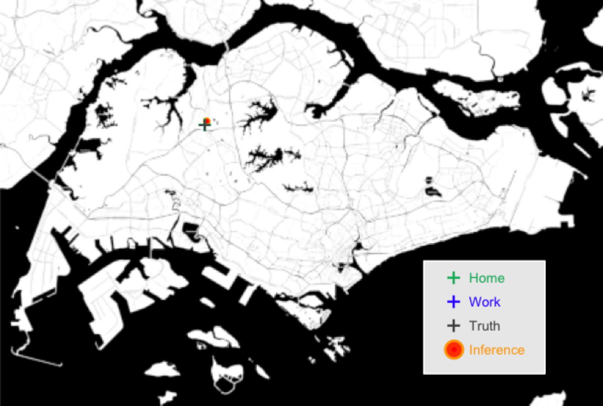
Home
Work
Truth
Inference

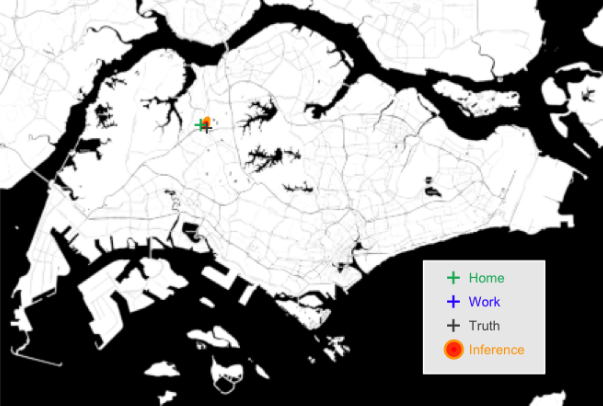
Home
Work
Truth
Inference

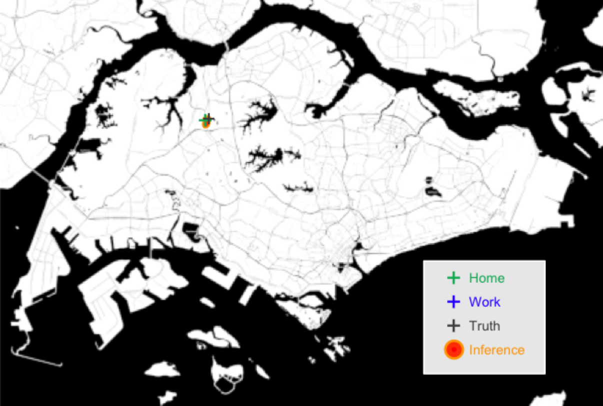
Home
Work
Truth
Inference

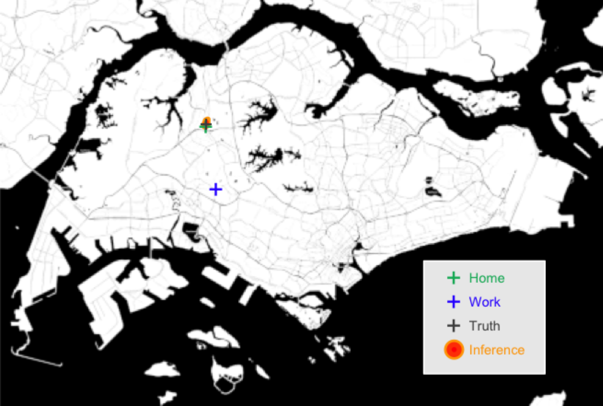
Home
Work
Truth
Inference

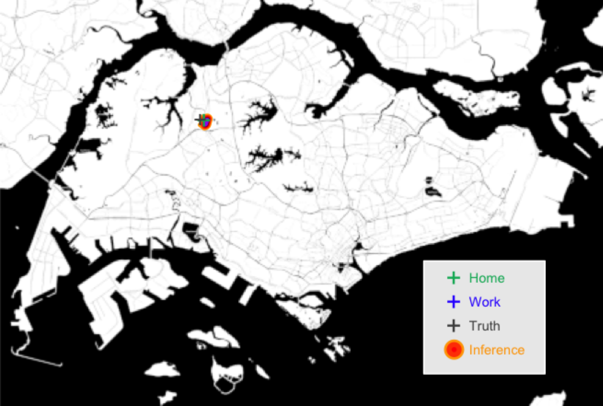
Home
Work
Truth
Inference

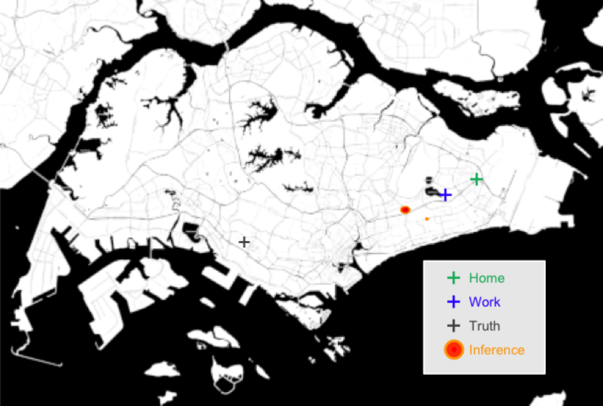
Home
Work
Truth
Inference

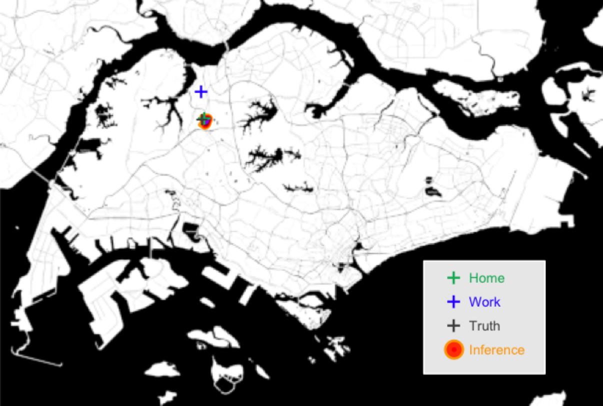
Home
Work
Truth
Inference

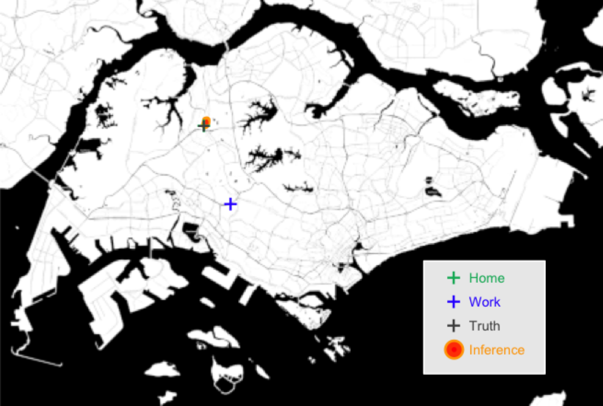
Home
Work
Truth
Inference

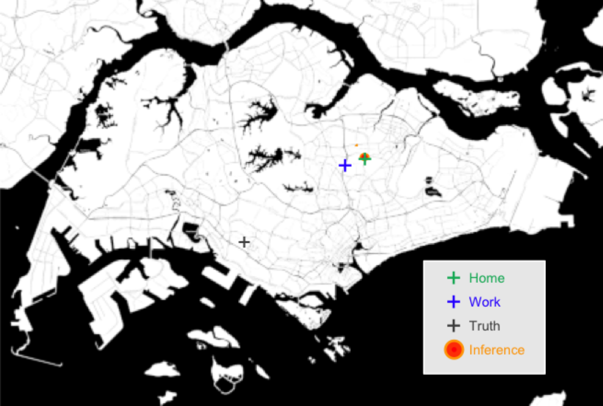

Home
Work
Truth
Inference

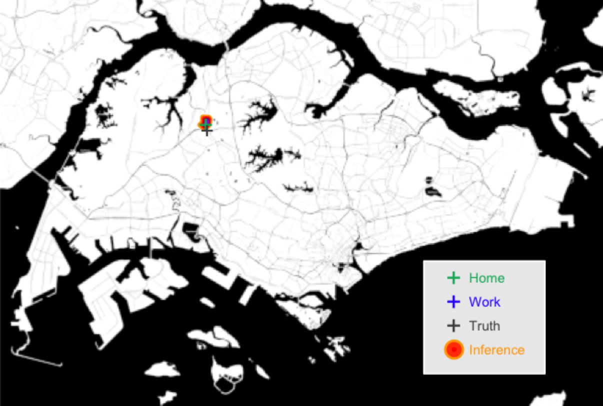
Home
Work
Truth
Inference

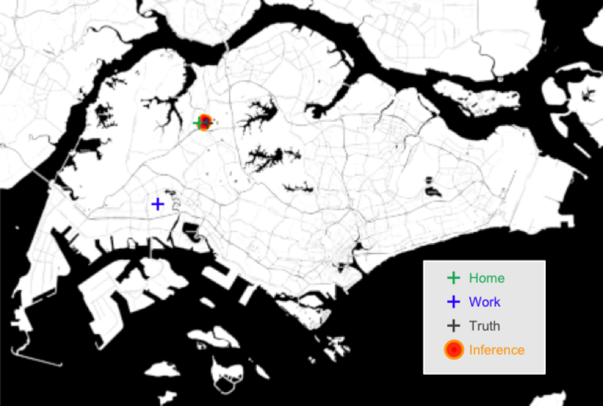

Home
Work
Truth
Inference

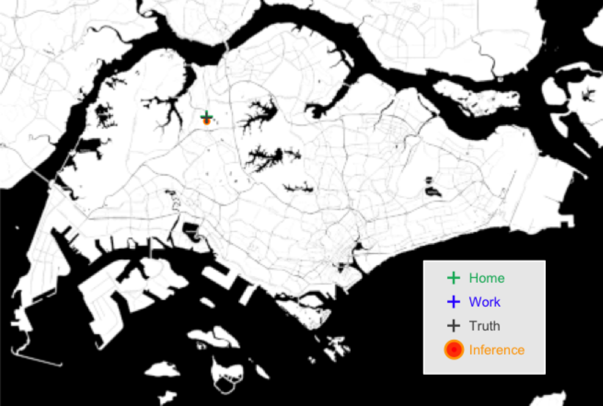
Home
Work
Truth
Inference

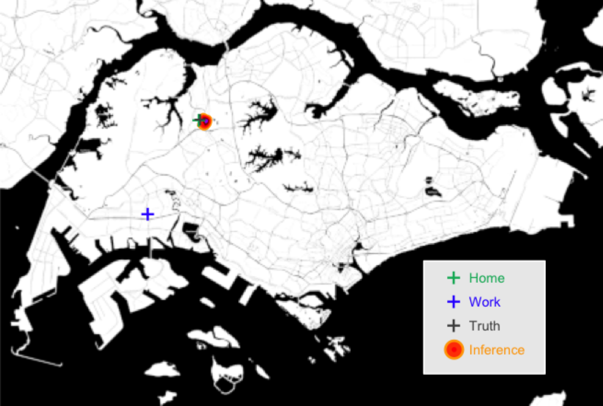
Home
Work
Truth
Inference

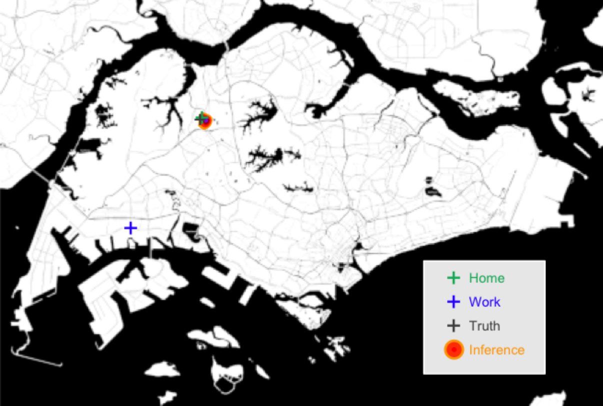
Home
Work
Truth
Inference

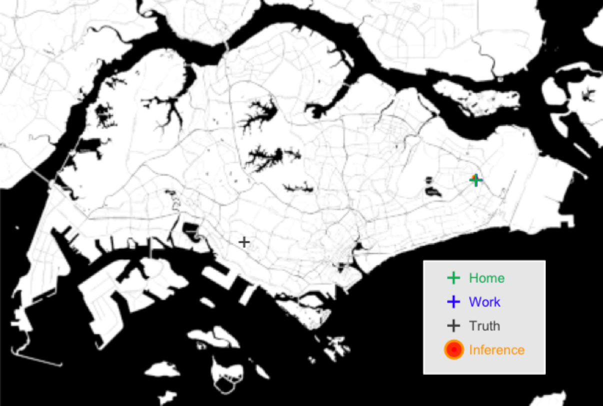
Home
Work
Truth
Inference

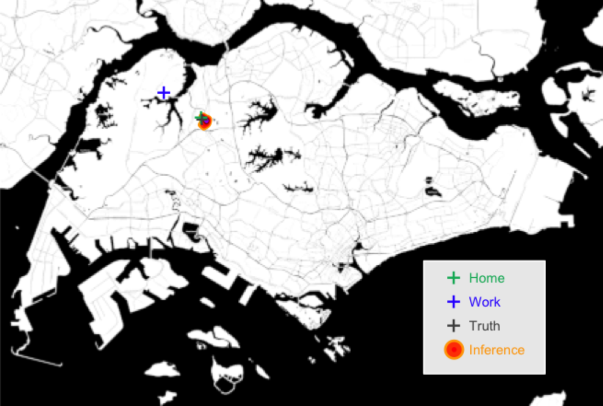
Home
Work
Truth
Inference

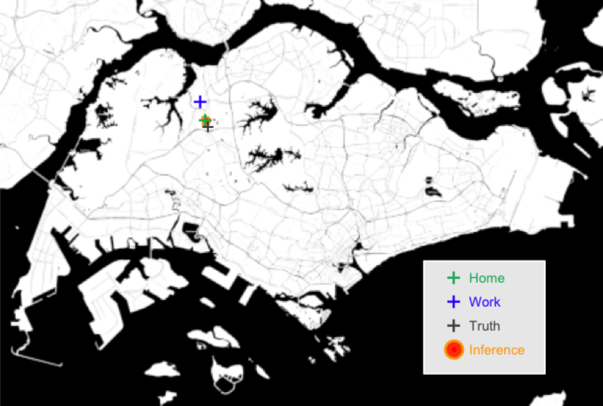
Home
Work
Truth
Inference

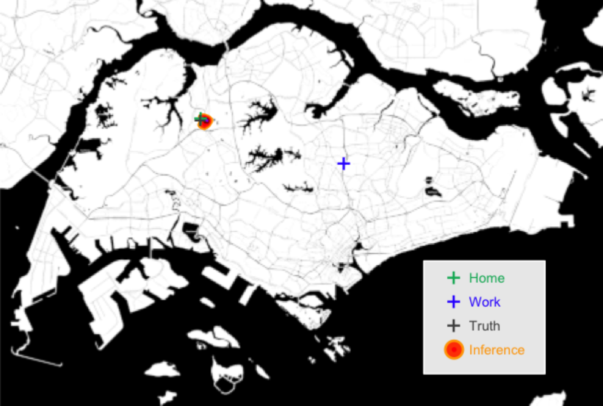
Home
Work
Truth
Inference

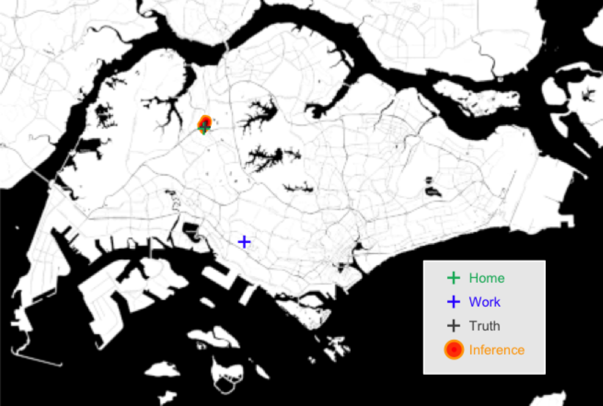
Home
Work
Truth
Inference

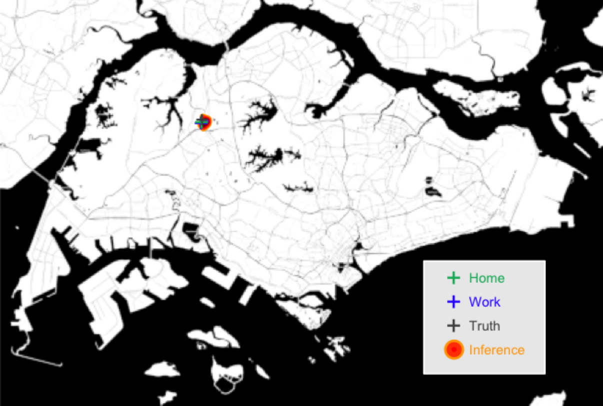

Home
Work
Truth
Inference

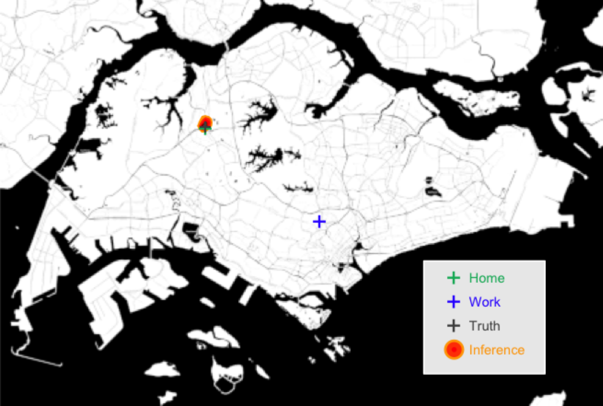
Home
Work
Truth
Inference

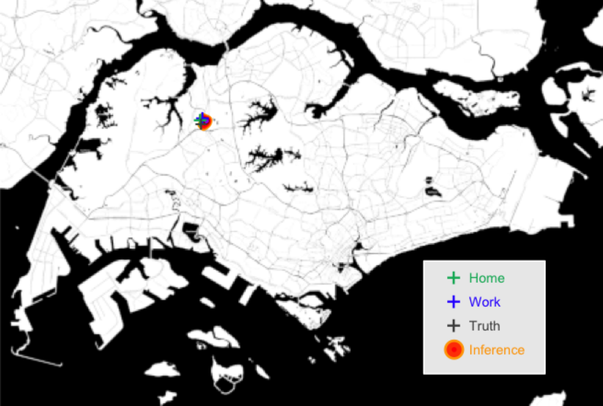
Home
Work
Truth
Inference

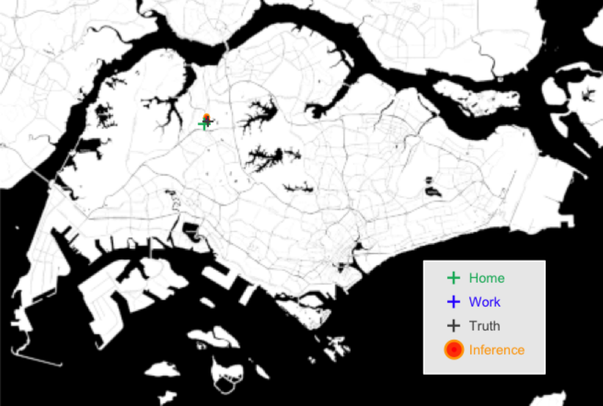
Home
Work
Truth
Inference

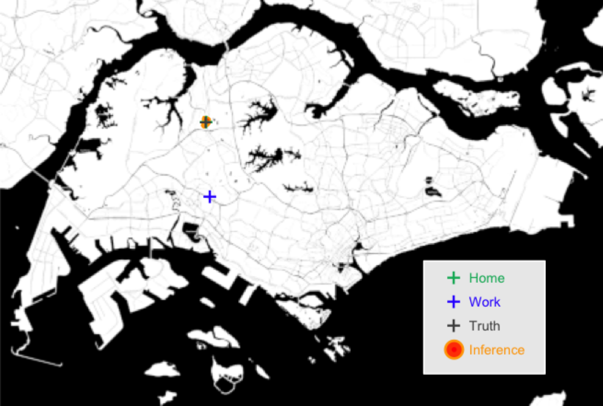
Home
Work
Truth
Inference

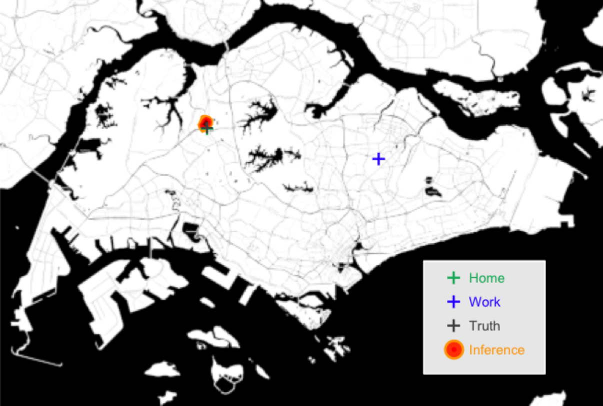
Home
Work
Truth
Inference

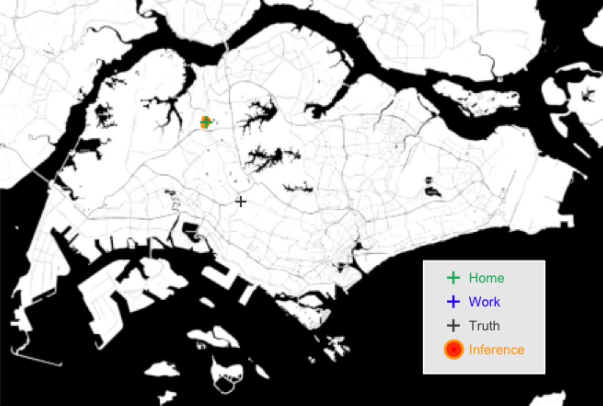
Home
Work
Truth
Inference

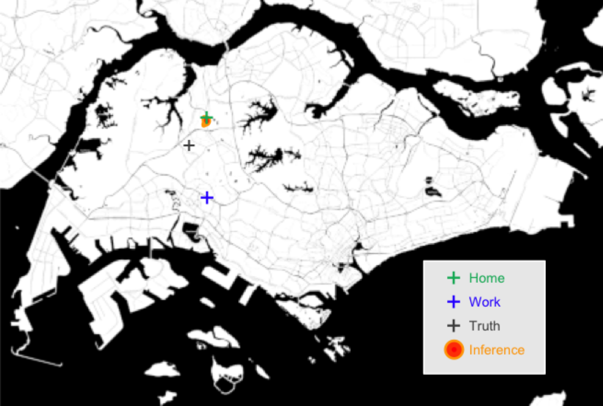
Home
Work
Truth
Inference

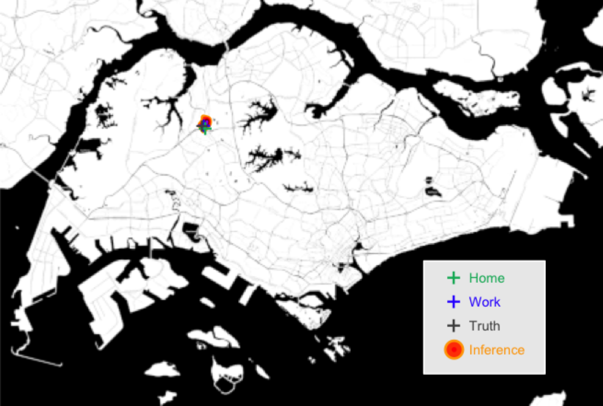
Home
Work
Truth
Inference

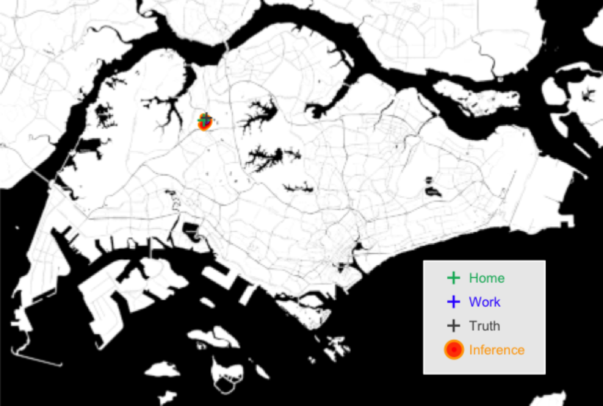
Home
Work
Truth
Inference

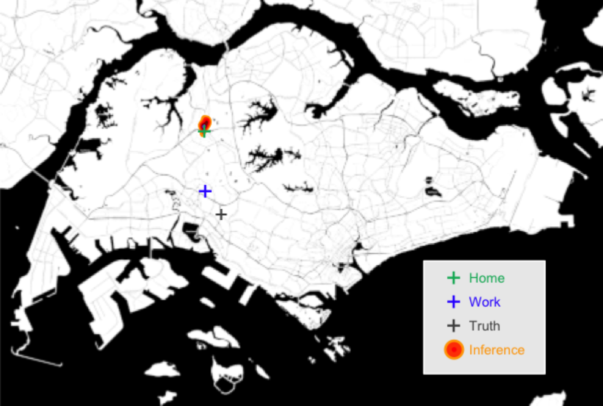
Home
Work
Truth
Inference

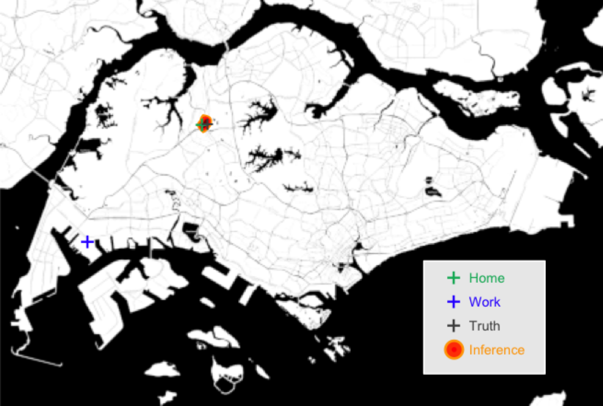

Home
Work
Truth
Inference

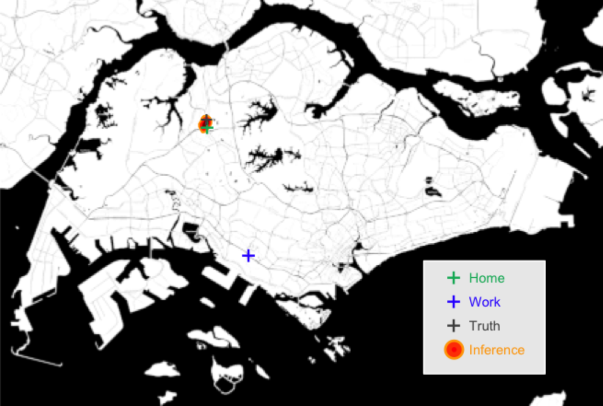
Home
Work
Truth
Inference

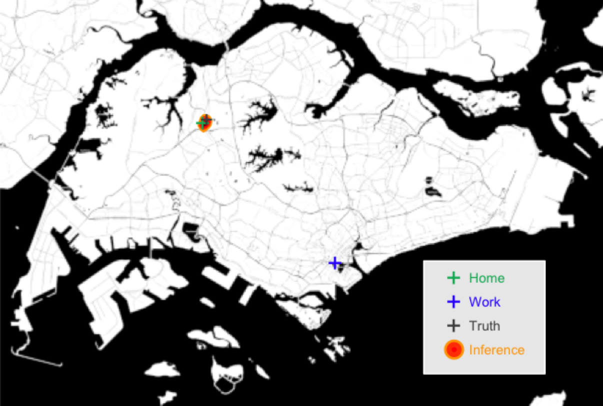
Home
Work
Truth
Inference

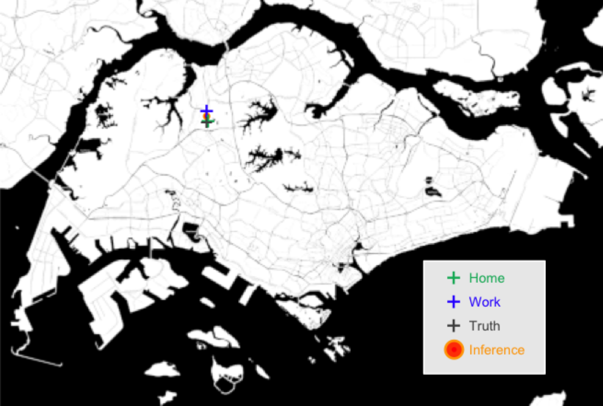
Home
Work
Truth
Inference

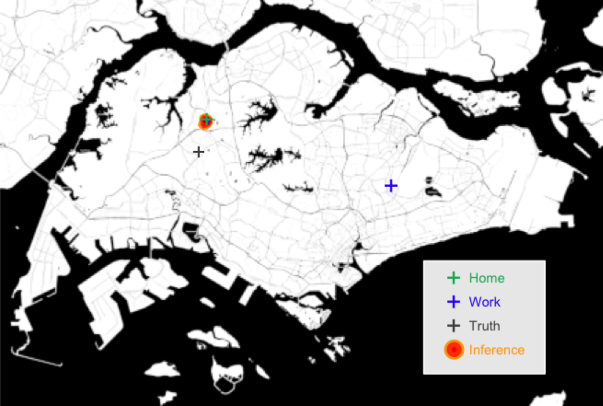

Home
Work
Truth
Inference

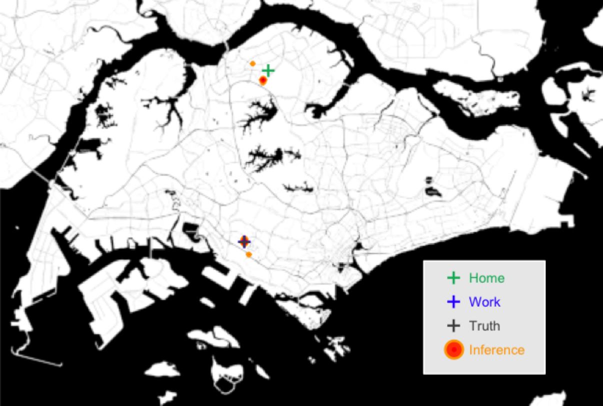
Home
Work
Truth
Inference

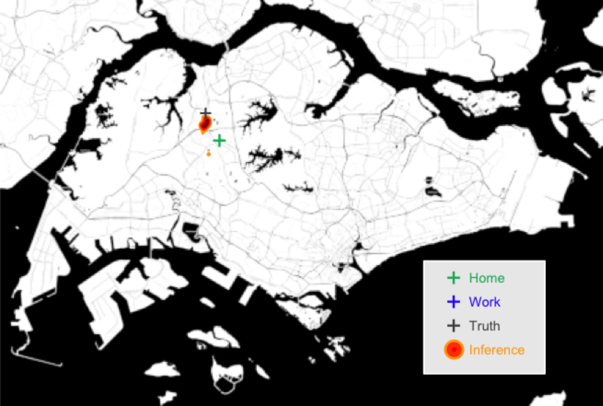
Home
Work
Truth
Inference

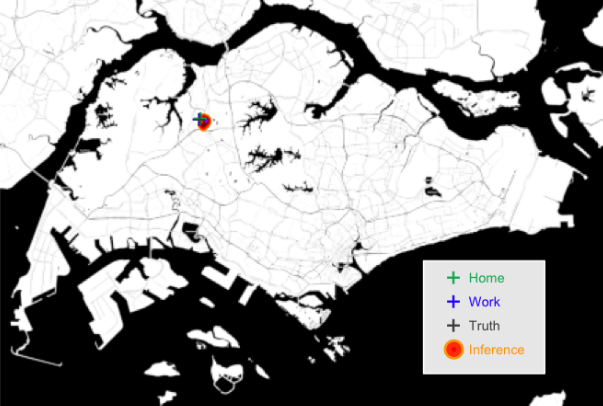

Home
Work
Truth
Inference

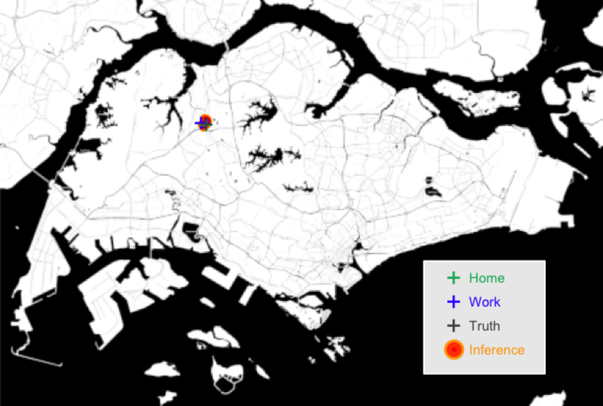
Home
Work
Truth
Inference

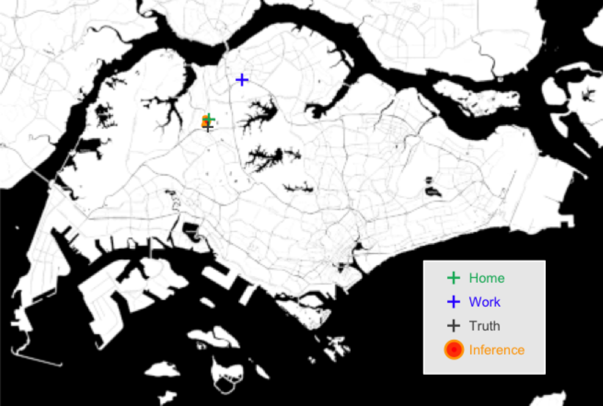
Home
Work
Truth
Inference

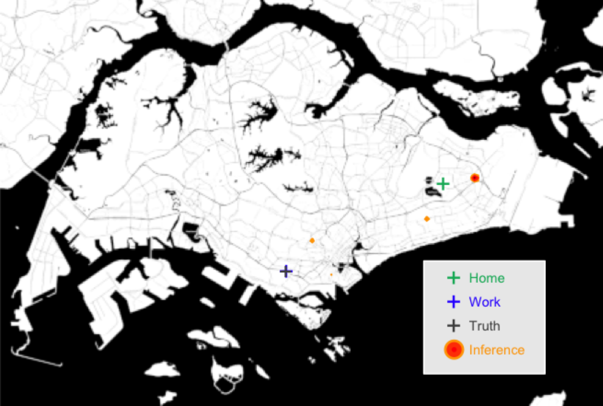
Home
Work
Truth
Inference

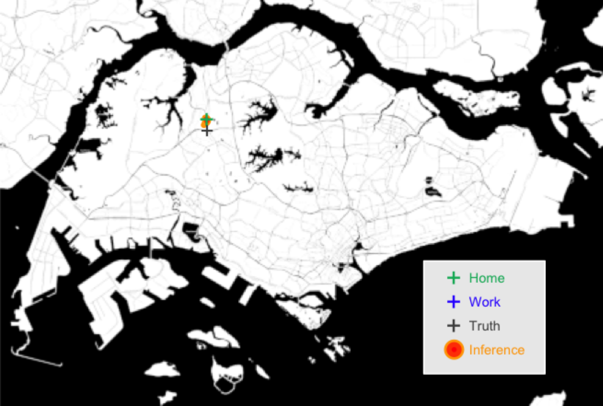
Home
Work
Truth
Inference

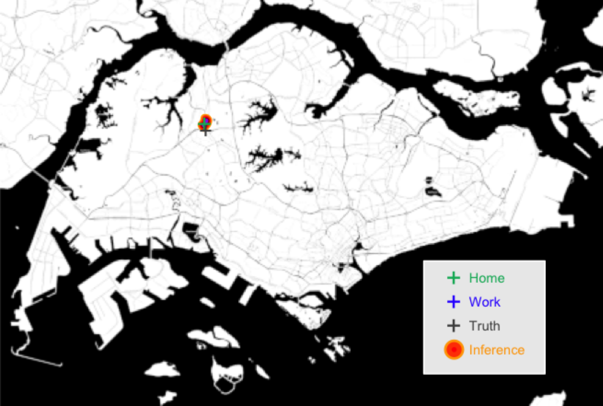
Home
Work
Truth
Inference

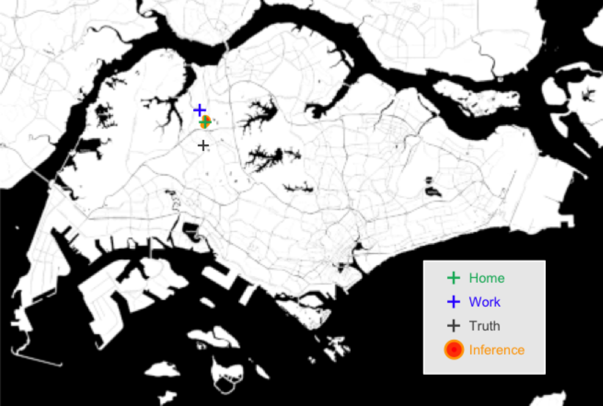
Home
Work
Truth
Inference

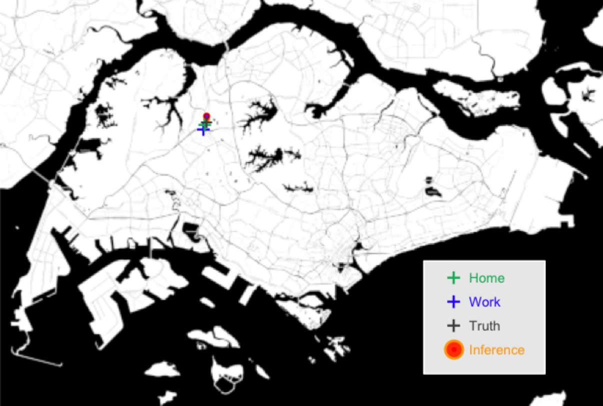
Home
Work
Truth
Inference

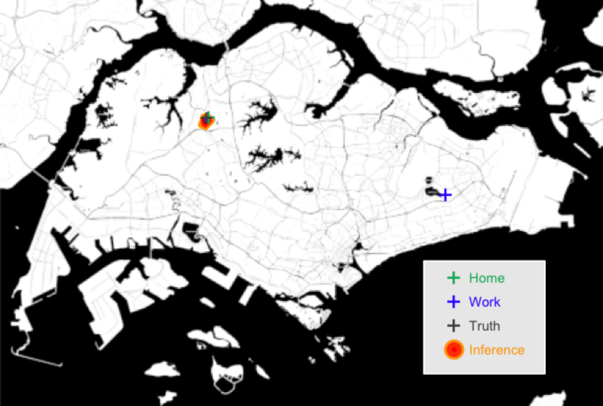

Home
Work
Truth
Inference

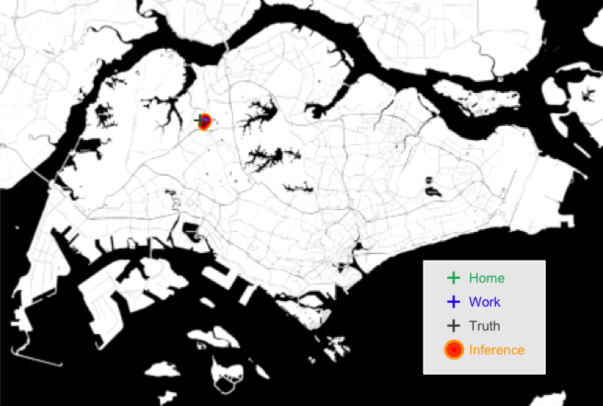

Home
Work
Truth
Inference

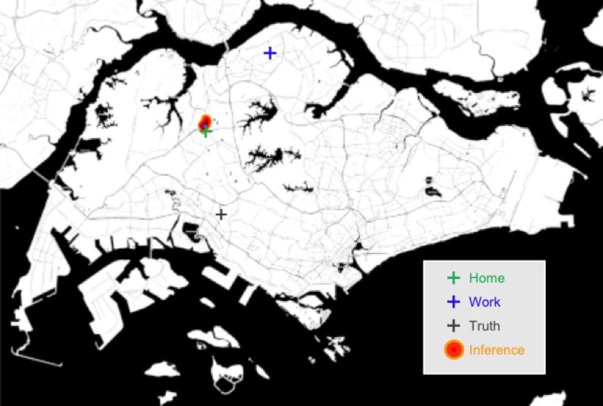
Home
Work
Truth
Inference

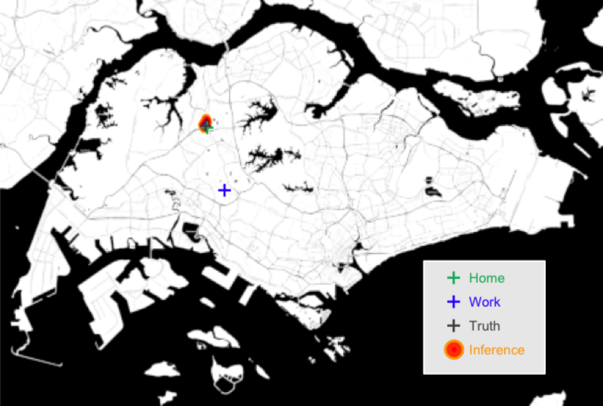
Home
Work
Truth
Inference

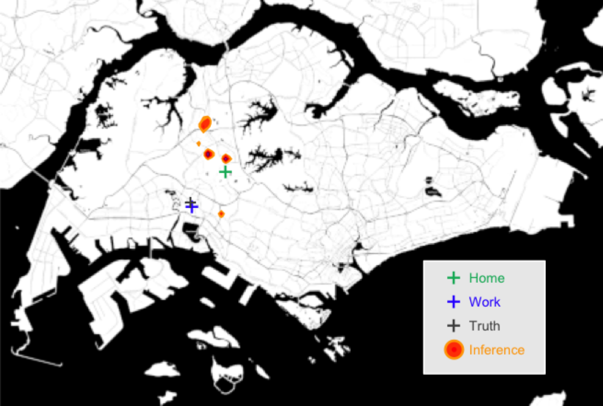
Home
Work
Truth
Inference

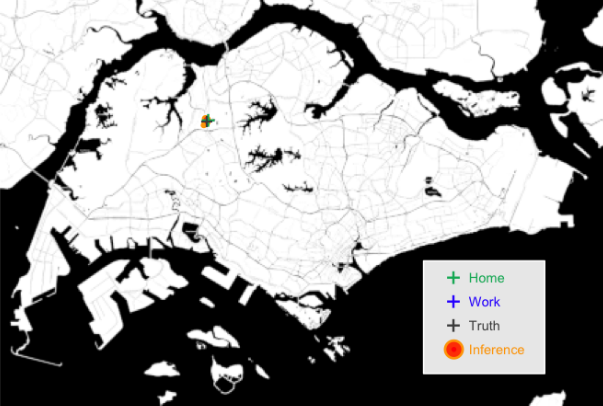
Home
Work
Truth
Inference

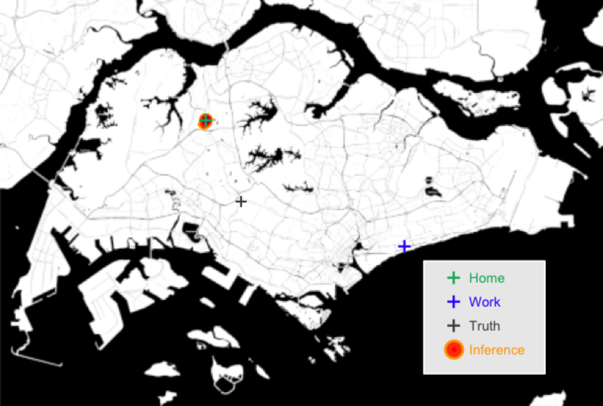

Home
Work
Truth
Inference

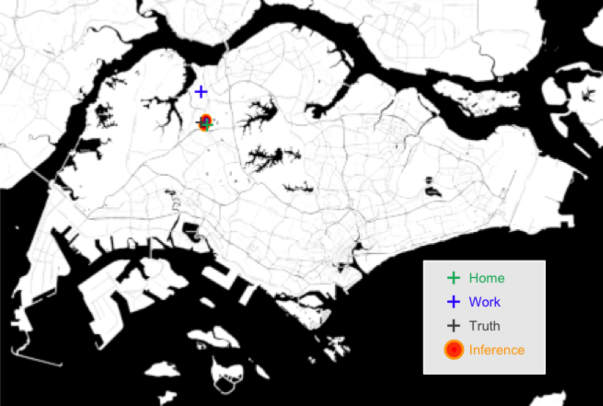
Home
Work
Truth
Inference

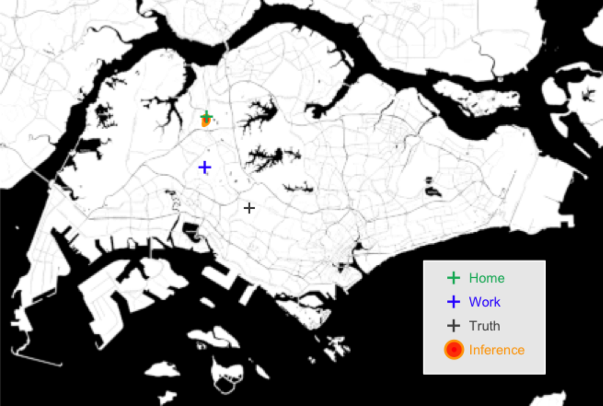
Home
Work
Truth
Inference

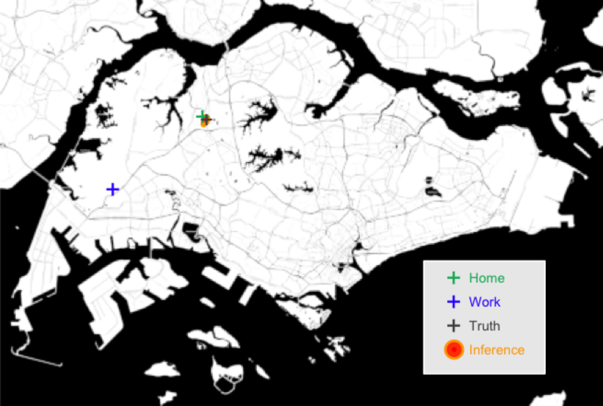
Home
Work
Truth
Inference
